# Design, Synthesis, Molecular Modeling, and Biological Evaluation of Novel Pyrimidine Derivatives as Potential Calcium Channel Blockers

**DOI:** 10.3390/molecules28124869

**Published:** 2023-06-20

**Authors:** Yasser M. Zohny, Samir M. Awad, Maha A. Rabie, Omar Awad Alsaidan

**Affiliations:** 1 Pharmaceutical Sciences Department, College of Pharmacy, Shaqra University, Dawadmi 11911, Saudi Arabia; 2 Pharmaceutical Organic Chemistry Department, Faculty of Pharmacy, Helwan University, Cairo 11795, Egypt; samirawad2000@yahoo.com; 3 Pharmacy Practice Department, College of Pharmacy, Shaqra University, Dawadmi 11911, Saudi Arabia; maha.rabie@su.edu.sa; 4 Pharmacology and Toxicology Department, School of Pharmacy, Cairo University, Cairo 11795, Egypt; 5 Department of Pharmaceutics, College of Pharmacy, Jouf University, Sakaka 72341, Saudi Arabia; osaidan@ju.edu.sa

**Keywords:** 3,4-dihydropyrimidin-2(1H)ones, nifedipine isosters, antihypertensive, calcium channel blocking

## Abstract

Pyrimidines play an important role in modern medical fields. They have a wide spectrum of biological activities such as antimicrobial, anticancer, anti-allergic, anti-leishmanial, antioxidant agents and others. Moreover, in recent years, 3,4-dihydropyrimidin-2(1H)ones have attracted researchers to synthesize them via Biginelli reaction and evaluate their antihypertensive activities as bioisosters of Nifedipine, which is a famous calcium channel blocker. Our new target compounds were prepared through one-pot reaction of thiourea **1**, ethyl acetoacetate **2** and/or 1H-indole-2-carbaldehyde, 2-chloroquinoline-3-carbaldehyde, 1,3-diphenyl-1H-pyrazole-4-carbaldehyde, **3a**–**c** in acid medium (HCl) yielding pyrimidines **4a**–**c**, which in turn were hydrolyzed to carboxylic acid derivatives **5a**–**c** which were chlorinated by SOCl_2_ to give acyl chlorides **6a**–**c**. Finally, the latter were reacted with some selected aromatic amines, namely, aniline, p-toluidine and p-nitroaniline, producing amides **7a**–**c, 8a**–**c,** and **9a**–**c**. The purity of the prepared compounds was examined via TLC monitoring, and structures were confirmed by different spectroscopic techniques such as IR, ^1^HNMR, ^13^CNMR, and mass spectroscopy. The in vivo evaluation of the antihypertensive activity revealed that compounds **4c, 7a, 7c, 8c, 9b** and **9c** had comparable antihypertensive properties with Nifedipine. On the other hand, the in vitro calcium channel blocking activity was evaluated by IC_50_ measurement and results revealed that compounds **4c, 7a, 7b, 7c, 8c, 9a, 9b,** and **9c** had comparable calcium channel blocking activity with the reference Nifedipine. Based on the aforementioned biological results, we selected compounds **8c** and **9c** to be docked onto Ryanodine and dihydropyridine receptors. Furthermore, we developed a structure–activity relationship. The designed compounds in this study show promising activity profiles in reducing blood pressure and as calcium channel blockers, and could be considered as new potential antihypertensive and/or antianginal agents.

## 1. Introduction

Hypertension is a long-term medical condition in which the blood pressure in the arteries is persistently elevated; it can lead to heart diseases, stroke and death and is a major global health concern [1,2]. There are several types of drugs used to treat high blood pressure such as ACE-inhibitors, diuretics, ß-blockers, α-blockers, renin inhibitors, calcium channel blockers and many more [3,4,5]. Calcium channel blockers (CCBs) are the drugs which slow down the movement of calcium into the cells of the walls of heart and blood vessels, making it easier for the heart to pump the blood and widen blood vessels [6,7]. They also alter the heart rate to prevent peripheral and cerebral vasospasm and reduce chest pain caused by angina pectoris. CCBs are easily identified by the suffix ‘‘dipine’’ such as Amlodipine, Arnidipine, Barnidipine, Clindipine, Isradipine and Manidipine [8,9,10]. Nifedipine is one of the most common and classic CCBs and is a first-generation dihydropyridine, often used to reduce systemic vascular resistance and arterial pressure [11,12]. However, Nifedipine is proven to be short acting and is no longer appropriate for the treatment as it causes unpredictable falls in blood pressure and may precipitate in ischemic events [13]. Hence, some bioisosters of Nifedipines and pyrimidines were synthesized and screened for antihypertensive activity by several researchers [14,15].

**Scheme 1:**
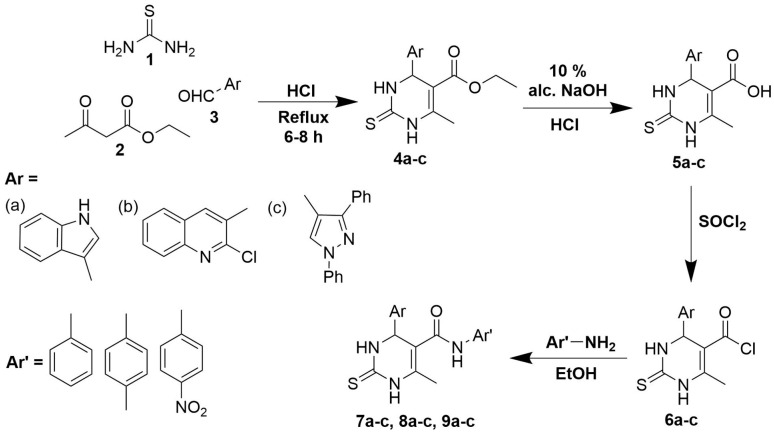
Synthesis of target dihydropyrimidines with three different starting aldehydes (a) 1H-indole-3-carbaldehyde; (b) 2-chloroquinoline-3-carbaldehyde and (c) 1,3-diphenyl-1H-pyrazole-4-carbaldehyde.

CCBs are classically synthesized by Biginelli reaction with urea, ethyl acetoacetate and aldehydes as starting materials, in an acid medium [16,17]. We revisited the large amount of literature [18,19] and the concluding remarks of our previous review article on Biginelli reactions for CCB synthesis [20]; herein, we propose a synthesis of series of novel CCBs, which are isosters of Nifedipine using the three-pot Biginelli reaction. Thiourea 1 and ethyl acetoacetate **2** and three varied aromatic aldehydes, 1H-indole-2-carbaldehyde, 2-chloroquinoline-3-carbaldehyde, 1,3-diphenyl-1H-pyrazole-4-carbaldehyde were used as starting materials. Target compounds **4** to **9** (as in Scheme 1) were synthesized and subjected to biological evaluation towards antihypertensive activity and calcium channel blocking ability. Relying on the results, the best target compounds were further considered for molecular modelling and docking experiments using ryanodine and dihydropyridine receptors to predict the structure–activity relationships.

## 2. Results and Discussions

### 2.1. Chemistry

The synthetic route for DHTM compounds is presented in Scheme 1. Fused DHPs such as hexahydroquinolines, indenopyridines and acridines are proven to be the active derivatives, exhibiting calcium antagonistic effects [21,22]. It has been demonstrated in the past that the substitution at the hexahydroquinoline ring’s six-position affects the sensitivity of the L-type channel inhibition [23]. Moreover, the substitution with simple phenyl rings are preferred over aromatic systems with heteroatoms due the animal toxicity [24,25]. Additionally, the analogs have exposed that the biological activity depends on the hydrophilic, lipophilic, electronic and steric properties of the substituents [26]. As provided in Scheme 1, the DHPM analogues with hybrid structures having a combination with indole, quinolone and pyrazole structures were designed to have versatile structures, while keeping the core skeleton structure intact. The variants were targeted by having different substitutions at the C5 position, and the aldehydes for the targets were selected according to the electron affinity and polarity of the substituents. In the present study, the Biginelli condensation reaction was performed with usual three component system, wherein, the thiourea, ethyl acetoacetate and selected heterocyclic aldehydes namely, 1H-indole-3-carbaldehyde, 2-chloroquinoline-3-carbaldehyde and 1,3-diphenyl-1H-pyrazole-4-carbaldehyde were selected as starting materials to offer corresponding 1,4-dihydropyrimidine esters **4a**–**c.** The formation of compounds 1,4-dihydropyrimidine esters was confirmed by the appearance of CH aromatic carbon at 3173 cm^−1^ and NH bands at 3426 and 3319 cm^−1^ from FT-IR spectra and the multiplets for aromatic protons from ^1^H NMR. The skeleton structure presented similar proton peaks for all three; however, the considered three aldehydes (Ar groups) presented different peaks in NMR. 1H-indole-3-carbaldehyde (compound 4c) presented extra peaks for 5H, 4H from the six-membered ring and a peak at 7.66 ppm was assigned for the aromatic proton of the five-membered heterocyclic ring. The saponification of ethyl ester provided the sodium salts of 1,4-dihydropyrimidine, the acidification of which to pH ≈ 4 yielded corresponding acids **5a**–**c,** which is substantiated by the absence of a proton peak at 1.18 ppm and the carbon peaks at ~14 ppm. The acyl chloride analogues, **6a**–**c** were obtained by chlorination of carboxylic acid derivatives using thionyl chloride which was conferred by the chemical shift from 169 to 178 ppm under ^13^C spectra of **6a**–**c** compounds. The resulting acyl chloride derivatives were further reacted with three aromatic amines, namely, aniline, *p*-toluidine and *p*-nitroaniline, to obtain the target 1,4-dihydropyrimidinethionecarboxamide novel derivatives **7a**–**c, 8a**–**c,** and **9a**–**c,** which are reported for the first time as per our knowledge. NMR of compound 4c appeared with two tt, and dtd in the aromatic proton range confirming diphenyl-1H-pyrazole moiety. The disappearance of triplet ~*δ* 1.24 (3H, t, J = 7.1 Hz) for the compounds 5a–c indicates the conversion of the ester to the acid group. The final compounds 7a–c, 8a–c and 9a–c presented extra aromatic peaks compared with 6a–c, pertaining to the Ar’ group. TLC in different solvent systems and the measured melting points confirmed the purity of obtained compounds, and the structural confirmation was conducted by analytical as well as spectral analysis.

### 2.2. Biological Evaluation

#### 2.2.1. Evaluation of Antihypertensive Activity

**Figure 1:**
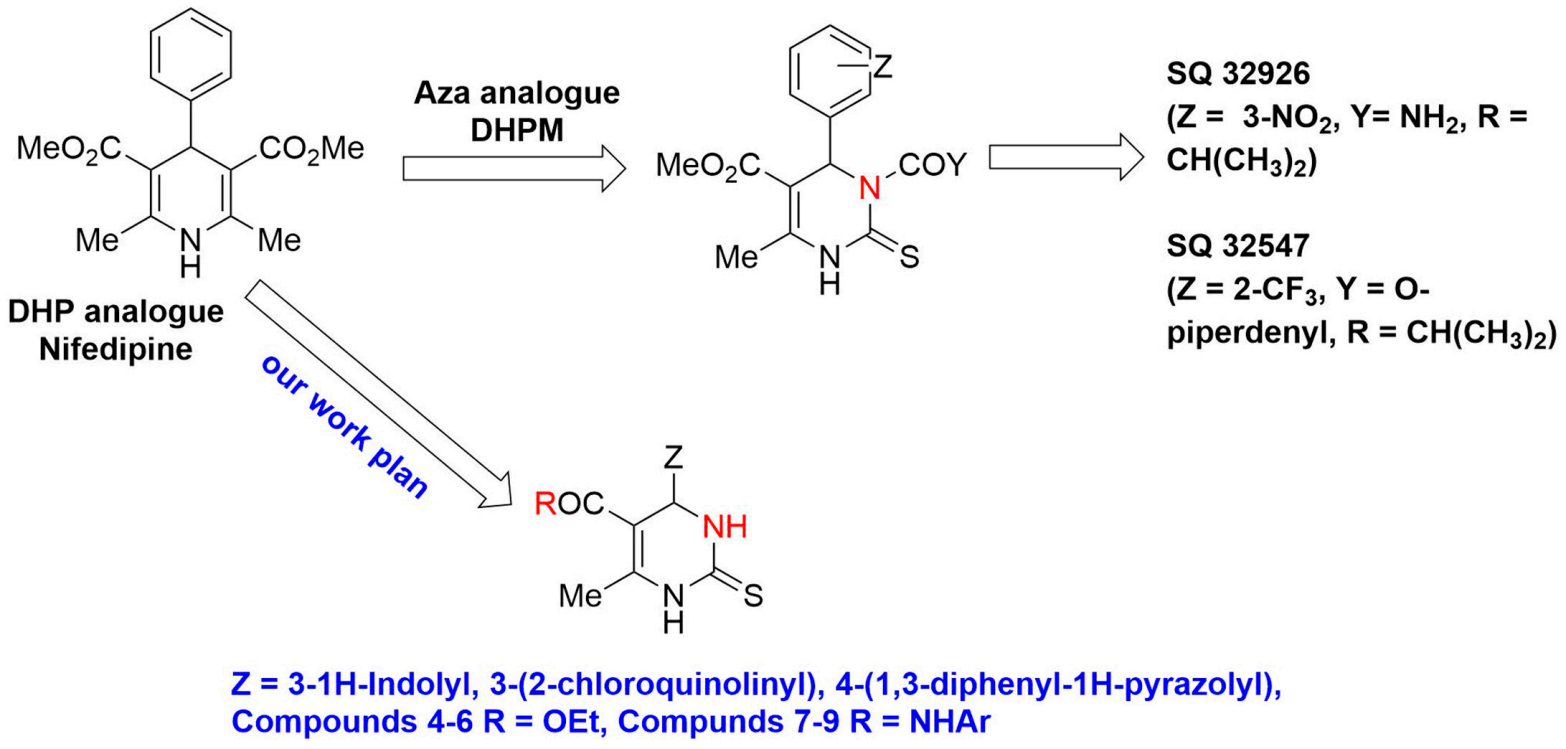
DHP analogue and aza analogues.

The analogues of pyrimidines and DHPMs are known to treat hypertension [14,27,28]. There are several aza analogues of DHPM, which were synthesized as shown in Figure 1. The compounds SQ 32926 and SQ 32547, in addition to many other pyrimidines analogues, have demonstrated more oral activity and potency than DHP antihypertensive drugs, which need longer time for treating antihypertension [29,30]. The current study highlights the synthesis of bioisosters of Nifedipine having similar nuclei as shown in Figure 1, and their biological evaluation towards antihypertensive activity and CCB activity with an aid of Nifedipine as a reference standard. 

The antihypertensive screening for the target compounds (**4**–**9**) was investigated by measuring the % inhibition in the blood pressure and the results are tabulated in Table 1. Nifedipine displayed a % inhibition of blood pressure of 31.32 and 26.54, which decreased to the compounds **4a**, **4b**, **5a**, **5b**, **5c**, **6a**, **6b**, **6c**, **7b**, **8a**, **8b** and **9a** (the range is between 11 and 23); the exact figure facts are provided Table 1. Among all the screened compounds, the compounds **4a**, **7a**, **7c**, **8c**, **9b** and **9c** presented an adjacent value to Nifedipine.

#### 2.2.2. Evaluation of Calcium Channel Blocking Activity

According to the literature, the contraction of smooth muscles depends on the calcium influx [14,31], though their underlying mechanism is different, the blocking of influx of calcium channels to smooth muscles by CCBs causes relaxation. To investigate the antagonistic effects of target compounds, the potassium chloride induced contractions were assessed. The measured % inhibition in the contraction at different doses for all the target compounds along with IC_50_ values are provided in Table 2. As expected from antihypertensive activity, the compounds **4a**, **4b**, **5a**, **5b**, **5c**, **6a**, **6b**, **6c**, **7b**, **8a**, **8b** and **9a** presented the % of inhibition in the contraction according to the standard drug Nifedipine. However, for all other drugs, the contraction % increased, and a similar trend was observed for IC_50_ values as well. For all the compounds with increased doses of compound, the % inhibition in the contraction increased; hence, it is said to be dose-dependent. The standard drug gave an IC_50_ of 21 μg/mL, and the smallest IC_50_ was observed for compound **8c**.

For a better understanding, the results were compared with a previous investigation by one of the authors of this manuscript [32]. The author and group synthesized DHPM derivatives and investigated both the antihypertensive and CCB activity amongst other activities. Aromatic substituents were employed at the C_4_ position of the DHPM ring to afford three compounds (1,4 dihydropyrimidinethione carboxamide derivatives) showing a result close to that of standard Nifedipine (IC50 21.00 ± 1.20 μg/mL), varying from 19.65 ± 1.60 to 21.45 ± 2.55 μg/mL. In the present study, the replacement of such aromatic substituents by heterocyclic ones seemed to slightly enhance both the antihypertensive and the CCB activity.

*Moreover*, *N3*-substituted DHPMs were synthesized by Mohamed Teleb et al. [33]. Among synthesized DHPMs, the ones with ester groups demonstrated better CCB activity, which was due to the improved pharmacokinetic activity achieved by stability towards metabolic inactivation. Nevertheless, the selectivity was achieved by having methoxy moiety and triazole ligation to the ester group. The presence of acid and amide groups decreased the CCB activity. In addition, the substitution at the C_4_ position with a phenyl ring carrying several moieties such as 2-Cl, 3-Cl, 2-CH_3_, 2-OC_2_H_5_, NO_2_ and many more [31], mentioned derivatives improved the antispasmodic effect and vasodilator activity, except the alkyl groups, with no electron withdrawing or releasing effects. 

**Figure 2:**
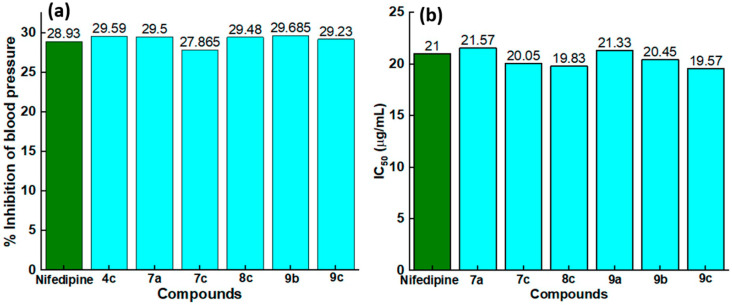
Comparison of (**a**) antihypertensive activity in terms of percent inhibition in blood pressure, (**b**) CCB in terms of IC_50_ values between the standard drug and the target compounds showing similar efficacy to that of standard.

For a better understanding, the synthesized compounds showing an efficacy much closer to the standard drug Nifedipine in terms of hypertensive activity (Figure 2a) and CCB activity (Figure 2b) are graphed. Compounds **4c, 7a, 7c, 8c, 9b** and **9c** showed a % inhibition in the blood pressure varying from 27.865 to 29.6 %, whereas Nifedipine showed 28.93 %. In the case of CCB activity measurements, a lower IC_50_ was obtained for compounds 8c and 9c (about 19 μg/mL), which is less than that of the standard drug.

### 2.3. Molecular Modeling and Binding Mode Prediction

**Figure 3:**
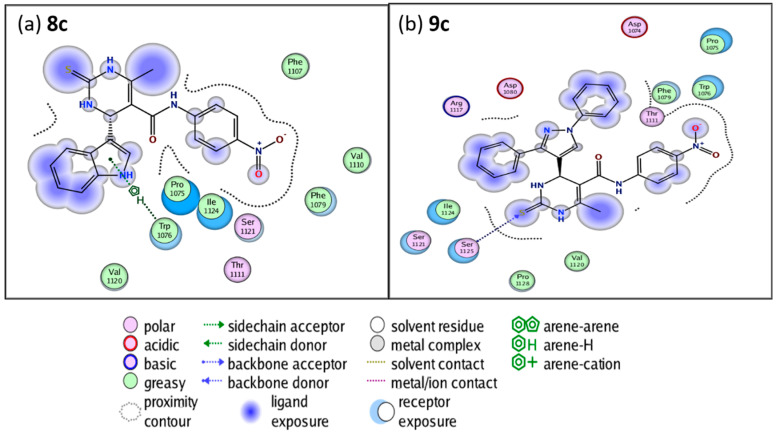
The 2D visualization of (**a**) compound **8c** and (**b**) compound **9c** ligand interaction with dihydropyridine using the MOE 2015.10th version software.

**Figure 4:**
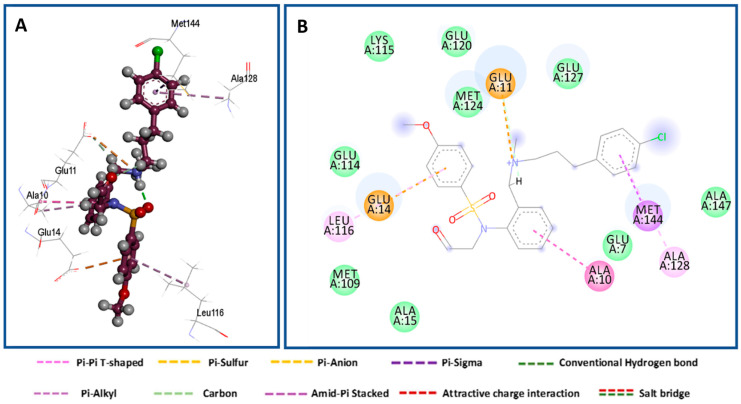
Docking mode of co-crystalized ligand (KN93). (**A**) A 3D interaction map between co-crystalized ligand and binding sites. (**B**) A 2D molecular docking model of the co-crystalized ligand.

**Figure 5:**
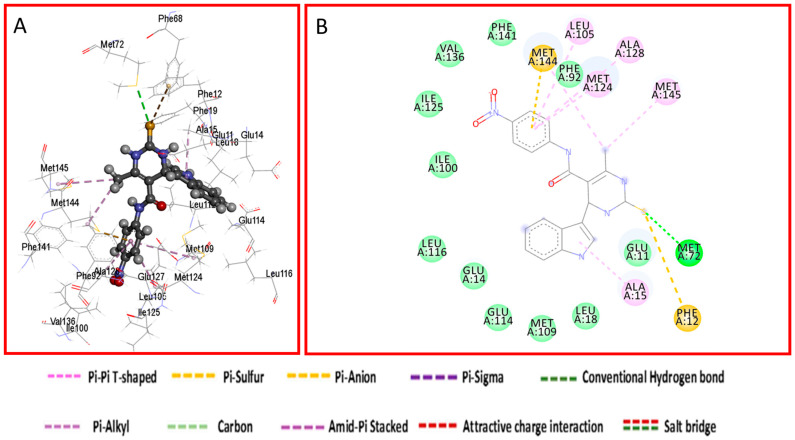
Docking mode of compound **8c**. (**A**) A 3D interaction map between compound 8c and binding sites. (**B**) A 2D molecular docking model of compound **8c**.

**Figure 6:**
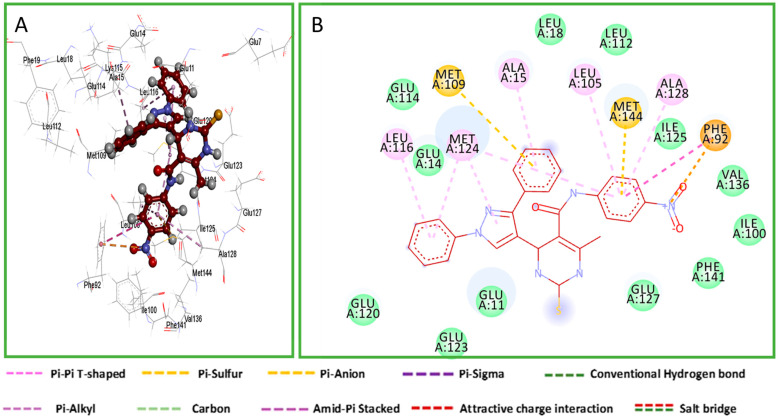
Docking mode of compound **9c**. (**A**) A 3D interaction map between compound 9c and binding sites. (**B**) A 2D molecular docking model of compound **9c**.

The work uses Nifedipine in the biological experiment as it is the drug used in the market for long and compared with present work. Both KN93 and Nifedipine produce similar pharmacological effects [34]. However, KN93 share some interesting similarities with our compounds. Structurally, KN93 contains three hydrophobic rings as our target compounds which are similarly spaced apart. These resemble hydrophobic side chains of amino acids that CaM often interacts with [35]. The antihypertensive and calcium channel blockade activity results proved that compounds **8c** and **9c** were promising, with IC_50_ values of 19.83 and 19.57 μg/mL, and hence employed for molecular modelling studies to understand the binding mode and structure–activity relationships. Each of two compounds and co-crystalized ligand, KN93, were docked into the active site of the Ryanodine receptor using Molecular Operating Environment (MOE) software (10th version). The binding affinities were evaluated on the basis of the binding free energy S-score and hydrogen bonds with their distance between the designed compounds and the amino acids in the receptor (Table 3). Figure 3 provides the 2D visualization of the Met 144 residue of the Ryanodine receptor and the binding affinities with compound **8c** and **9c.** There existed several interactive catalytic sites, such as amide-pi stacked with the chlorobenzene ring of KN93, pi-cation interaction with benzene ring of both the compounds **8c** and **9c**. A total of 15 residues were found at the binding of the Ryanodine–KN93 complex, whereas 20 were seen in the Ryanodine–Nifedipine complex. In our compounds, 19 residues were seen in the Ryanodine–8c complex. Finally, a total number of 20 residues were shown in the Ryanodine–9c complex. The molecular docking modes of co-crystalized ligand and the interaction map between the co-crystallized ligand are presented in Figure 4, Figure 5 and Figure 6.

### 2.4. Structure–Activity Relationships

As described in Table 3. The analysis of the structure–activity relationships shows that the combining of different aromatic/heterocyclic rings to 6-methyl-2-thioxo-1,2,3,4-tetrahydropyrimidin-5-yl)methanone moiety contributed to variation in the calcium channel blocker activity of these compounds. The addition of 1,3-diphenyl-1H-pyrazole ring and 4-nitrophenyl ring to 6-methyl-2-thioxo-1,2,3,4-tetrahydropyrimidin-5-yl)methanone moiety afforded compounds **9c** and **8c** that are more potent than the addition of phenyl group, 4-methyl phenyl ring and 2-chloroquinoline ring to 6-methyl-2-thioxo-1,2,3,4-tetrahydropyrimidin-5-yl)methanone moiety to give compounds **9a, 7a, 8b** and **9b**. 4-nitro phenyl ring, compound **9c** is the most active compound followed by 4-methyphenyl ring, compound **9b** and phenyl ring, compound **9a** as a calcium channel blocker. The presence of methyl phenyl ring in compound **9b** is more effective than the presence of phenyl ring in compound **9a** and also the existence of 4-nitro phenyl moiety in compound **8c** is more effective than the presence of 4-methylphenyl ring in compound **8b**. The docking studies for compounds 8c and 9c presented the better binding score than the standard Nifedipine and the results established the importance of the existence of methylphenyl, phenyl, and nitrophenyl rings as pharmacophores for the calcium channel blocker activity.

## 3. Materials and Methods

### 3.1. Chemistry

#### Materials and Methods

The reagents used in the study were procured from Merck, Darmastadt, Germany. No prior purification was carried out, and all the reagents were used as such. The melting points for the target compounds were measured with Electro-thermal IA 9100 apparatus (Shimadzu, Japan). The proton NMR spectra were measured with Bruker AMX400 and Bruker Current AV400 Data spectrometer (400 MHz), Bruker BioSpin GmbH, Germany. Trimethylsilane (TMS) is used as internal reference and the alterations in chemical shifts, *δ* were uttered in ppm. Electrospray Ionization (ESI) spectra were measured using Finnigan Thermo Quest MAT 95XL spectrometer and FAB high-resolution (HR) mass spectra with a VG Analytical 70-250S spectrometer; Palmer, USA with method, MCA and polyethylene glycol (PEG) as support. The reactions were monitored by Thin Layer Chromatography (TLC) with a silica gel (60 F254) coated with aluminum plates, Merck, and are envisioned by UV irradiation and iodine vapors. Silica gel of mesh size 60–120 mesh was used for column chromatography. The reactions were performed under an atmosphere of dry nitrogen. 

Thiourea **1**, (7.6 g, 0.1 mol), ethyl acetate **2**, (13 mL, 0.1 mol) and the appropriate aromatic/heterocyclic aldehyde **3** (0.1 mol), were taken in 50 mL absolute ethanol containing 1 mL of 37% HCl and was refluxed for 8 h. The reaction mixture was then brought to room temperature and poured into an ice/water mixture and neutralized with ammonia solution. The obtained precipitate was filtered and dried using suction and recrystallized with ethanol.

**4a:** *Ethyl 4-(1H-indol-3-yl)-6-methyl-2-thioxo-1,2,3,4-tetrahydropyrimidine-5-carboxylate:* Yield: 76%; m.p.: 252–254 °C; IR ν (KBr cm^−1^): 3426 (NH), 3333 (NH), 3319 (NH), 3173 (CH, aromatic), 2978 (CH, aliphatic), 1749 (C=O ester), 1683 (C=O), 1277 (C=S), 1220 (C–O). ^1^HNMR (DMSO-*d*_6_, 400 MHz) *δ*: 1.19 (t, 3H, CH_3_), 2.1 (s, 3H, CH_3_), 3.76 (q, 2H, CH_3_CH_2_-O), 5.67 (s, 1H, CH), 7.28–8.05 (m, 5H, aromatic), 10.34, 10.51, 11.12 (3 s, 3H, 3NH (D_2_O exchangeable)). ^13^C NMR: (DMSO-*d*_6_, 400 MHz) *δ* (ppm) 177.4 (1C, s), 161.6 (1C, s), 154.9, 146.0 (1C, s), 141.0, 139.67 (1C, s), 130.6 (1C, s), 128.1–128.3 (2C, 128.2 (s), 128.2 (s)), (1C, s), 113.4 (1C, s), 60.0 (1C, s), 52.1, (1C, s), 16.7 (1C, s), 14.9 (1C, s); MS (EI) *m*/*z*: 315.11 (M+, 12.5%); Calcd./Anal., for C_16_H_17_N_3_O_2_S: C, 60.93; H, 5.43; N, 13.32. Found: C, 60.68; H, 5.27; N, 13.66.
**4b:** *Ethyl 4-(2-chloroquinolin-3-yl)-6-methyl-2-thioxo-1,2,3,4-tetrahydropyrimidine-5-carboxylate***:** Yield: 79%; m.p.: 218–220 °C; ^1^HNMR (DMSO-*d*_6_, 400 MHz) *δ*: 1.18 (t, 3H, CH_3_), 2.39 (s, 3H, CH_3_), 3.67 (q, 2H, CH_3_CH_2_-O), 5.4 (s, 1H, CH), 7.36–8.09 (m, 5H, aromatic), 10.09, 11.33 (2 s, 2H, 2NH (D_2_O exchangeable)). ^13^C NMR: (DMSO-*d*_6_, 400 MHz) *δ* (ppm) 178.1 (1C, s), 161.6 (1C, s), 154.7 (1C, s), 147.6 (1C, s), 146.0 (1C, s),134.7 (1C, s), 128.5 (1C, s), 128.3 (1C, s), 127.4 (1C, s), 126.0 (1C, s), 125.5 (1C, s), 121.7 (1C, s), 113.4 (1C, s), 60.0 (1C, s), 55.6 (1C, s), 15.0 (1C, s), 14.5 (1C, s); Calcd./Anal., for C_17_H_16_ClN_3_O_2_S: C, 56.43; H, 4.46; N, 11.61. Found: C, 56.68; H, 4.27; N, 11.66.
**4c:** *Ethyl 4-(1,3-diphenyl-1H-pyrazol-4-yl)-6-methyl-2-thioxo-1,2,3,4-tetrahydropyrimidine-5-carboxylate:* Yield: 75%; m.p.: 218–220 °C; ^1^HNMR (DMSO-*d*_6_, 400 MHz) *δ*: 2.23 (t, 3H, CH_3_), 2.50 (s, 3H, CH_3_), 3.92 (q, 2H, CH_3_CH_2_-O), 5.4 (s, 1H, CH), 7.16–8.11 (m, 11H, aromatic), 10.31, 11.49 (2 s, 2H, 2NH (D_2_O exchangeable)). ^13^C NMR: (DMSO-*d*_6_, 400 MHz) *δ* ppm) 169.7 (1C, s), 162.0 (1C, s), 154.3 (1C, s), 146.1 (1C, s), 137.6 (1C, s), 130.6 (1C, s), 129.8 (2C, s), 128.2 (2C, s), 127.8-127.8 (2C, 127.8 (s), 127.8 (s)), 127.5 (1C, s), 127.3–127.8 (3C, 127.3 (s), 127.8 (s)), 121.8 (2C, s), 113.4 (1C, s), 60.2 (1C, s), 55.0 (1C, s), 15.1 (1C, s), 14.6 (1C, s), 14.2 (1C, s); Calcd./Anal., for C_17_H_16_ClN_3_O_2_S: C, 56.43; H, 4.46; N, 11.61. Found: C, 56.68; H, 4.27; N, 11.66.

A solution of any of **4a**–**c** (0.01 mol) in 50 mL of 10% alcoholic NaOH was refluxed for 2 h. The mixture was then cooled and acidified with conc. HCl, the precipitate was filtered off, washed with water, dried under suction and recrystallized from ethanol.

**5a:** *4-(Indol-3yl)-6-methyl-2-thioxo-1,2,3,4-tetrahydropyrimidine-5-carboxylic acid:* Yield: 69%; m.p.: 233–235 °C; ^1^HNMR (DMSO-*d*_6_, 400 MHz) *δ*: 2.23 (t, 3H, CH_3_), 5.53 (s, 1H, CH), 7.31–8.05 (m, 5H, aromatic), 10.32, 10.87, 11.19 (3 s, 3H, 3NH (D2O exchangeable)). 13C NMR: (DMSO-*d*_6_, 400 MHz) *δ* (ppm) 177.5 (1C, s), 174.7 (1C, s), 154.9 (1C, s), 148.9 (1C, s), 128.1–128.3 (2C, 128.2 (s), 128.2 (s)), 128.4 (1C, s), 126.1 (1C, s), 125.8 (1C, s), 125.5 (1C, s), 121.7 (1C, s), 113.4 (1C, s), 51.9 (1C, s), 16.9 (1C, s); Calcd./Anal., for C_14_H_13_N_3_O_3_S: C, 55.43; H, 4.32; N, 13.85. Found: C, 55.66; H, 4.25; N, 13.55.
**5b:** *4-(2-Chloroquinolin-3-yl)-6-methyl-2-thioxo-1,2,3,4-tetrahydropyrimidine-5-carboxylic acid:* Yield: 71%; m.p.: 244–246 °C; ^1^HNMR (DMSO-*d*_6_, 400 MHz) *δ*: 2.44 (t, 3H, CH_3_), 5.47 (s, 1H, CH), 7.26–8.05 (m, 5H, aromatic), 10.35, 11.34 (2 s, 2H, 2NH (D_2_O exchangeable)). ^13^C NMR: (DMSO-*d*_6_, 400 MHz) *δ* (ppm) 172.3 (1C, s), 165.3 (1C, s), 152.6 (1C, s), 146.0 (1C, s), 141.1 (1C, s), 130.6 (1C, s), 128.5 (1C, s), 128.3 (1C, s), 127.4 (1C, s), 126.0 (1C, s), 125.5 (1C, s), 121.8 (1C, s), 119.9 (1C, s), 55.8 (1C, s), 18.0 (1C, s); Calcd./Anal., for C_15_H_12_ClN_3_O_3_S: C, 51.51; H, 3.46; N, 12.01. Found: C, 51.60; H, 3.35; N, 12.25.
**5c:** *4-(1,3-Diphenyl-1H-pyrazol-4-yl)-6-methyl-2-thioxo-1,2,3,4-tetrahydropyrimidine-5-carboxylic acid:* Yield: 68%; m.p.: 214–216 °C; ^1^HNMR (DMSO-*d*_6_, 400 MHz) *δ*: 2.50 (t, 3H, CH_3_), 5.39 (s, 1H, CH), 7.1–7.91 (m, 11H, aromatic), 10.31, 11.39 (2 s, 2H, 2NH (D_2_O exchangeable)). ^13^C NMR: (DMSO-*d*_6_, 400 MHz) *δ* (ppm) 169.2 (1C, s), 164.1 (1C, s), 154.3 (1C, s), 146.7 (1C, s), 138.1 (1C, s), 131.6 (1C, s), 130.0 (2C, s), 128.8 (2C, s), 126.0–127.5 (2C, 127.6 (s), 128.3 (s)), 125.5 (1C, s), 121.9 (2C, s), 114.6 (1C, s), 55.1 (1C, s), 16.8 (1C, s); Calcd./Anal., for C_21_H_18_N_4_O_3_S: C, 62.05; H, 4.46; N, 13.78. Found: C, 62.30; H, 4.45; N, 13.55.

A mixture of any of **5a**–**c** (0.01 mol) and 15 mL thionyl chloride was refluxed for 40 min. Unreacted thionyl chloride was removed by heating the reaction mixture on a water bath. The produced acid chlorides **6a**–**c** were rapidly dried under suction and used as crude for subsequent work.

**6a:** *4-(1H-indol-3-yl)-6-methyl-2-thioxo-1,2,3,4-tetrahydropyrimidine-5-carbonyl chloride:* Yield: 68%; m.p.: 247–249 °C; ^1^HNMR (DMSO-*d*_6_, 400 MHz) *δ*: 2.23 (t, 3H, CH_3_), 5.53 (s, 1H, CH), 7.41–8.07 (m, 5H, aromatic), 10.39, 10.67, 10.84 (3 s, 3H, 3NH (D2O exchangeable)). 13C NMR: (DMSO-*d*_6_, 400 MHz) *δ* (ppm) 178.3 (1C, s), 173.4 (1C, s), 153.9, 149.0 (1C, s), 142.1(1C, s), 135.3 (1C, s), 129.3–128.3, (2C, 129.3 (s), 126.3 (s)) 127.4 (1C, s), 125.7 (1C, s), 122.0 (1C, s), 113.5 (1C, s), 110.9 (1C, s), 52.3 (1C, s), 17.8 (1C, s); Calcd./Anal., for C_15_H_14_ClN_3_OS: C, 56.33; H, 4.41; N, 13.14. Found: C, 56.28; H, 4.48; N, 13.22.
**6b:** *4-(2-Chloroquinolin-3-yl)-6-methyl-2-thioxo-1,2,3,4-tetrahydropyrimidine-5-carbonyl chloride:* Yield: 73%; m.p.: 254–256 °C; ^1^HNMR (DMSO-*d*_6_, 400 MHz) *δ*: 2.34 (t, 3H, CH_3_), 5.37 (s, 1H, CH), 7.26–8.05 (m, 5H, aromatic), 10.35, 10.94 (2 s, 2H, 2NH (D_2_O exchangeable)). ^13^C NMR: (DMSO-*d*_6_, 400 MHz) *δ* (ppm) 178.1 (1C, s), 171.6 (1C, s), 152.7 (1C, s), 147.6 (1C, s), 146.1 (1C, s), 141.0 (1C, s), 130.6 (1C, s), 128.5 (1C, s), 128.2 (1C, s), 127.4 (1C, s), 126.0 (1C, s), 125.8 (1C, s), 121.8 (1C, s), 55.4 (1C, s), 16.8 (1C, s); Calcd./Anal., for C_16_H_13_Cl_2_N_3_OS: C, 52.47; H, 3.58; N, 11.47. Found: C, 52.29; H, 3.66; N, 11.25.
**6c:** *4-(Diphenyl-1H-pyrazol-4-yl)-6-methyl-2-thioxo-1,2,3,4-tetrahydropyrimidine-5-carbonyl chloride:* Yield: 80%; m.p.: 235–237 °C; ^1^HNMR (DMSO-*d*_6_, 400 MHz) *δ*: 2.34 (t, 3H, CH_3_), 5.32 (s, 1H, CH), 7.15–8.23 (m, 11H, aromatic), 10.41, 11.43 (2 s, 2H, 2NH (D_2_O exchangeable)). ^13^C NMR: (DMSO-*d*_6_, 400 MHz) *δ* (ppm) 174.3 (1C, s), 167.0 (1C, s), 155.9 (1C, s), 148.0 (1C, s), 138.3 (1C, s), 130.6 (1C, s), 129.98 (2C, s), 128.8 (2C, s), 128.1–127.5 (1C, s), 127.5–121.8 (4C, 121.8 (s), 125.7 (s), 127.5 (s)), 112.8 (2C, s), 55.3 (1C, s), 17.9 (1C, s); Calcd./Anal., for C_22_H_19_ClN_4_OS: C, 62.48; H, 4.53; N, 13.25. Found: C, 62.21; H, 4.32; N, 13.25.

A mixture of any **6a**–**c** (0.01 mol) and the appropriate aromatic amine (0.01 mol) in 25 mL ethanol was refluxed for 5hrs, then cooled, filtered off, dried and recrystallized from ethanol.

**7a:** *4-(1H-indoly-3-yl)-6-methyl-N-phenyl-2-thioxo-1,2,3,4-tetrahydropyrimidine-5-carboxamide:* Yield: 77%; m.p.: 266–268 °C; ^1^HNMR (DMSO-*d*_6_, 400 MHz) *δ*: 2.36 (t, 3H, CH_3_), 5.19 (s, 1H, CH), 7.11–9.59 (m, 11H, aromatic), 10.65, 11.03, 11.37 (3 s, 3H, 3NH (D_2_O exchangeable)). ^13^C NMR: (DMSO-*d*_6_, 400 MHz) *δ* (ppm) 167.6 (1C, s), 165.1 (1C, s), 153.9, 146.7 (1C, s), 139.1 (1C, s), 135.3 (1C, s), 130.5 (1C, s), 129.8-128.8 (4C, 128.8 (s), 129.0 (s), 129.8 (s)), 128.3 (1C, s), 127.5 (1C, s), 127.5 (1C, s), 125.4 (2C, s), 121.8 (1C, s), 113.4 (1C, s), 53.9 (1C, s), 17.3 (1C, s); Calcd./Anal., for C_20_H_18_N_4_OS: C, 66.28; H, 5.01; N, 15.46. Found: C, 66.45; H, 4.99; N, 15.35.
**7b:** *4-(2-Chloroquinolin-3-yl)-6-methyl-N-phenyl-2-thioxo-1,2,3,4-tetrahydropyrimidine-5-carboxamide:* Yield: 78%; m.p.: 260–262 °C; ^1^HNMR (DMSO-*d*_6_, 400 MHz) *δ*: 2.24 (t, 3H, CH_3_), 5.3 (s, 1H, CH), 7.29–8.21 (m, 10H, aromatic), 9.89, 10.35, 11.39 (3 s, 3H, 3NH (D_2_O exchangeable)). ^13^C NMR: (DMSO-*d*_6_, 400 MHz) *δ* (ppm) 177.9 (1C, s), 163.7 (1C, s), 155.9 (1C, s), 148.9 (1C, s), 145.1 (1C, s), 141.2 (1C, s), 139.7 (1C, s), 134.5 (1C, s), 130.6 (1C, s), 128.8 (2C, s), 128.6 (1C, s), 128.3–127.4 (2C, 128.3 (s), 127.4 (s)), 126.3 (1C, s), 125.8, 125.6 (1C, s), 121.7 (1C, s), 114.5 (2C, s), 57.1 (1C, s), 17.3 (1C, s); MS (EI) *m*/*z*: 408.11 (M+, 20.5%); Calcd./Anal., for C_21_H_17_ClN_4_OS: C, 61.68; H, 4.19; N, 13.70. Found: C, 61.69; H, 4.20; N, 13.46.
**7c:** *4-(1,3-Diphenyl-1H-pyrazol-4-yl)-6-methyl-N-phenyl-2-thioxo-1,2,3,4-tetrahydropyrimidine-5-carboxamide:* Yield: 73%; m.p.: 222–224 °C; ^1^HNMR (DMSO-*d*_6_, 400 MHz) *δ*: 2.24 (t, 3H, CH_3_), 5.42 (s, 1H, CH), 7.25–8.18 (m, 10H, aromatic), 10.41, 10.73, 11.71 (3 s, 3H, 3NH (D_2_O exchangeable)). ^13^C NMR: (DMSO-*d*_6_, 400 MHz) *δ* (ppm) 177.2 (1C, s), 161.62 (1C, s), 154.98 (1C, s), 148.93 (1C, s), 146.06 (1C, s), 141.07 (1C, s), 139.67 (1C, s), 134.53 (2C, s), 130.6–128.8 (4C, 130.61 (s), 128.8 (s)), 128.5–127.4 (3C, 128.5 (s), 128.3 (s), 127.4 (s)), 126.3 (1C, s), 126.0–125.5 (4C, 126.0 (s), 125.8 (s), 125.5 (s)), 121.75 (2C, s), 114.41(2C, s), 57.53 (1C, s), 17.38 (1C, s); Calcd./Anal., for C_27_H_23_N_5_OS: C, 69.65; H, 4.98; N, 15.04. Found: C, 69.59; H, 4.90; N, 14.98.
**8a:** *4-(1H-indol-3-yl)-6-methyl-thioxo-N-p-tolyl-1,2,3,4-tetrahydropyrimidine-5-carboxamide:* Yield: 76%; m.p.: 262–264 °C; ^1^HNMR (DMSO-*d*_6_, 400 MHz) *δ*: 2.14 (t, 3H, CH_3_), 2.29 (t, 3H, CH_3_), 5.51 (s, 1H, CH), 7.31–8.19 (m, 9H, aromatic), 9.68, 10.36, 10.83, 11.35 (4 s, 4H, 4NH (D_2_O exchangeable)). ^13^C NMR: (DMSO-*d*_6_, 400 MHz) *δ* (ppm) 167.6 (1C, s), 163.8 (1C, s), 153.9 (1C, s), 146.7 (1C, s), 140.9 (1C, s), 135.3 (1C, s), 130.5 (2C, s), 129.8 (1C, s), 129–128.2 (4C, 129.2 (s), 128.2 (s)), 127.5 (1C, s), 127.5 (1C, s), 126.0 (1C, s), 125.4 (1C, s), 121.8 (2C, s), 113.4 (1C, s), 53.9 (1C, s), 20.5 (1C, s), 17.6 (1C, s); Calcd./Anal., for C_21_H_20_N_4_OS: C, 67.00; H, 5.35; N, 14.88. Found: C, 67.36; H, 5.01; N, 14.84.
**8b:** *4-(2-Chloroquinolin-3-yl)-6-methyl-thioxo-N-p-tolyl-1,2,3,4-tetrahydropyrimidine-5-carboxamide:* Yield: 77%; m.p.: 282–284 °C; ^1^HNMR (DMSO-*d*_6_, 400 MHz) *δ*: 2.13 (t, 3H, CH_3_), 2.25 (t, 3H, CH_3_), 5.47 (s, 1H, CH), 7.23–8.23 (m, 9H, aromatic), 10.15, 10.45, 11.5 (3 s, 3H, 3NH (D_2_O exchangeable)). ^13^C NMR: (DMSO-*d*_6_, 400 MHz) *δ* (ppm) 172.3 (1C, s), 167.5 (1C, s), 152.6 (1C, s), 148.9 (1C, s), 144.2 (1C, s), 139.7 (1C, s), 130.6 (1C, s) (1C, s), 128.8 (2C, s), 128.6 (1C, s), 128.3 (1C, s), 127.4 (1C, s), 126.7 (1C, s), 126.3 (1C, s), 125.8 (1C, s), 121.7 (1C, s), 114.5 (2C, s), 57.4 (1C, s), 20.4 (1C, s), 17.3 (1C, s); Calcd./Anal., for C_22_H_19_ClN_4_OS: C, 62.48; H, 4.53; N, 13.25. Found: C, 62.55; H, 4.31; N, 13.36.
**8c:** *4-(1,3-Diphenyl-1H-pyrazol-4-yl)-6-methyl-2-thioxo-N-p-tolyl-1,2,3,4-tetrahydropyrimidine-5-carboxamide:* Yield: 77%; m.p.: 275–277 °C; ^1^HNMR (DMSO-*d*_6_, 400 MHz) *δ*: 2.12 (t, 3H, CH_3_), 2.34 (t, 3H, CH_3_), 5.42 (s, 1H, CH), 7.15–8.19 (m, 14H, aromatic), 9.82, 10.7, 11.3 (3 s, 3H, 3NH (D_2_O exchangeable)). ^13^C NMR: (DMSO-*d*_6_, 400 MHz) *δ* (ppm) 178.7 (1C, s), 168.1 (1C, s), 155.1 (1C, s), 151.0 (1C, s), 143.7 (1C, s), 142.0 (1C, s), 141.2 (1C, s), 139.7 (1C, s), 134.5 (2C, s), 130.6 (2C, s), 128.8 (2C, s), 128.6–128.3 (4C, 128.6 (s), 128.5 (s), 128.3 (s)), 127.5 (1C, s), 126.2–125.5 (4C, 126.2 (s), 126.2 (s), 125.5 (s)), 121.8 (2C, s), 113.1 (2C, s), 57.9 (1C, s), 23.0 (1C, s), 17.4 (1C, s); Calcd./Anal., for C_28_H_25_N_5_OS: C, 70.12; H, 5.25; N, 14.60. Found: C, 70.35; H, 5.11; N, 14.50.
**9a:** *4-(1H-indol-3-yl)-6-methyl-N-(4-nitrophenyl)-2-thioxo-1,2,3,4-tetrahydropyrimidine-5-carboxamide:* Yield: 83%; m.p.: 263–265 °C; ^1^HNMR (DMSO-*d*_6_, 400 MHz) *δ*: 2.29 (t, 3H, CH_3_), 5.5 (s, 1H, CH), 7.31–8.09 (m, 9H, aromatic), 9.89, 10.35, 10.8, 11.29 (3 s, 3H, 3NH (D_2_O exchangeable)). ^13^C NMR: (DMSO-*d*_6_, 400 MHz) *δ* (ppm) 168.2 (1C, s), 164.0 (1C, s), 153.1 (1C, s), 148.5 (1C, s), 146.9 (1C, s), 139.3 (1C, s), 135.2 (1C, s), 129.2-128.3 (2C, 128.3 (s), 129.2 (s)), 128.4 (1C, s), 127.6 (1C, s), 127.5 (2C, s), 126.6 (1C, s), 125.4 (1C, s), 121.7 (2C, s), 114.0 (1C, s), 54.7 (1C, s), 17.6 (1C, s); Calcd./Anal., for C_20_H_17_N_5_O_3_S: C, 58.96; H, 4.21; N, 17.19. Found: C, 58.70; H, 4.06; N, 17.21.
**9b:** *4-(2-Chloroquinolin-3-yl)-6-methyl-N-(4-nitrophenyl)-2-thioxo-1,2,3,4-tetrahydropyrimidine-5-carboxamide:* Yield: 83%; m.p.: 276–278 °C; ^1^HNMR (DMSO-*d*_6_, 400 MHz) *δ*: 2.13 (t, 3H, CH_3_), 5.45 (s, 1H, CH), 7.17–8.19 (m, 9H, aromatic), 9.95, 10.86, 11.3 (3 s, 3H, 3NH (D_2_O exchangeable)). ^13^C NMR: (DMSO-*d*_6_, 400 MHz) *δ* (ppm) 174.4 (1C, s), 168.3 (1C, s), 156.1 (1C, s), 149.0 (1C, s), 146.5 (1C, s), 143.6 (1C, s), 135.2 (1C, s), 130.7 (1C, s), 128.8 (1C, s), 128.5 (1C, s), 128.2 (1C, s), 127.4 (1C, s), 126.6 (1C, s), 126.3 (1C, s), 125.6 (2C, s), 121.7 (1C, s), 115.0 (2C, s), 57.5 (1C, s), 17.4 (1C, s); Calcd./Anal., for C_21_H_16_ClN_5_O_3_S: C, 55.57; H, 3.55; N, 15.43. Found: C, 55.67; H, 3.88; N, 15.26.
**9c:** *4-(1,3-Diphenyl-1H-pyrazol-4-yl)-6-methyl-N-(4-nitrophenyl)-2-thioxo-1,2,3,4-tetrahydropyrimidine-5-carboxamide:* Yield: 83%; m.p.: 261–263 °C; ^1^HNMR (DMSO-*d*_6_, 400 MHz) *δ*: 2.4 (t, 3H, CH_3_), 5.59 (s, 1H, CH), 7.25–8.19 (m, 14H, aromatic), 9.58, 10.5, 11.59 (3 s, 3H, 3NH (D_2_O exchangeable)). ^13^C NMR: (DMSO-*d*_6_, 400 MHz) *δ* (ppm) 178.7 (1C, s), 168.5 (1C, s), 158.9 (1C, s), 155.1 (1C, s), 151.6, (1C, s) 143.9 (1C, s), 142.0 (1C, s), 141.1 (1C, s), 130.7 (2C, s), 128.8 (2C, s), 128.6–128.3 (2C, 128.86 (s), 128.3 (s)), 127.4 (1C, s), 126.2, 125.6 (4C, 126.2 (s), 126.8 (s), 125.6 (s)), 125.5 (2C, s), 121.1 (2C, s), 114.2 (2C, s), 57.8 (1C, s), 17.5 (1C, s); Calcd./Anal., for C_27_H_22_N_6_O_3_S: C, 63.52; H, 4.34; N, 16.46. Found: C, 63.87; H, 4.35; N, 16.66.

The respective spectra for all the above compounds and techniques are presented as Supplementary Information.

### 3.2. Biological Evaluation

#### 3.2.1. Evaluation of In Vivo Antihypertensive Activity

Rats of either sex were administered heparin at the dose of 2000 IU/Kg by IV route. Rats were anaesthetized with Pentothal sodium 80 mg/Kg given intraperitoneally. The blood pressure transducer was initially calibrated using a mercury manometer. For each rat, the carotid artery was cannulated and attached to a blood pressure transducer to record the initial arterial blood pressure which will be calibrated initially via the mercury manometer. In a similar way on the opposite side, the jugular vein was cannulated to administer 0.3 mL heparinized saline for checking the effect of a normal flow of fluid in the vein on blood pressure by inhibition of the adrenaline response.

#### 3.2.2. Evaluation of Calcium Channel Blocking Activity

Contraction of ileum was induced by adding potassium chloride and calcium chloride to the organ bath containing slightly modified Tyrode solution (NaCl = 8.0gm/L, KCl = 0.2 g/L, CaCl_2_ = 0.18 g/L, NaH_2_PO_4_ = 0.1 g/L, MgCl_2_ = 0.1g/L, Glucose = 1.0 g/L, NaHCO_3_ = 1.0g/L). Test drugs with calcium channel blocking activity have a relaxing effect. Test drugs of a concentration of 2 mg/mL (0.3 mL in volume) were used for the study.

The assembly was set up and arrangements were made for the experiment. The animal was kept for overnight fasting, then stunned by a sharp blow on head and sacrificed by cutting the blood vessels in the neck. The abdominal cavity was quickly opened, and a piece of ileum was isolated. It was placed in a Petri dish containing tyrode solution maintained at 37 °C. The mesentery of ileum was removed, and the interior content was washed by blowing Tyrode solution (NaCl = 8.0 g/L, KCl = 0.2 g/L, CaCl_2_ = 0.18 g/L, NaH_2_PO_4_ = 1 g/L, MgCl_2_ = 0.1 g/L, Glucose = 1.0 g/L, NaHCO_3_ = 1.0 g/L) with help of a pipette. The tissue was mounted in a mammalian organ bath and connected to isotonic frontal writing lever. The tissue was allowed to stabilize for 30 min. The responses of acetylcholine were taken till the maximum effect was obtained. The normal tyrode solution was changed with tyrode containing the test solution. The responses of acetylcholine were taken with the same dose and continued till maximum effect obtained. The percentage of relaxation from the test-drug pre-contracted level was calculated for each concentration of test compound. An IC50 was calculated by linear regression analysis:

y = 96.18x + 1.372

If y = 50%, then x = 0.5 mL dose.

$${IC}_{50}\;of\;Nifedipine=dose\;for\;50\%\;inhibition\times \frac{Concentration}{bath\;capacity}\;=0.5\times \frac{1000}{25}=20\;\mu g/mL$$

### 3.3. Molecular Modeling and Binding Mode Prediction

Based on the pharmacological results, we selected compounds **8c** and **9c** and RyR1, along with dihydropyridine inhibitors, for use as the docking model (PDB ID: 6M7H, and 4MS2) [35,36].

Computer-guided docking experiments were carried out using Molecular Operating Environment (MOE 2015.10) software (Version 10), Chemical Computing Group, Montreal, Canada. Molecular docking studies were examined to gain deeper insight into the molecular bases of the inhibitory potency and for the purpose of lead optimization and to pick up the interaction between compounds and the Ryanodine receptor.

## 4. Conclusions

The present study aimed to synthesize novel dihydropyrimidine analogues having active constituents at C5 position using three different aldehydes depending on the electron affinity as well as the polarity of the functionalities. The employed Biginelli reaction with considered aldehydes and aromatic amines yielded several DHPM analogues. The evaluation of antihypertensive and CCB activity demonstrated better activity in compounds **4a, 7a, 7c, 8c, 9b** and **9c.** The results were on par with activities of Nifedipine. Compounds **8c** and **9c** presented better IC_50_ values, which further lead to the molecular docking investigations. The binding affinities of the synthesized compounds with considered receptors was attributed to the hydrogen donor and acceptor groups, pi-electron clouds in the docked systems. The work concludes that, the results compared with the standard drug and the literature suggested the enhanced activity for the heterocyclic substitution at C_4_ position of DHPM. Perhaps the symmetry or asymmetry established at other positions and the substitution of several other aromatic and heterocyclic systems should be explored in detail to substantiate the conclusions. The experimental and computational predictions of these compounds provide the productive matrix for further progress of potent and deserving CCBs.

## Figures and Tables

**Table 1:** Screening of antihypertensive activity by measuring % inhibition in blood pressure.

Compound Code	Dose (mL)	Control (mmHg)	Test (mmHg)	% Inhibition in Blood Pressure
**Nifedipine**	0.3	29.07	20.02	31.32
	0.3	28.35	20.78	26.54
**4a**	0.3	28.41	23.09	19.78
	0.3	27.57	22.38	19.19
**4b**	0.3	28.34	23.5	18.53
	0.3	28.38	24.18	18.79
**4c**	0.3	29.32	22.17	29.40
	0.3	30.15	23.45	29.78
**5a**	0.3	29.27	25.07	15.82
	0.3	29.13	25.63	15.12
**5b**	0.3	30.01	24.21	15.23
	0.3	29.23	24.14	15.55
**5c**	0.3	28.19	23.43	16.32
	0.3	28.32	23.54	16.09
**6a**	0.3	30.23	26.54	11.75
	0.3	30.02	26.23	11.79
**6b**	0.3	28.90	26.85	11.09
	0.3	28.38	26.92	11.07
**6c**	0.3	30.12	27.45	11.00
	0.3	30.01	27.09	11.02
**7a**	0.3	29.34	20.19	31.42
	0.3	30.18	21.42	27.58
**7b**	0.3	29.19	21.98	23.23
	0.3	30.01	21.86	23.90
**7c**	0.3	29.33	22.50	27.32
	0.3	28.45	22.21	28.41
**8a**	0.3	30.13	24.42	20.44
	0.3	29.62	23.53	21.02
**8b**	0.3	30.51	25.61	18.21
	0.3	30.00	25.03	19.81
**8c**	0.3	29.14	22.62	28.70
	0.3	29.48	20.07	30.26
**9a**	0.3	29.15	23.15	24.30
	0.3	30.17	22.54	26.86
**9b**	0.3	29.32	20.19	30.09
	0.3	30.15	21.42	29.28
**9c**	0.3	29.71	21.42	27.44
	0.3	30.43	20.53	31.02

**Table 2:** Screening of calcium channel blocking activity.

Compound Code	Dose (mL)	Control (cm)	Test (cm)	% Inhibition	IC_50_ (μg/L)
**Nifedipine**	0.1	3.4	3.0	11.74	21 ± 0.28
	0.2	3.4	2.7	20.47	
	0.3	3.4	2.3	32.26	
	0.4	3.3	2.1	35.76	
	0.5	3.3	1.7	48.38	
	0.6	3.3	1.2	61.56	
**4a**	0.1	3.4	2.9	17.24	23.07 ± 0.32
	0.3	3.4	2.3	20.56	
	0.5	3.3	1.8	30.80	
**4b**	0.1	3.4	2.8	16.78	24.69 ± 0.22
	0.3	3.4	2.3	19.36	
	0.5	3.3	1.6	29.83	
**4c**	0.1	3.3	2.4	16.25	22.42 ± 0.13
	0.3	3.4	2.5	25.69	
	0.5	3.4	1.3	37.27	
**5a**	0.1	3.4	2.6	14.88	24.37 ± 0.41
	0.3	3.3	2.1	19.71	
	0.5	3.4	1.6	35.23	
**5b**	0.1	3.3	2.7	14.87	25.25 ± 0.38
	0.3	3.4	2.3	19.98	
	0.5	3.4	1.8	34.89	
**5c**	0.1	3.4	2.7	13.67	24.81 ± 0.43
	0.3	3.4	2.3	18.96	
	0.5	3.3	1.5	32.96	
**6a**	0.1	3.3	2.6	8.56	26.84 ± 0.26
	0.3	3.4	2.3	17.87	
	0.5	3.4	1.8	30.84	
**6b**	0.1	3.4	2.7	7.98	30.78 ± 0.24
	0.3	3.3	2.1	16.98	
	0.5	3.4	1.8	30.09	
**6c**	0.1	3.3	2.7	8.98	29.73 ± 0.19
	0.3	3.4	2.1	17.43	
	0.5	3.4	1.5	30.53	
**7a**	0.1	3.4	2.8	16.64	21.57 ± 0.12
	0.3	3.4	2.2	28.59	
	0.5	3.4	1.7	39.10	
**7b**	0.1	3.4	2.6	15.09	22.11 ± 0.20
	0.3	3.4	2.2	30.93	
	0.5	3.3	1.8	36.89	
**7c**	0.1	3.4	2.7	17.96	20.05 ± 0.05
	0.3	3.4	2.2	29.97	
	0.5	3.3	1.5	44.98	
**8a**	0.1	3.3	2.7	16.76	22.63 ± 0.36
	0.3	3.4	2.1	28.98	
	0.5	3.4	1.5	45.83	
**8b**	0.1	3.3	2.7	16.76	22.91 ± 0.29
	0.3	3.4	2.1	29.56	
	0.5	3.4	1.5	44.87	
**8c**	0.1	3.4	2.7	16.24	19.83 ± 0.36
	0.3	3.4	2.1	35.23	
	0.5	3.4	1.5	50.13	
**9a**	0.1	3.4	2.8	15.81	21.33 ± 0.21
	0.3	3.4	2.1	34.53	
	0.5	3.3	1.3	45.82	
**9b**	0.1	3.4	2.6	17.12	20.45 ± 1.07
	0.3	3.4	2.01	37.45	
	0.5	3.3	1.4	49.75	
**9c**	0.1	3.3	2.5	17.76	19.57 ± 0.18
	0.3	3.4	2.1	36.20	
	0.5	3.4	1.6	50.86	

**Table 3:** Summary of Molecular Operating Environment (MOE) docking results for the synthesized compound with target proteins.

Ligands	Hydrogen Bonds between Atoms of Ligands and Amino Acids of Receptor						S-Score (Binding Energy) (kcal/mol)
	Ligands Atoms	Receptor		Type	Distance (Å)	Energy (kcal/mol)	
		Atoms	Residues				
Ryanodine receptor							
KN93	C11	OE1	GLU 11	H-donor	3.15	−0.7	−7.11
8c	O11	SD	MET 72	H-donor	3.47	−0.5	−7.04
9c	C20	6-ring	PHE 92	H-pi	4.47	−0.7	−8.41
Nifedipine	N	O	GLU 11	H-donor	2.71	−0.91	−5.28
dihydropyridine receptor							
PX4	O2	NE1	TRP 1076	H-acceptor	3.43	−0.9	−5.91
8c	5-ring	NE1	TRP 1076	pi-H	4.22	−1.0	−5.09
9c	S21	CA	SER 1125	H-acceptor	4.13	−0.8	−6.89
Nifedipine	CO	N	GLU 1096	H-acceptor	2.50	−0.9	−5.74
	CO	N	TYR 1193	H-acceptor	2.36		

## Data Availability

Not applicable.

## Supplementary Materials

The following supporting information can be downloaded at: https://www.mdpi.com/article/10.3390/molecules28124869/s1, Supplementary data includes detailed synthesis and characterization of all compounds, molecular modeling and binding mode evaluation to representative inhibitors as well as their spectral analyses data.

## Sample Availability

Samples of the compounds are not available from the authors upon request.

## References

[1] Di Palo K.E., Barone N.J. (2020). Hypertension and heart failure: Prevention, targets, and treatment. Heart Fail. Clin..

[2] Luyckx V.A., Bertram J.F., Brenner B.M., Fall C., Hoy W.E., Ozanne S.E., Vikse B.E. (2013). Effect of fetal and child health on kidney development and long-term risk of hypertension and kidney disease. Lancet.

[3] Ram C.V.S. (2010). Beta-blockers in hypertension. Am. J. Cardiol..

[4] Matchar D.B., McCrory D.C., Orlando L.A., Patel M.R., Patel U.D., Patwardhan M.B., Powers B., Samsa G.P., Gray R.N. (2008). Systematic review: Comparative effectiveness of angiotensin-converting enzyme inhibitors and angiotensin II receptor blockers for treating essential hypertension. Ann. Intern. Med..

[5] Cutler J.A., Davis B.R. (2008). Thiazide-type diuretics and β-adrenergic blockers as first-line drug treatments for hypertension. Circulation.

[6] Zhu J., Chen N., Zhou M., Guo J., Zhu C., Zhou J., Ma M., He L. (2021). Calcium channel blockers versus other classes of drugs for hypertension. Cochrane Database Syst. Rev..

[7] Rothwell P.M., Howard S.C., Dolan E., O’Brien E., Dobson J.E., Dahlöf B., Poulter N.R., Sever P.S. (2010). Effects of β blockers and calcium-channel blockers on within-individual variability in blood pressure and risk of stroke. Lancet Neurol..

[8] Özkaya E., Yazganoğlu K.D., Özkaya E., Yazganoğlu K.D. (2014). Calcium Channel Blockers. Advers. Cutan. Drug React. Cardiovasc. Drugs.

[9] Chatki P.K., Tabassum S. (2021). Analytical Methods of Dihydropyridines Based Calcium Channel Blockers-Amlodipine, Lacidipine, Isradipine, Nifedipine, Felodipine, Cilnidipine and its related formulations: A Review. Asian J. Res. Chem..

[10] Loas G., Van de Borne P., Darquennes G., Le Corre P. (2022). Association of amlodipine with the risk of in-hospital death in patients with COVID-19 and hypertension: A reanalysis on 184 COVID-19 patients with hypertension. Pharmaceuticals.

[11] Wang A.L., Iadecola C., Wang G. (2017). New generations of dihydropyridines for treatment of hypertension. J. Geriatr. Cardiol. JGC.

[12] Liang L., Kung J.Y., Mitchelmore B., Cave A., Banh H.L. (2022). Comparative peripheral edema for dihydropyridines calcium channel blockers treatment: A systematic review and network meta-analysis. J. Clin. Hypertens..

[13] van Zwieten P.A., Pfaffendorf M. (1993). Pharmacology of the dihydropyridine calcium antagonists: Relationship between lipophilicity and pharmacodynamic responses. J. Hypertens. Suppl. Off. J. Int. Soc. Hypertens..

[14] Farghaly A.M., AboulWafa O.M., Elshaier Y.A., Badawi W.A., Haridy H.H., Mubarak H.A. (2019). Design, synthesis, and antihypertensive activity of new pyrimidine derivatives endowing new pharmacophores. Med. Chem. Res..

[15] Alam O., Khan S.A., Siddiqui N., Ahsan W., Verma S.P., Gilani S.J. (2010). Antihypertensive activity of newer 1, 4-dihydro-5-pyrimidine carboxamides: Synthesis and pharmacological evaluation. Eur. J. Med. Chem..

[16] Marinescu M. (2021). Biginelli reaction mediated synthesis of antimicrobial pyrimidine derivatives and their therapeutic properties. Molecules.

[17] Kappe C.O. (1998). 4-Aryldihydropyrimidines via the Biginelli condensation: Aza-analogs of nifedipine-type calcium channel modulators. Molecules.

[18] Rozzo M. (2014). Development of a Green Method for the Production of Dihydropyrimidinones Through a Biginelli Reaction. https://digitalcommons.butler.edu/urc/2014/chemistry/1/.

[19] Drapak I., Perekhoda L., Tsapko T., Berezniakova N., Tsapko Y. (2017). Cardiovascular calcium channel blockers: Historical overview, development and new approaches in design. J. Heterocycl. Chem..

[20] Zohny Y.M., Awad S.M., Rabie M.A., Al-Saidan O.A. (2023). Synthesis of Dihydropyrimidines: Isosteres of Nifedipine and Evaluation of Their Calcium Channel Blocking Efficiency. Molecules.

[21] Şafak C., Gündüz M.G., İlhan S.Ö., Şimşek R., İşli F., Yıldırım Ş., Fincan G.S.Ö., Sarıoğlu Y., Linden A. (2012). Synthesis and Myorelaxant Activity of Fused 1,4-Dihydropyridines on Isolated Rabbit Gastric Fundus. Drug Dev. Res..

[22] Tu S., Miao C., Fang F., Youjian F., Li T., Zhuang Q., Zhang X., Zhu S., Shi D. (2004). New potential calcium channel modulators: Design and synthesis of compounds containing two pyridine, pyrimidine, pyridone, quinoline and acridine units under microwave irradiation. Bioorg. Med. Chem. Lett..

[23] Lipkind G.M., Fozzard H.A. (2003). Molecular modeling of interactions of dihydropyridines and phenylalkylamines with the inner pore of the L-type Ca^2+^ channel. Mol. Pharmacol..

[24] El-Khouly A., Gündüz M., Cengelli C., Şimşek R., Erol K., Şafak C., Yıldırım S.Ö., Butcher R. (2013). Microwave-assisted synthesis and spasmolytic activity of 4-indolylhexahydroquinoline derivatives. Drug Res..

[25] Bisi A., Budriesi R., Rampa A., Fabbri G., Chiarini A., Valenti P. (1996). Synthesis and pharmacological profile of some chloroxanthone-1, 4-dihydropyridine derivatives. Arzneimittelforschung.

[26] Miri R., Javidnia K., Sarkarzadeh H., Hemmateenejad B. (2006). Synthesis, study of 3D structures, and pharmacological activities of lipophilic nitroimidazolyl-1,4-dihydropyridines as calcium channel antagonist. Bioorg. Med. Chem..

[27] Katouah H.A., Gaffer H.E. (2019). Synthesis and docking study of pyrimidine derivatives scaffold for anti-hypertension application. ChemistrySelect.

[28] Matos L.H.S., Masson F.T., Simeoni L.A., Homem-de-Mello M. (2018). Biological activity of dihydropyrimidinone (DHPM) derivatives: A systematic review. Eur. J. Med. Chem..

[29] Jain K.S., Bariwal J.B., Kathiravan M.K., Phoujdar M.S., Sahne R.S., Chauhan B.S., Shah A.K., Yadav M.R. (2008). Recent advances in selective α1-adrenoreceptor antagonists as antihypertensive agents. Bioorg. Med. Chem..

[30] Prakash G.S., Lau H., Panja C., Bychinskaya I., Ganesh S.K., Zaro B., Mathew T., Olah G.A. (2014). Synthesis of dihydropyrimidinones/thiopyrimidinones: Nafion-Ga, an efficient “green” lewis acid catalyst for the biginelli reaction. Catal. Lett..

[31] Zorkun I.S., Saraç S., Çelebi S., Erol K. (2006). Synthesis of 4-aryl-3,4-dihydropyrimidin-2 (1H)-thione derivatives as potential calcium channel blockers. Bioorg. Med. Chem..

[32] Mahgoub S., El-Sayed M.-I.K., El-Shehry M.F., Awad S.M., Mansour Y.E., Fatahala S.S. (2021). Synthesis of novel calcium channel blockers with ACE2 inhibition and dual antihypertensive/anti-inflammatory effects: A possible therapeutic tool for COVID-19. Bioorg. Chem..

[33] Teleb M., Zhang F.-X., Farghaly A.M., Wafa O.M.A., Fronczek F.R., Zamponi G.W., Fahmy H. (2017). Synthesis of new N3-substituted dihydropyrimidine derivatives as L-/T-type calcium channel blockers. Eur. J. Med. Chem..

[34] Cassambai S., Mee C.J., Renshaw D., Hussain A. (2019). Tiotropium bromide, a long acting muscarinic receptor antagonist triggers intracellular calcium signalling in the heart. Toxicol. Appl. Pharmacol..

[35] Johnson C.N., Pattanayek R., Potet F., Rebbeck R.T., Blackwell D.J., Nikolaienko R., Sequeira V., Le Meur R., Radwański P.B., Davis J.P.J.C.C. (2019). The CaMKII inhibitor KN93-calmodulin interaction and implications for calmodulin tuning of NaV1. 5 and RyR2 function. Cell Calcium.

[36] Tang L., Gamal El-Din T.M., Payandeh J., Martinez G.Q., Heard T.M., Scheuer T., Zheng N., Catterall W.A.J.N. (2014). Structural basis for Ca^2+^ selectivity of a voltage-gated calcium channel. Nature.

