# Supplementary material for: Design, Synthesis, Molecular Modeling, and Biological Evaluation of Novel Pyrimidine Derivatives as Potential Calcium Channel Blockers
Source: Molecules. 2023 Jun 20;28(12):4869. doi: 10.3390/molecules28124869 (PMC10303112; doi:10.3390/molecules28124869)
Supplement: Supplementary file 1 [file molecules-2382942-supplementary.pdf › molecules-2382942-supplementary.pdf]

# Supplementary Material

## Table of Contents

1H NMR and 13C NMR spectra copies

Ethyl 4-(1H-indol-3-yl)-6-methyl-2-thioxo-1,2,3,4-tetrahydropyrimidine-5-carboxylate 4a

ON DMSO D:\\ abar

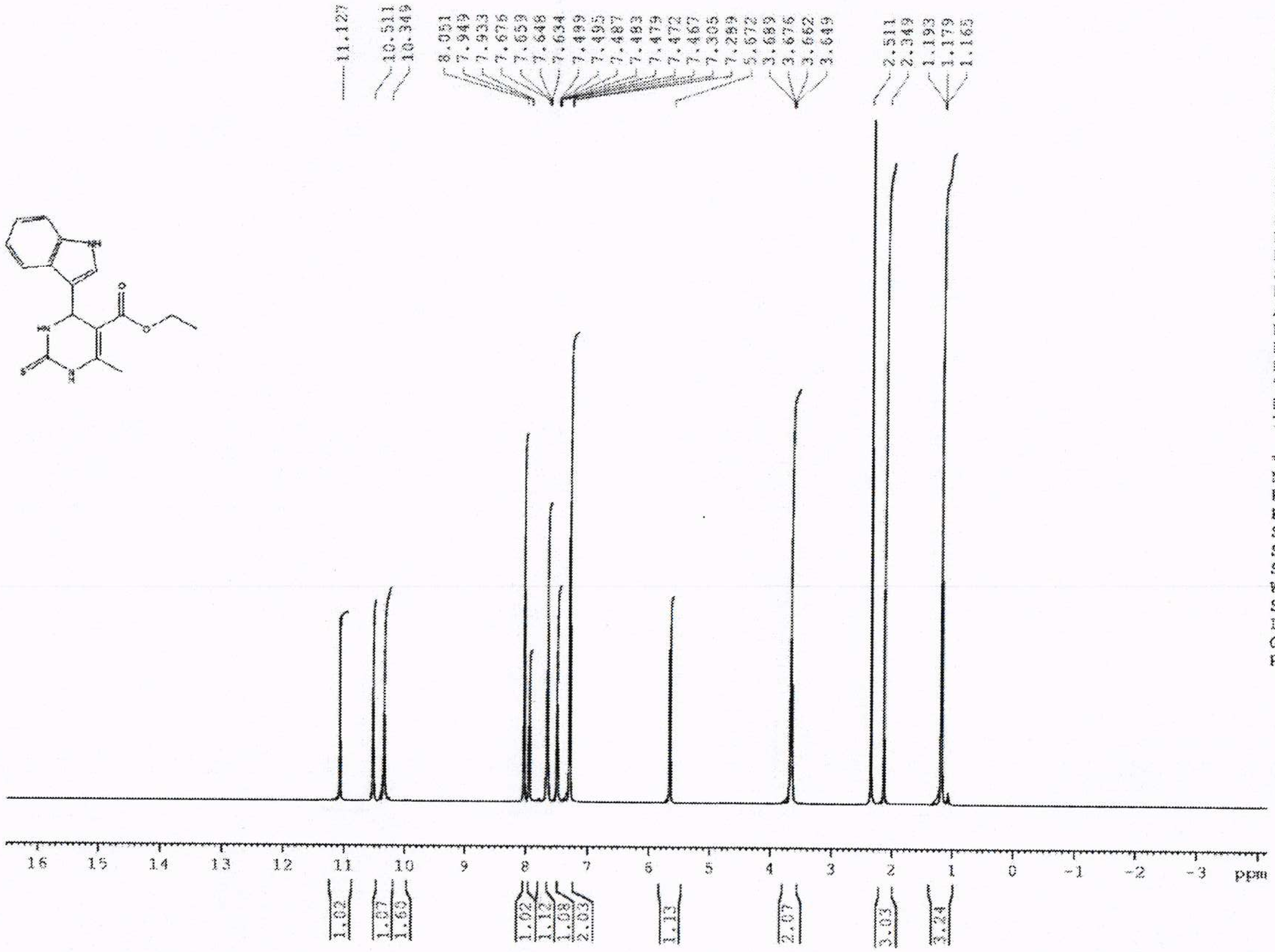


BRUKER

| | |
|---|---|
| NAME | drshahat-2 |
| EXPNO | 10 |
| PROCNO | 1 |
| Date_ | 20210624 |
| Time | 12.45 |
| INSTRUM | spect |
| PROBHD | 5 mm PABBO BB- |
| PULPROG | zg30 |
| TD | 65536 |
| SOLVENT | DMSO |
| NS | 16 |
| DS | 2 |
| SWH | 10330.578 Hz |
| FIDRES | 0.157632 Hz |
| AQ | 3.1720407 sec |
| RG | 35.9 |
| DW | 48.400 usec |
| DE | 6.50 usec |
| TE | 298.3 K |
| D1 | 1.00000000 sec |
| TD0 | 1 |

======== CHANNEL f1 ========

| | |
|---|---|
| NUC1 | 1H |
| P1 | 14.70 usec |
| PL1 | -1.10 dB |
| SFO1 | 500.1330885 MHz |
| SI | 32768 |
| SF | 500.1300000 MHz |
| WDW | EM |
| SSB | 0 |
| LB | 0.30 Hz |
| GB | 0 |
| PC | 1.00 |

C13CPD32 DMSO D:\\ abari 46

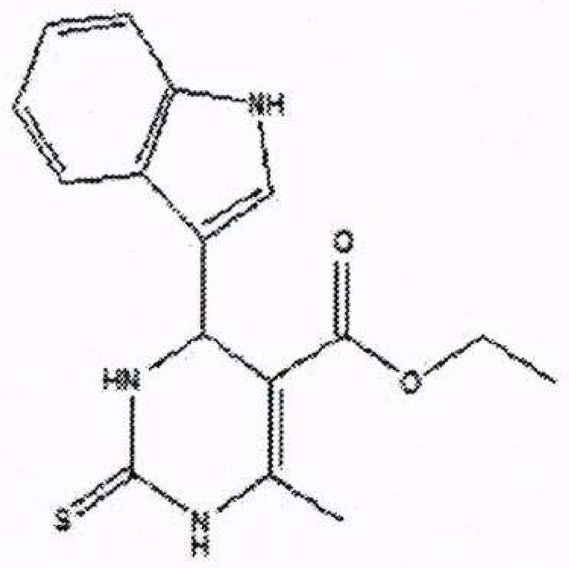

4a

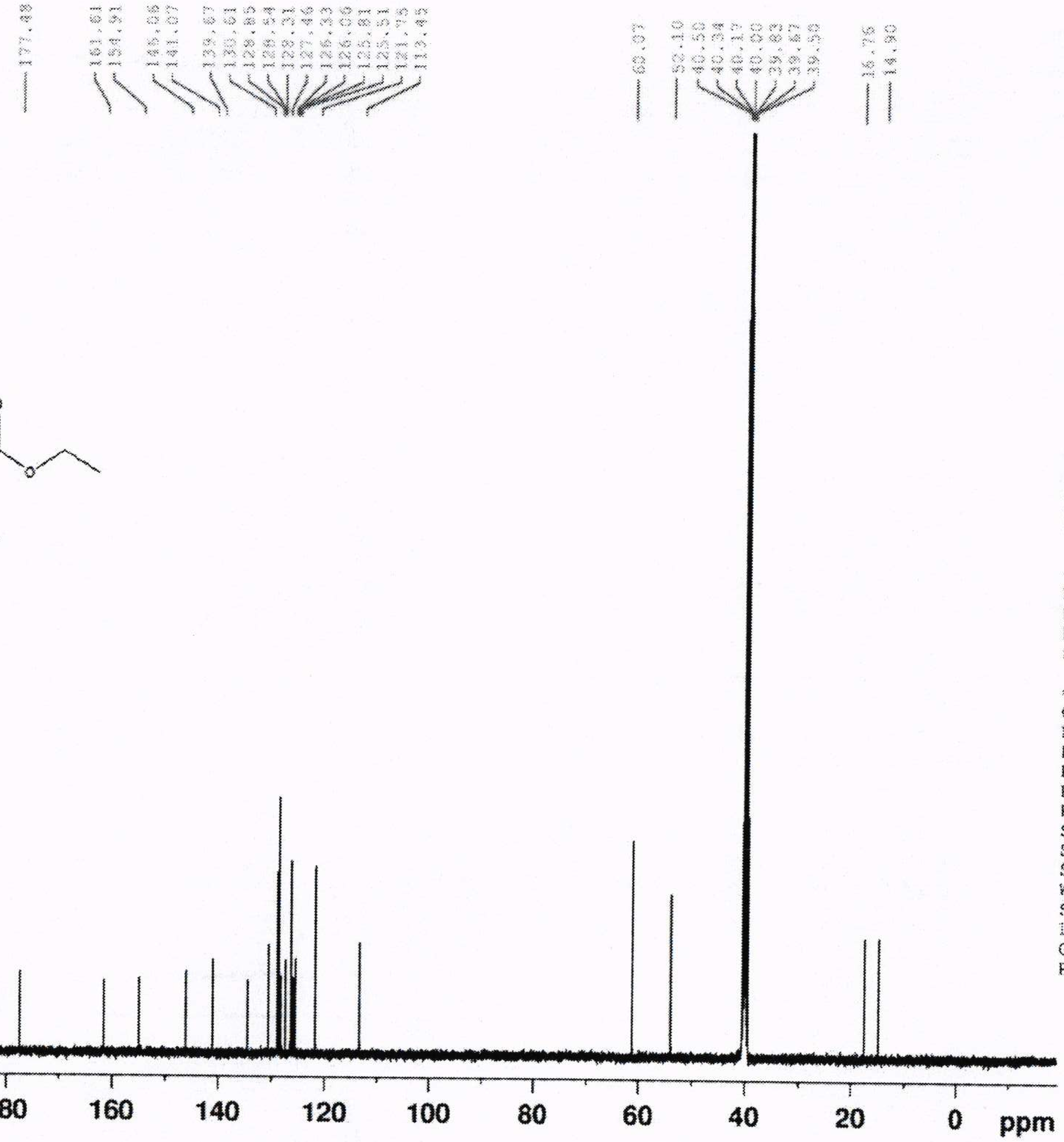


| | |
|---|---|
| NAME | drshahat-2 |
| EXPNO | 11 |
| PROCNO | 1 |
| Date_ | 20210624 |
| Time | 20.47 |
| INSTRUM | spect |
| PROBHD | 5 mm PABBO BB- |
| PULPROG | zgpg30 |
| TD | 65536 |
| SOLVENT | DMSO |
| NS | 2000 |
| DS | 2 |
| SWH | 30030.029 Hz |
| FIDRES | 0.458222 Hz |
| AQ | 1.0912410 sec |
| RG | 14596.5 |
| DW | 16.650 usec |
| DE | 6.50 usec |
| TE | 299.1 K |
| D1 | 2.00000000 sec |
| D11 | 0.03000000 sec |
| TD0 | 1 |

======== CHANNEL f1 ========

| | |
|---|---|
| NUC1 | 13C |
| P1 | 20.00 usec |
| PL1 | -6.00 dB |
| SFO1 | 125.7703643 MHz |

======== CHANNEL f2 ========

| | |
|---|---|
| CPDPRG2 | waltz16 |
| NUC2 | 1H |
| PCPD2 | 80.00 usec |
| PL2 | -1.10 dB |
| PL12 | 13.44 dB |
| PL13 | 16.40 dB |
| SFO2 | 500.1320005 MHz |
| SI | 32768 |
| SF | 125.7577890 MHz |
| WDW | EM |
| SSB | 0 |
| LB | 1.00 Hz |
| GB | 0 |
| PC | 1.40 |

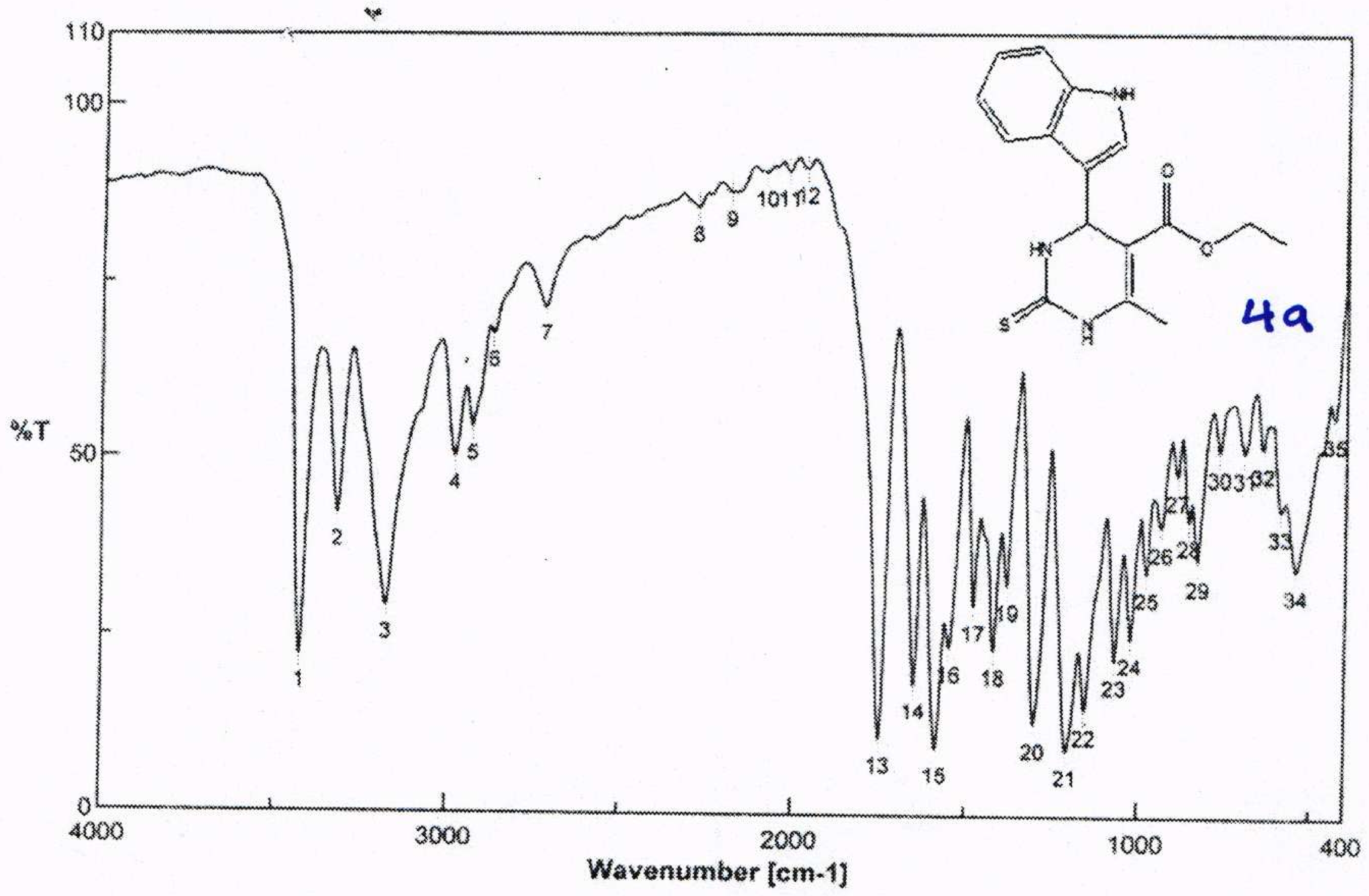


| | |
|---|---|
| Accumulation | 16 |
| Resolution | 4 cm-1 |
| Zero Filling | ON |
| Apodization | Cosine |
| Gain | Auto (4) |
| Scanning Speed | Auto (2 mm/sec) |
| Date/Time | 15/06/21 8:44Ö |
| Update | 15/06/21 8:45Ö |
| Operator | |
| File Name | Memory#27 |
| Sample Name | 1 |
| Comment | |

| No. | cm-1 | %T | No. | cm-1 | %T | No. | cm-1 | %T |
|---|---|---|---|---|---|---|---|---|
| 1 | 3425.92 | 22.2424 | 2 | 3319.86 | 42.1963 | 3 | 3178.11 | 29.2658 |
| 4 | 2980.45 | 50.2125 | 5 | 2930.31 | 54.382 | 6 | 2872.45 | 67.6564 |
| 7 | 2723 | 71.4615 | 8 | 2285.23 | 85.5904 | 9 | 2188.81 | 87.8301 |
| 10 | 2090.46 | 90.6215 | 11 | 2023.93 | 90.6816 | 12 | 1971.86 | 91.0992 |
| 13 | 1749.12 | 10.9233 | 14 | 1650.77 | 18.535 | 15 | 1586.16 | 9.60047 |
| 16 | 1549.52 | 23.6982 | 17 | 1480.1 | 29.5846 | 18 | 1422.24 | 23.3596 |
| 19 | 1383.68 | 32.5285 | 20 | 1302.68 | 13.0578 | 21 | 1207.22 | 9.40387 |
| 22 | 1155.15 | 14.9925 | 23 | 1070.3 | 21.991 | 24 | 1024.98 | 25.0683 |
| 25 | 978.697 | 34.4013 | 26 | 937.235 | 40.6767 | 27 | 891.916 | 47.9657 |
| 28 | 858.168 | 41.6919 | 29 | 831.169 | 35.9772 | 30 | 771.387 | 51.2849 |
| 31 | 698.105 | 51.1864 | 32 | 644.108 | 51.7501 | 33 | 592.039 | 43.0424 |
| 34 | 545.756 | 34.8429 | 35 | 434.869 | 55.9967 | | | |

## Cairo University
## Micro Analytical Center

***DI Analysis***
***Shimadzu Qp-2010 Plus***

| Sample Information | | Method | |
|---|---|---|---|
| Analyzed by | : Mai Younis | | |
| Analyzed | : 22/06/2021 02:44:23 ص | ——— Analytical Line 1 ——— | |
| Sample Name | : 2 | IonSourceTemp | : 250.00 °C |
| Sample ID | : | [MS Table] | |
| Customer Name | : Dr. Moh F | --Group 1 - Event 1-- | |
| Data File | : C:\GCMSsolution\Data\Project1\21.QGD | Start Time | : 0.00min |
| Org Data File | : C:\GCMSsolution\Data\Project1\21.QGD | End Time | : 10.00min |
| Method File | : C:\GCMSsolution\Data\Project1\Mai.qgm | ACQ Mode | : Scan |
| Org Method File | : C:\GCMSsolution\Data\Project1\Mai.qgm | Event Time | : 0.50sec |
| Report File | : | Scan Speed | : 1000 |
| Tuning File | : C:\GCMSsolution\System\Tune1_default1.qgt | Start m/z | : 50.00 |
| $EndIf$Modified by | : Mai Younis | End m/z | : 500.00 |
| Modified | : 22/06/2021 02:49:22 ص | | |
| | | Electron Voltage | : 70 eV |
| | | Ionization Mode | : EI |

C:\GCMSsolution\Data\Project1\21.QGD

Chromatogram 21 C:\GCMSsolution\Data\Project1\21.QGD

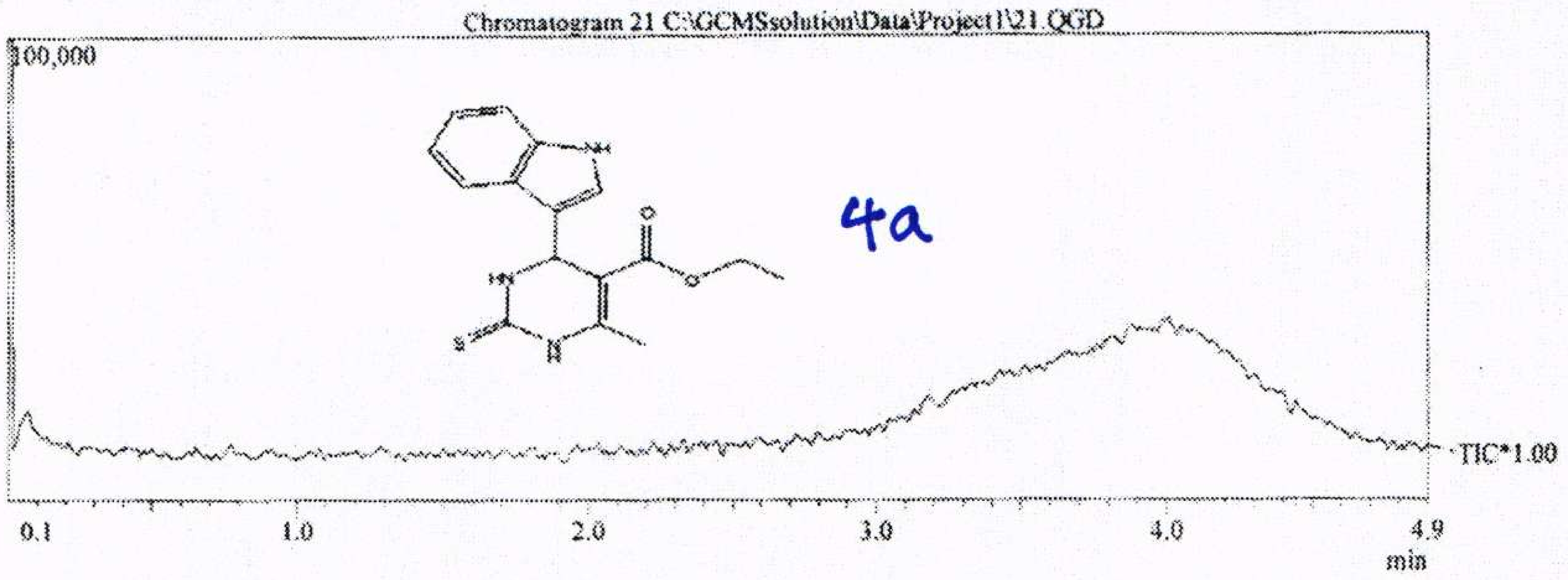


Spectrum

Line#:1 R.Time:3.1(Scan#:370)
MassPeaks: 315
RawMode:Single 3.1(370) BasePeak: 73(537)
BG Mode:None Group 1 - Event 1

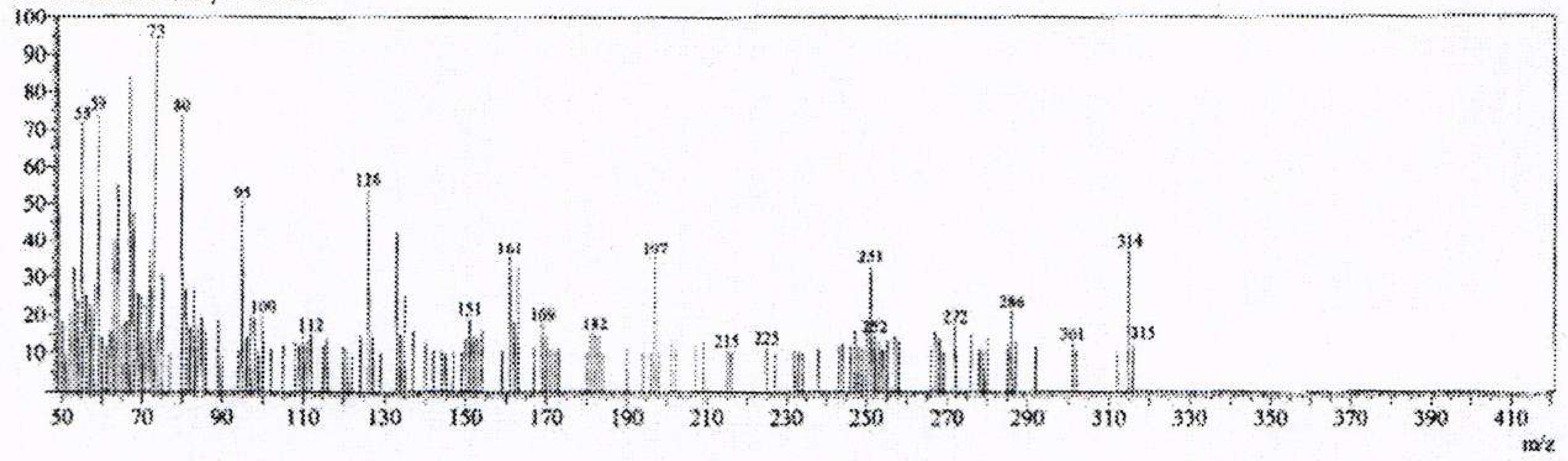


Mass Table
Line#:1 R.Time:3.1(Scan#:370)
MassPeaks: 315
RawMode:Single 3.1(370) BasePeak:73(537)
BG Mode:None Group 1 - Event 1

| # | m/z | Abs. In | Rel. Int. | # | m/z | Abs. In | Rel. Int. | # | m/z | Abs. In | Rel. Int. |
|---|---|---|---|---|---|---|---|---|---|---|---|
| 1 | 50.00 | 97 | 18.06 | 4 | 53.00 | 177 | 32.96 | 7 | 56.00 | 135 | 25.14 |
| 2 | 51.00 | 52 | 9.68 | 5 | 54.00 | 127 | 23.65 | 8 | 57.00 | 122 | 22.72 |
| 3 | 52.00 | 110 | 20.48 | 6 | 55.00 | 386 | 71.88 | 9 | 58.00 | 154 | 28.68 |

Ethyl 4-(2-chloroquinolin-3-yl)-6-methyl-2-thioxo-1,2,3,4-tetrahydropyrimidine-5-carboxylate 4b

ƆN DMSO D:\\ abar

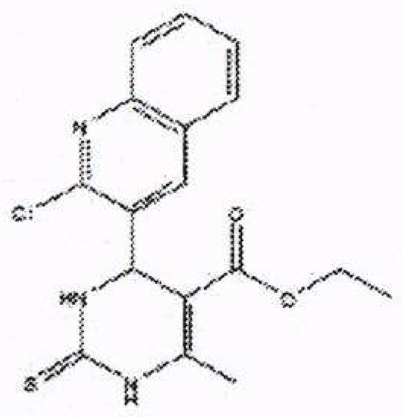

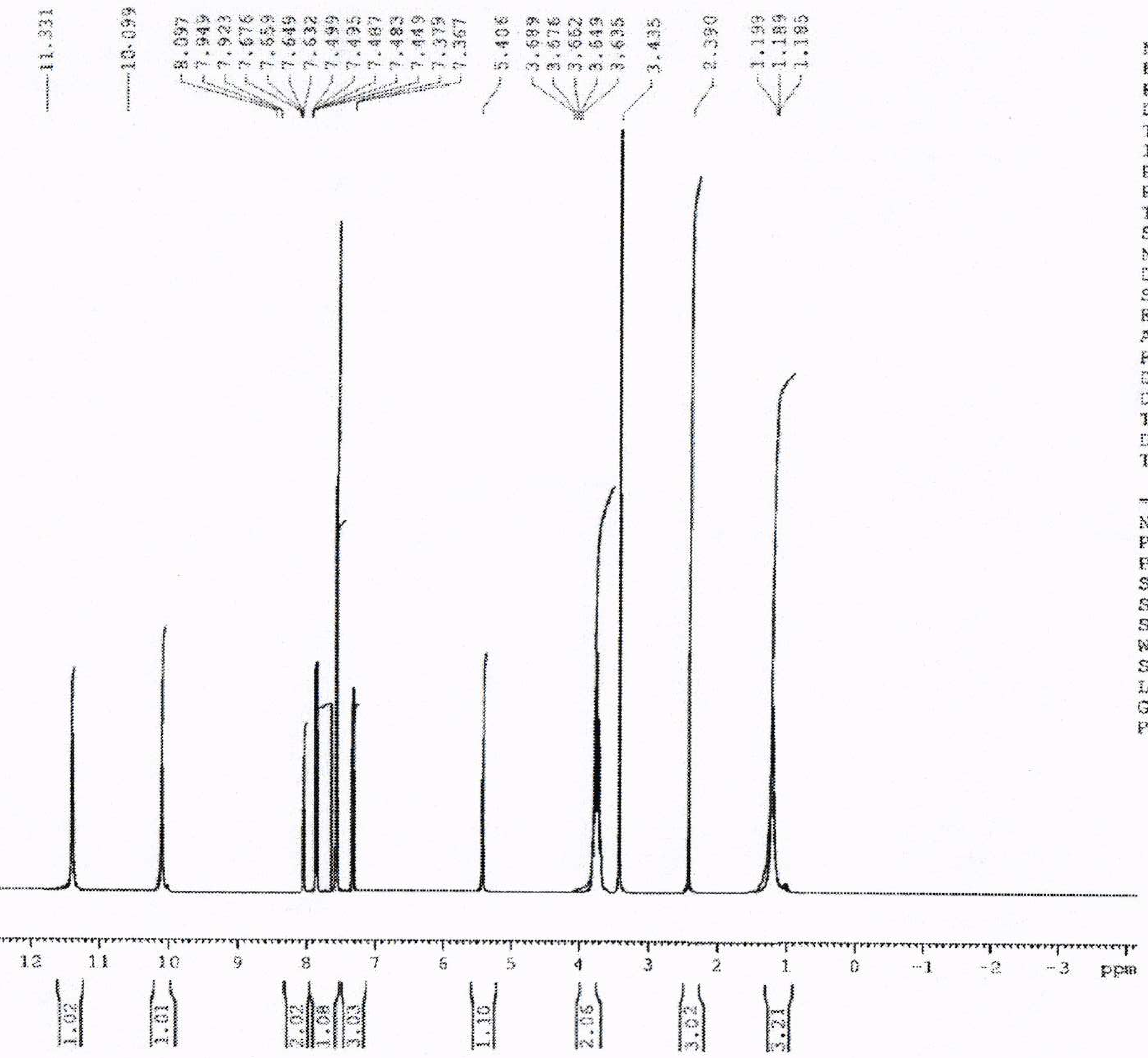


| | |
|---|---|
| NAME | drshahat-1 |
| EXPNO | 10 |
| PROCNO | 1 |
| Date_ | 20210624 |
| Time | 18.59 |
| INSTRUM | spect |
| PROBHD | 5 mm PABBO BB- |
| PULPROG | zg30 |
| TD | 65536 |
| SOLVENT | DMSO |
| NS | 16 |
| DS | 2 |
| SWH | 10330.578 Hz |
| FIDRES | 0.157632 Hz |
| AQ | 3.1720407 sec |
| RG | 90.5 |
| DW | 48.400 usec |
| DE | 6.50 usec |
| TE | 298.3 K |
| D1 | 1.00000000 sec |
| TD0 | 1 |

======== CHANNEL f1 ========

| | |
|---|---|
| NUC1 | 1H |
| P1 | 14.70 usec |
| PL1 | -1.10 dB |
| SFO1 | 500.1330885 MHz |
| SI | 32768 |
| SF | 500.1300000 MHz |
| WDW | EM |
| SSB | 0 |
| LB | 0.30 Hz |
| GB | 0 |
| PC | 1.00 |

C13CPD32 DMSO D:\\ abari 45

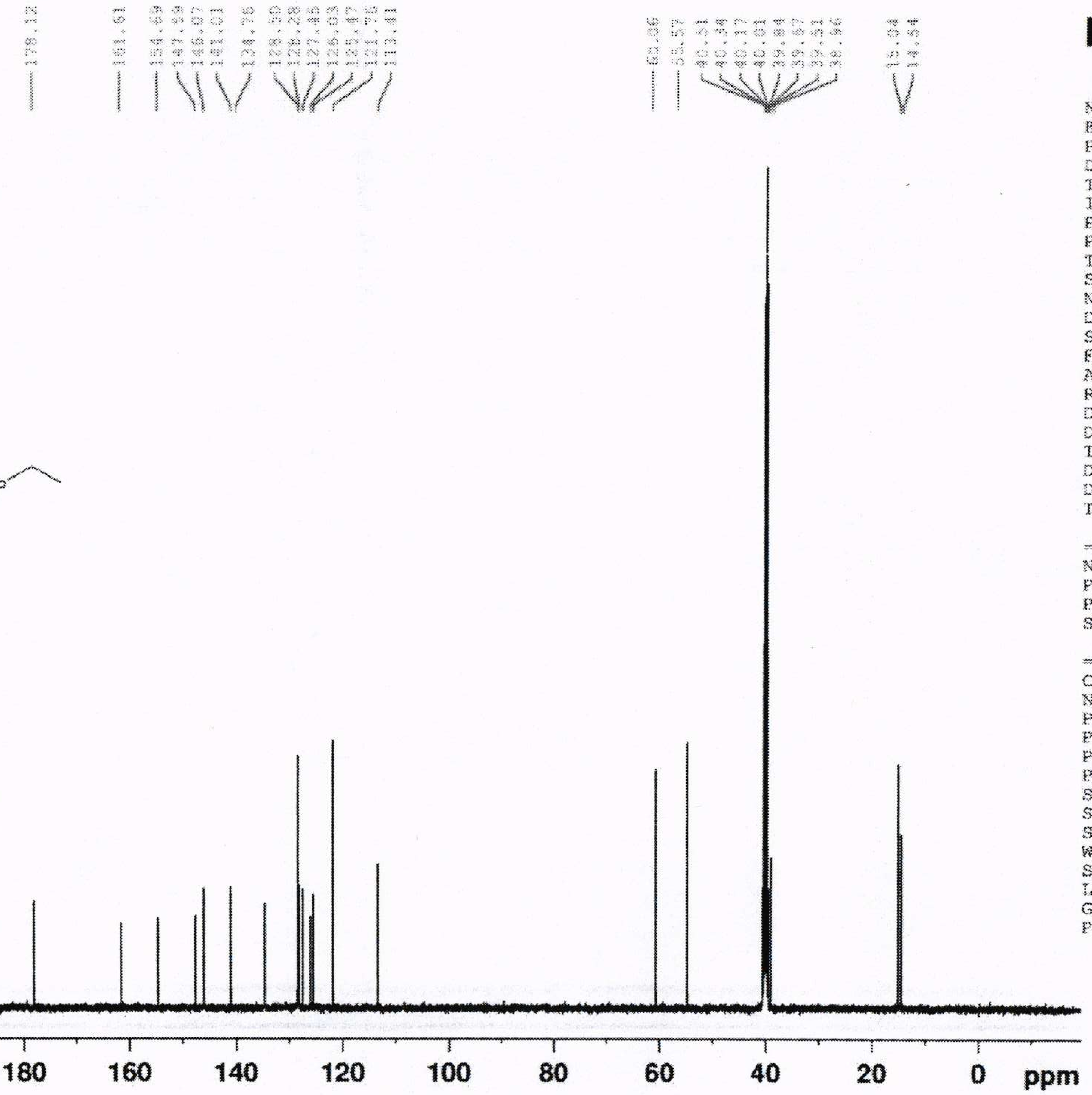


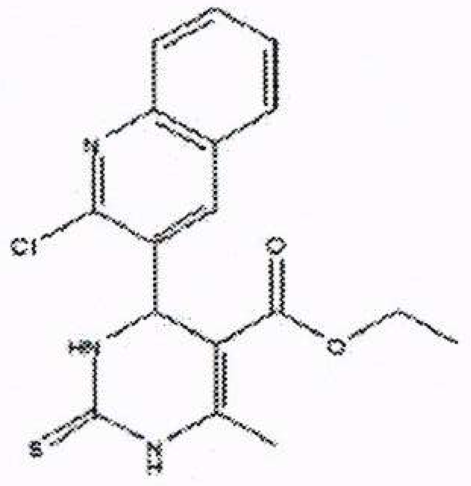


4b

BRUKER

| | |
|---|---|
| NAME | drshahat-1 |
| EXPNO | 11 |
| PROCNO | 1 |
| Date_ | 20210624 |
| Time | 13.45 |
| INSTRUM | spect |
| PROBHD | 5 mm PABBO BB- |
| PULPROG | zgpg30 |
| TD | 65536 |
| SOLVENT | DMSO |
| NS | 1104 |
| DS | 2 |
| SWH | 30030.029 Hz |
| FIDRES | 0.458222 Hz |
| AQ | 1.0912410 sec |
| RG | 2048 |
| DW | 16.650 usec |
| DE | 6.50 usec |
| TE | 299.0 K |
| D1 | 2.00000000 sec |
| D11 | 0.03000000 sec |
| TD0 | 1 |

======== CHANNEL f1 ========

| | |
|---|---|
| NUC1 | 13C |
| P1 | 20.00 usec |
| PL1 | -6.00 dB |
| SFO1 | 125.7703643 MHz |

======== CHANNEL f2 ========

| | |
|---|---|
| CPDPRG2 | waltz16 |
| NUC2 | 1H |
| PCPD2 | 80.00 usec |
| PL2 | -1.10 dB |
| PL12 | 13.44 dB |
| PL13 | 16.40 dB |
| SFO2 | 500.1320005 MHz |
| SI | 32768 |
| SF | 125.7577890 MHz |
| WDW | EM |
| SSB | 0 |
| LB | 1.00 Hz |
| GB | 0 |
| PC | 1.40 |

Ethyl 4-(1,3-diphenyl-1H-pyrazol-4-yl)-6-methyl-2-thioxo-1,2,3,4-tetrahydropyrimidine-5-carboxylate 4c

ON DMSO D:\\ abar

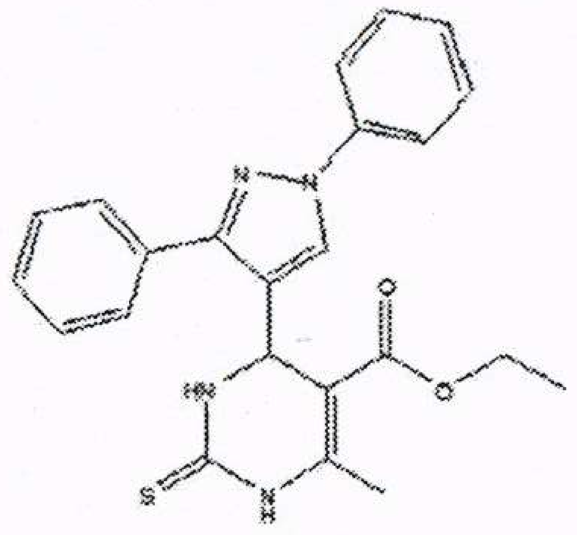

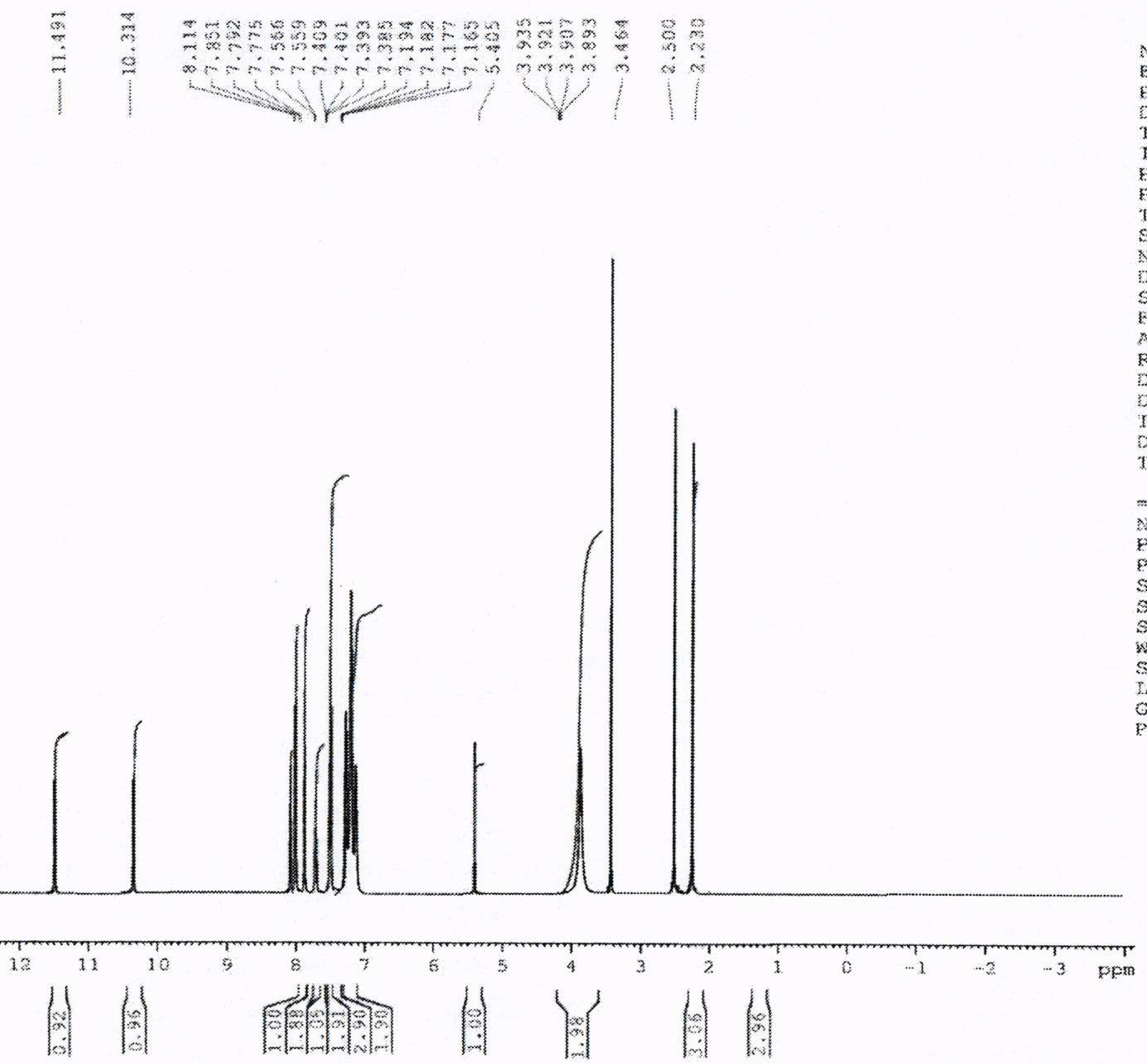


| | | |
|---|---|---|
| NAME | drshahat-3 | |
| EXPNO | 10 | |
| PROCNO | 1 | |
| Date_ | 20210624 | |
| Time | 11.17 | |
| INSTRUM | spect | |
| PROBHD | 5 mm PABBO BB- | |
| PULPROG | zg30 | |
| TD | 65536 | |
| SOLVENT | DMSO | |
| NS | 16 | |
| DS | 2 | |
| SWH | 10330.578 | Hz |
| FIDRES | 0.157632 | Hz |
| AQ | 3.1720407 | sec |
| RG | 128 | |
| DW | 48.400 | usec |
| DE | 6.50 | usec |
| TE | 298.3 | K |
| D1 | 1.00000000 | sec |
| TD0 | 1 | |

======== CHANNEL f1 ========

| | | |
|---|---|---|
| NUC1 | 1H | |
| P1 | 14.70 | usec |
| PL1 | -1.10 | dB |
| SFO1 | 500.1330885 | MHz |
| SI | 32768 | |
| SF | 500.1300000 | MHz |
| WDW | EM | |
| SSB | 0 | |
| LB | 0.30 | Hz |
| GB | 0 | |
| PC | 1.00 | |

C13CPD32 DMSO D:\\ abari 44

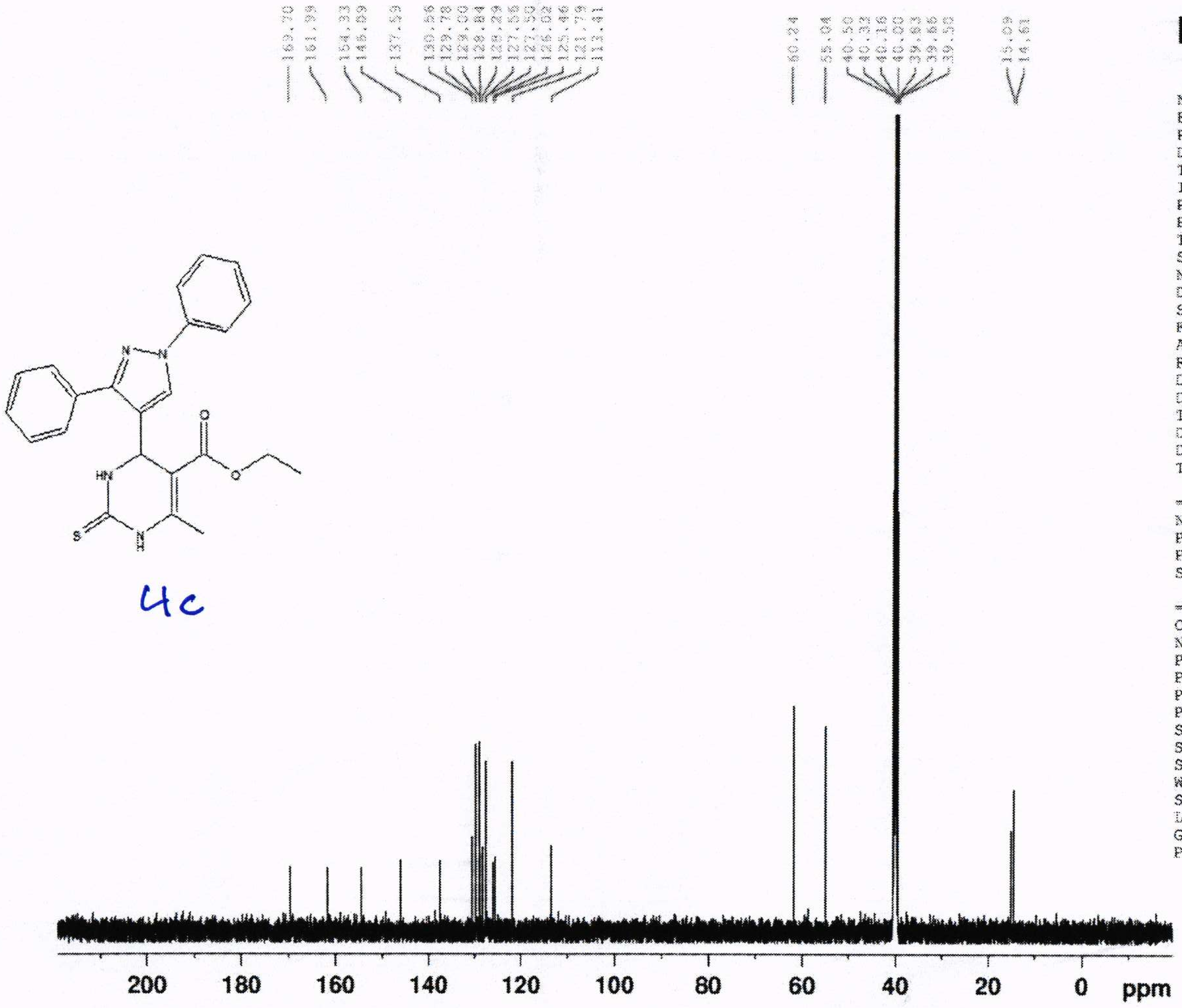


BRUKER

| | |
|---|---|
| NAME | drshahat-3 |
| EXPNO | 11 |
| PROCNO | 1 |
| Date_ | 20210624 |
| Time | 12.07 |
| INSTRUM | spect |
| PROBHD | 5 mm PABBO BB- |
| PULPROG | zgpg30 |
| TD | 65536 |
| SOLVENT | DMSO |
| NS | 1165 |
| DS | 2 |
| SWH | 30030.029 Hz |
| FIDRES | 0.458222 Hz |
| AQ | 1.0912410 sec |
| RG | 4597.6 |
| DW | 16.650 usec |
| DE | 6.50 usec |
| TE | 299.2 K |
| D1 | 2.00000000 sec |
| D11 | 0.03000000 sec |
| TD0 | 1 |
| ======== CHANNEL f1 ======== | |
| NUC1 | 13C |
| P1 | 20.00 usec |
| PL1 | -6.00 dB |
| SFO1 | 125.7703643 MHz |
| ======== CHANNEL f2 ======== | |
| CPDPRG2 | waltz16 |
| NUC2 | 1H |
| PCPD2 | 80.00 usec |
| PL2 | -1.10 dB |
| PL12 | 13.44 dB |
| PL13 | 16.40 dB |
| SFO2 | 500.1320005 MHz |
| SI | 32768 |
| SF | 125.7577890 MHz |
| WDW | EM |
| SSB | 0 |
| LB | 1.00 Hz |
| GB | 0 |
| PC | 1.40 |

4-(1H-Indol-3-yl)-6-methyl-2-thioxo-1,2,3,4-tetrahydropyrimidine-5-carboxylic acid 5a

ON DMSO D:\\ abar

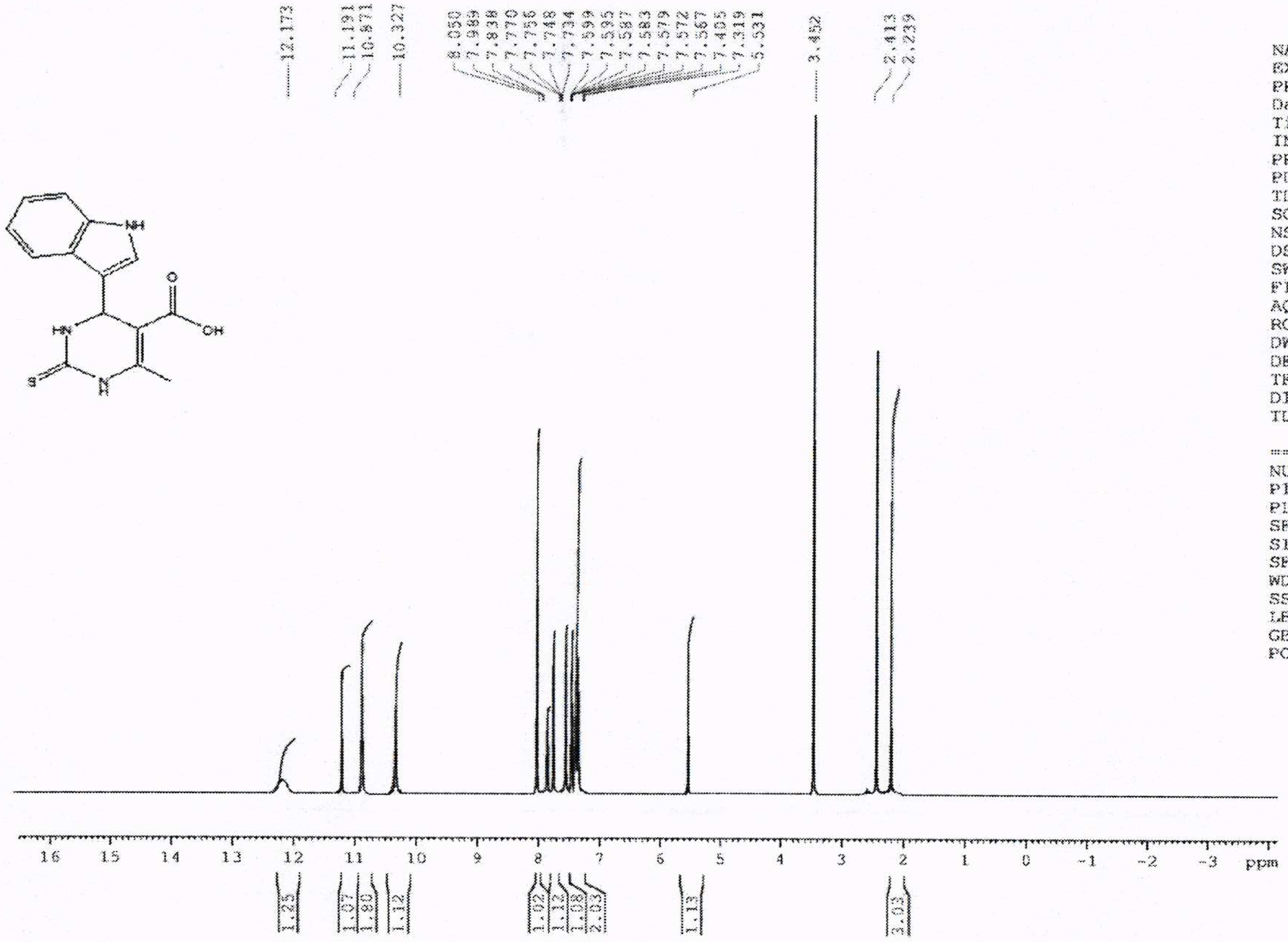


BRUKER

| | |
|---|---|
| NAME | drshahat-5 |
| EXPNO | 10 |
| PROCNO | 1 |
| Date_ | 20210624 |
| Time | 18.59 |
| INSTRUM | spect |
| PROBHD | 5 mm PABBO BB- |
| PULPROG | zg30 |
| TD | 65536 |
| SOLVENT | DMSO |
| NS | 16 |
| DS | 2 |
| SWH | 10330.578 Hz |
| FIDRES | 0.157632 Hz |
| AQ | 3.1720407 sec |
| RG | 90.5 |
| DW | 48.400 usec |
| DE | 6.50 usec |
| TE | 298.3 K |
| D1 | 1.00000000 sec |
| TD0 | 1 |

======== CHANNEL f1 ========

| | |
|---|---|
| NUC1 | 1H |
| P1 | 14.70 usec |
| PL1 | -1.10 dB |
| SFO1 | 500.1330885 MHz |
| SI | 32768 |
| SF | 500.1300000 MHz |
| WDW | EM |
| SSB | 0 |
| LB | 0.30 Hz |
| GB | 0 |
| PC | 1.00 |

C13CPD32 DMSO D:\\ abari 46

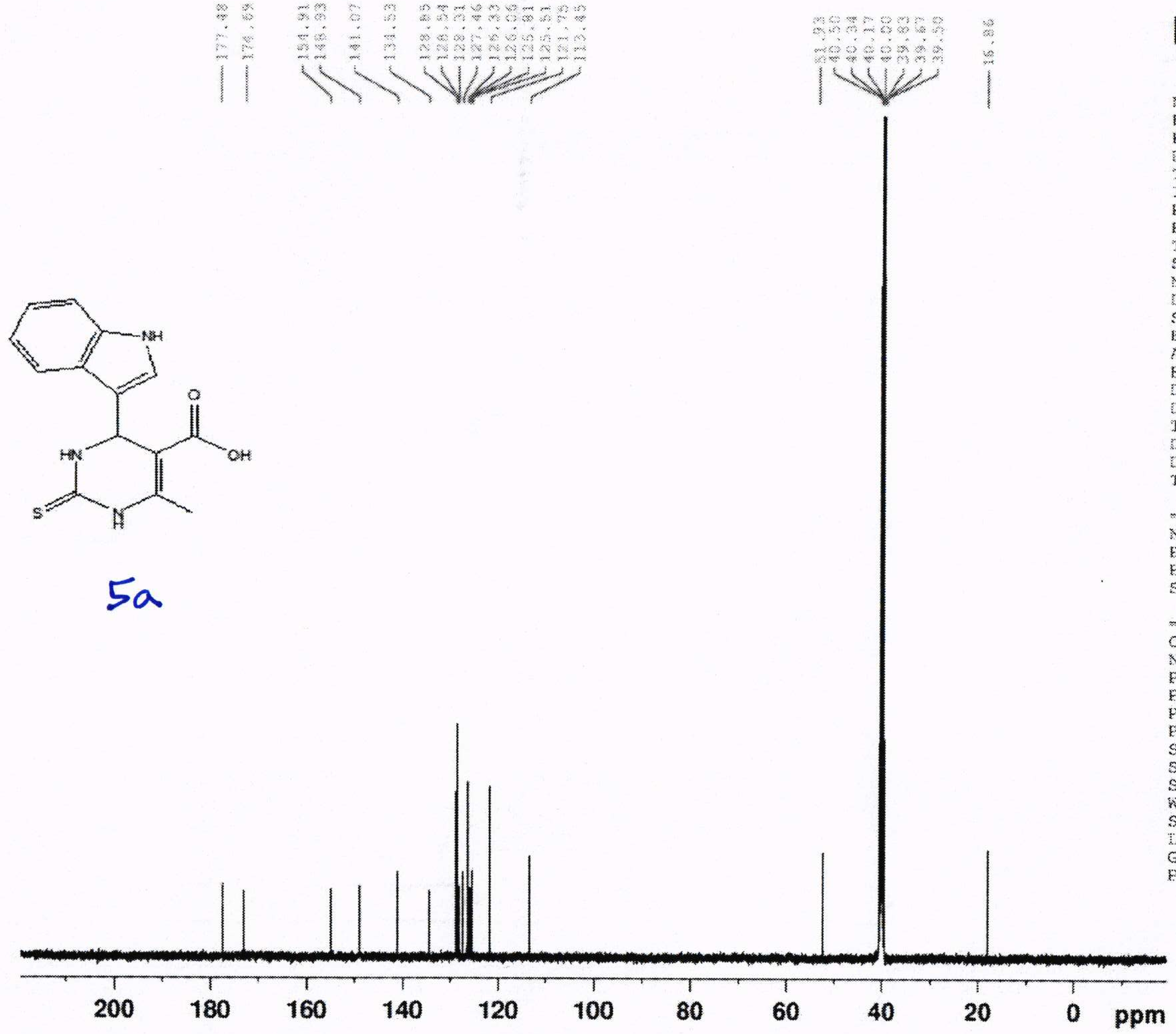


5a

BRUKER

| | |
|---|---|
| NAME | drshahat-5 |
| EXPNO | 11 |
| PROCNO | 1 |
| Date_ | 20210624 |
| Time | 20.47 |
| INSTRUM | spect |
| PROBHD | 5 mm PABBO BB- |
| PULPROG | zgpg30 |
| TD | 65536 |
| SOLVENT | DMSO |
| NS | 2000 |
| DS | 2 |
| SWH | 30030.029 Hz |
| FIDRES | 0.458222 Hz |
| AQ | 1.0912410 sec |
| RG | 14596.5 |
| DW | 16.650 usec |
| DE | 6.50 usec |
| TE | 299.1 K |
| D1 | 2.00000000 sec |
| D11 | 0.03000000 sec |
| TD0 | 1 |

======== CHANNEL f1 ========

| | |
|---|---|
| NUC1 | 13C |
| P1 | 20.00 usec |
| PL1 | -6.00 dB |
| SFO1 | 125.7703643 MHz |

======== CHANNEL f2 ========

| | |
|---|---|
| CPDPRG2 | waltz16 |
| NUC2 | 1H |
| PCPD2 | 80.00 usec |
| PL2 | -1.10 dB |
| PL12 | 13.44 dB |
| PL13 | 16.40 dB |
| SFO2 | 500.1320005 MHz |
| SI | 32768 |
| SF | 125.7577890 MHz |
| WDW | EM |
| SSB | 0 |
| LB | 1.00 Hz |
| GB | 0 |
| PC | 1.40 |

4-(2-Chloroquinolin-3-yl)-6-methyl-2-thioxo-1,2,3,4-tetrahydropyrimidine-5-carboxylic acid 5b

ON DMSO D:\\ abar

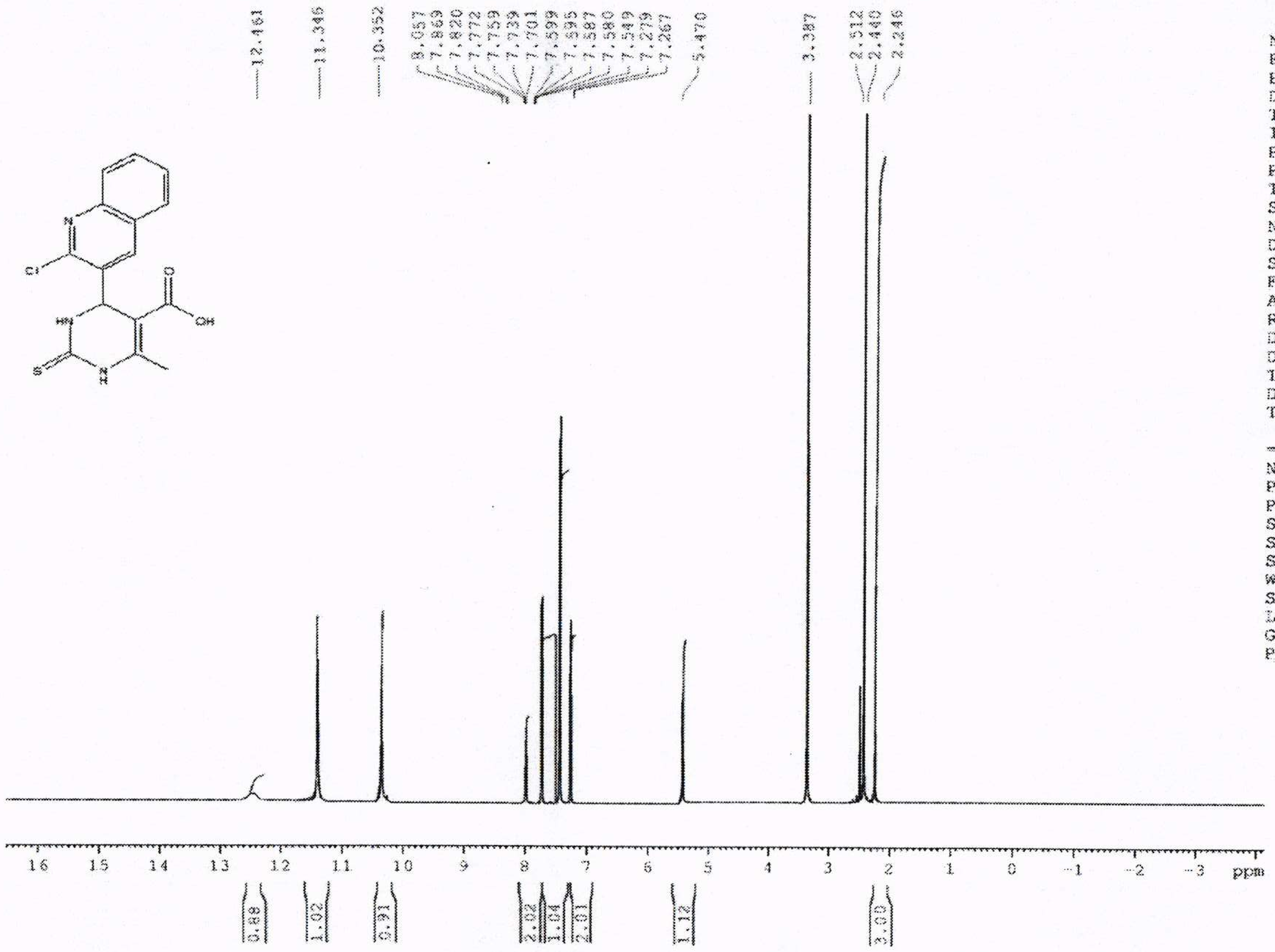


| | | |
|---|---|---|
| NAME | drshahat-4 | |
| EXPNO | 10 | |
| PROCNO | 1 | |
| Date_ | 20210624 | |
| Time | 18.59 | |
| INSTRUM | spect | |
| PROBHD | 5 mm PABBO BB- | |
| PULPROG | zg30 | |
| TD | 65536 | |
| SOLVENT | DMSO | |
| NS | 16 | |
| DS | 2 | |
| SWH | 10330.578 | Hz |
| FIDRES | 0.157632 | Hz |
| AQ | 3.1720407 | sec |
| RG | 90.5 | |
| DW | 48.400 | usec |
| DE | 6.50 | usec |
| TE | 298.3 | K |
| D1 | 1.00000000 | sec |
| TD0 | 1 | |

======== CHANNEL f1 ========

| | | |
|---|---|---|
| NUC1 | 1H | |
| P1 | 14.70 | usec |
| PL1 | -1.10 | dB |
| SFO1 | 500.1330885 | MHz |
| SI | 32768 | |
| SF | 500.1300000 | MHz |
| WDW | EM | |
| SSB | 0 | |
| LB | 0.30 | Hz |
| GB | 0 | |
| PC | 1.00 | |

C13CPD32 DMSO D:\\ abari 45

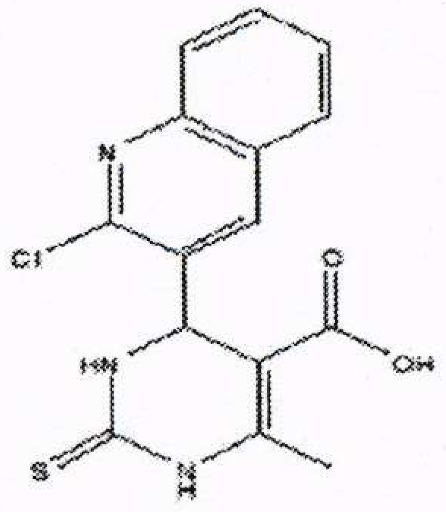

5b

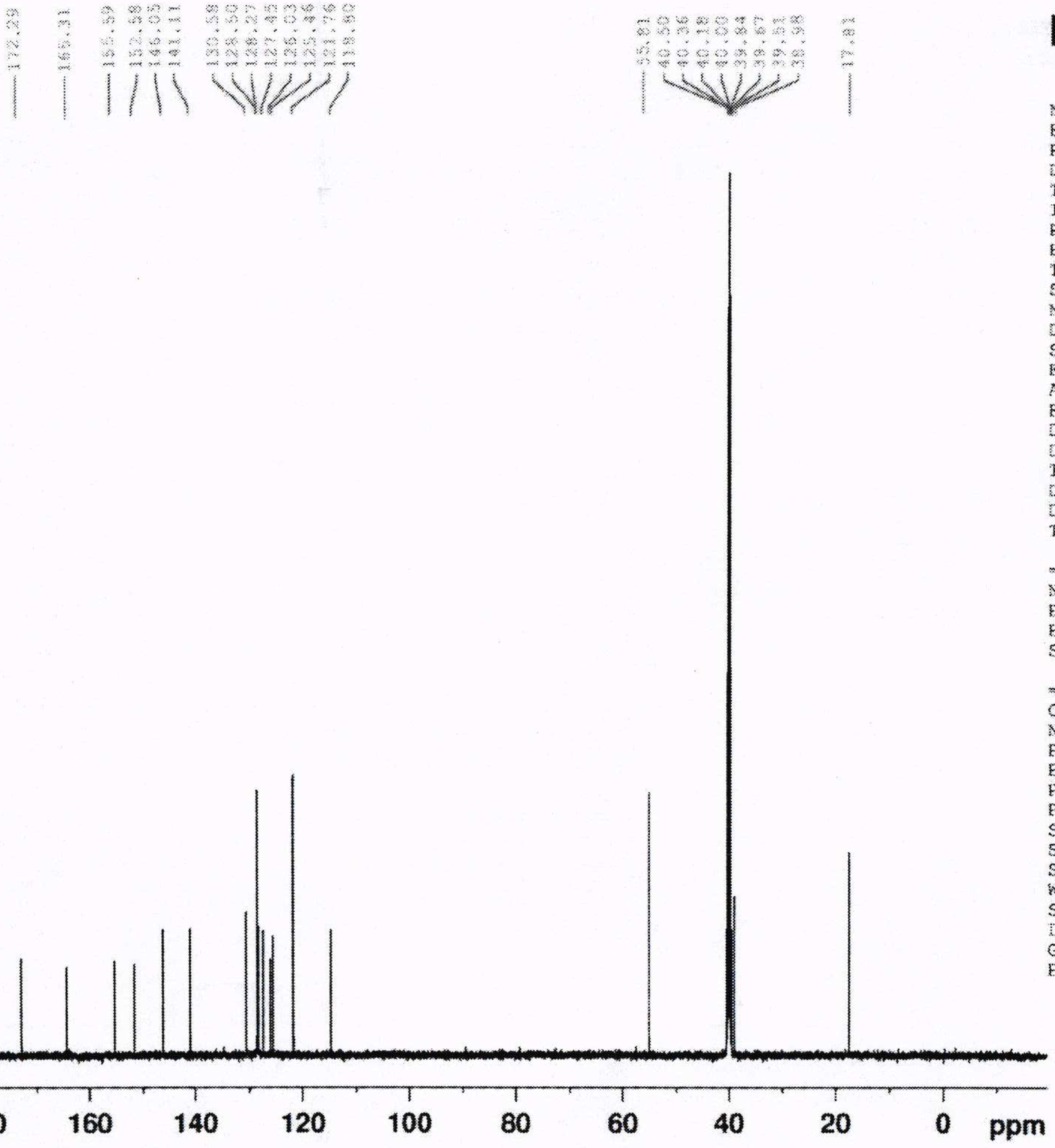


| | |
|---|---|
| NAME | drshahat-4 |
| EXPNO | 11 |
| PROCNO | 1 |
| Date_ | 20210624 |
| Time | 13.45 |
| INSTRUM | spect |
| PROBHD | 5 mm PABBO BB- |
| PULPROG | zgpg30 |
| TD | 65536 |
| SOLVENT | DMSO |
| NS | 1104 |
| DS | 2 |
| SWH | 30030.029 Hz |
| FIDRES | 0.458222 Hz |
| AQ | 1.0912410 sec |
| RG | 2048 |
| DW | 16.650 usec |
| DE | 6.50 usec |
| TE | 299.0 K |
| D1 | 2.00000000 sec |
| D11 | 0.03000000 sec |
| TD0 | 1 |

======== CHANNEL f1 ========

| | |
|---|---|
| NUC1 | 13C |
| P1 | 20.00 usec |
| PL1 | -6.00 dB |
| SFO1 | 125.7703643 MHz |

======== CHANNEL f2 ========

| | |
|---|---|
| CPDPRG2 | waltz16 |
| NUC2 | 1H |
| PCPD2 | 80.00 usec |
| PL2 | -1.10 dB |
| PL12 | 13.44 dB |
| PL13 | 16.40 dB |
| SFO2 | 500.1320005 MHz |
| SI | 32768 |
| SF | 125.7577890 MHz |
| WDW | EM |
| SSB | 0 |
| LB | 1.00 Hz |
| GB | 0 |
| PC | 1.40 |

4-(1,3-Diphenyl-1H-pyrazol-4-yl)-6-methyl-2-thioxo-1,2,3,4-tetrahydropyrimidine-5-carboxylic acid 5c

ɔN DMSO D:\\ abar

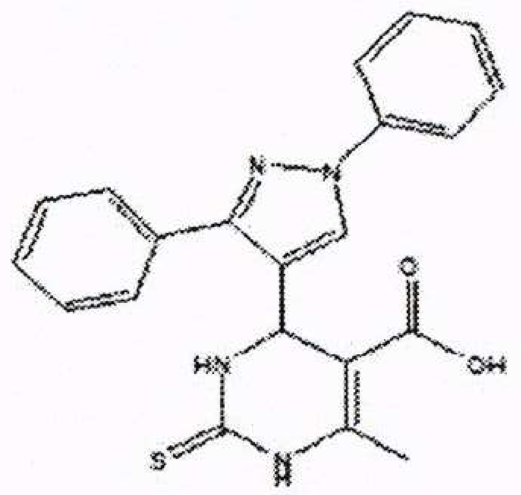

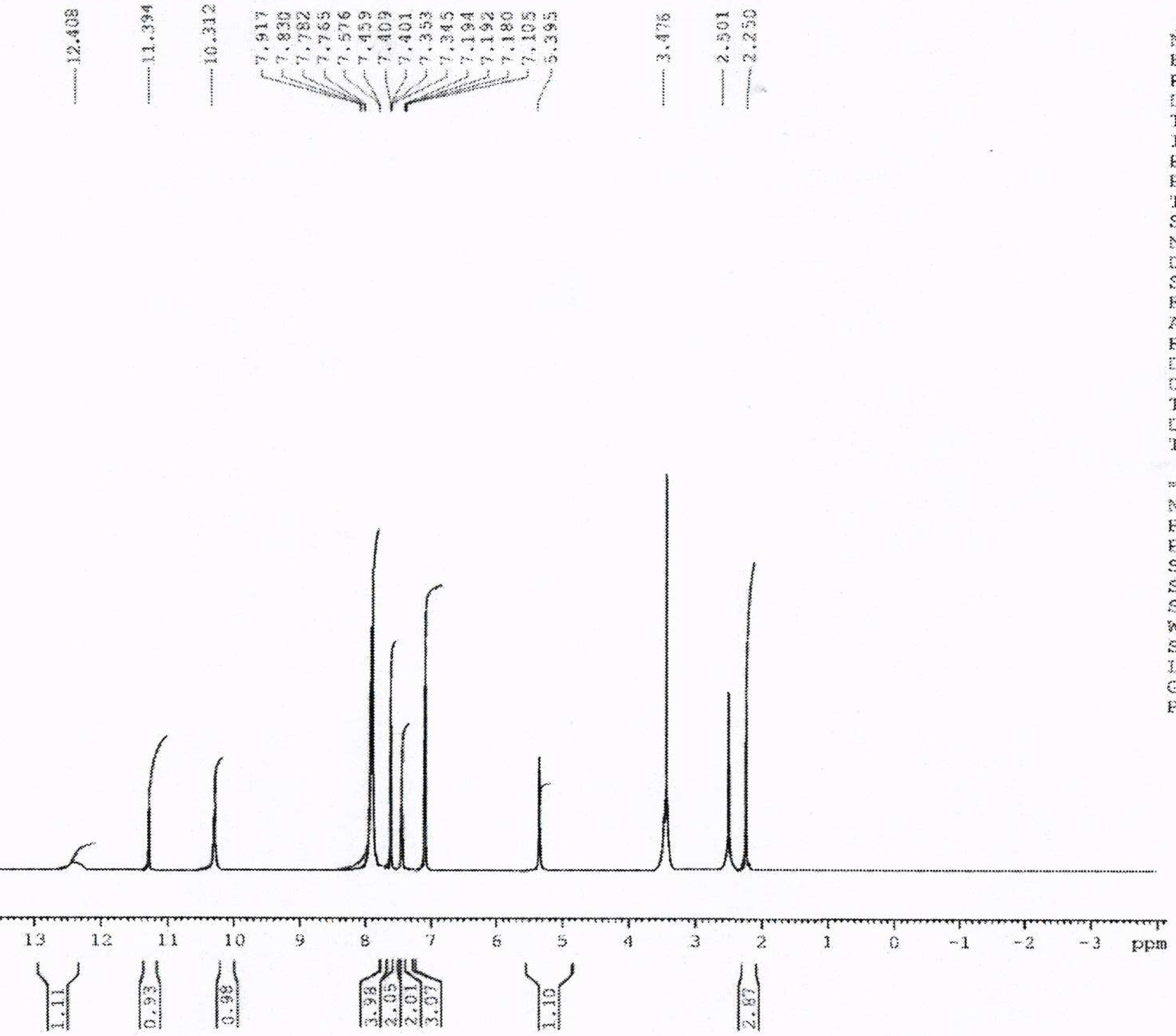


| | |
|---|---|
| NAME | drshahat-6 |
| EXPNO | 10 |
| PROCNO | 1 |
| Date_ | 20210624 |
| Time | 11.17 |
| INSTRUM | spect |
| PROBHD | 5 mm PABBO BB- |
| PULPROG | zg30 |
| TD | 65536 |
| SOLVENT | DMSO |
| NS | 16 |
| DS | 2 |
| SWH | 10330.578 Hz |
| FIDRES | 0.157632 Hz |
| AQ | 3.1720407 sec |
| RG | 128 |
| DW | 48.400 usec |
| DE | 6.50 usec |
| TE | 298.3 K |
| D1 | 1.00000000 sec |
| TD0 | 1 |

======== CHANNEL f1 ========

| | |
|---|---|
| NUC1 | 1H |
| P1 | 14.70 usec |
| PL1 | -1.10 dB |
| SFO1 | 500.1330885 MHz |
| SI | 32768 |
| SF | 500.1300000 MHz |
| WDW | EM |
| SSB | 0 |
| LB | 0.30 Hz |
| GB | 0 |
| PC | 1.00 |

C13CPD32 DMSO D:\\ abari 44

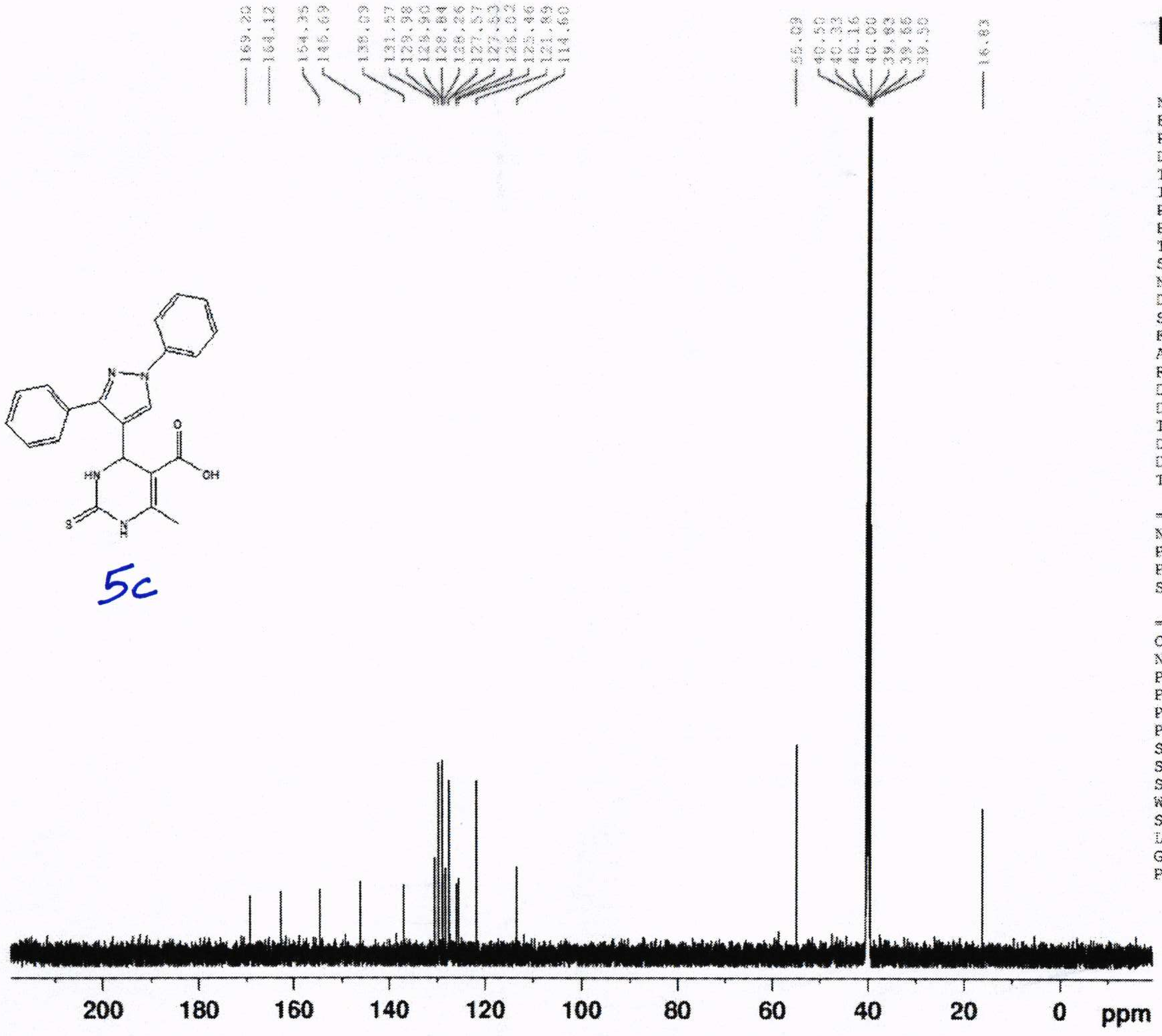


5c

BRUKER

| | |
|---|---|
| NAME | drshahat-6 |
| EXPNO | 11 |
| PROCNO | 1 |
| Date_ | 20210624 |
| Time | 12.07 |
| INSTRUM | spect |
| PROBHD | 5 mm PABBO BB- |
| PULPROG | zgpg30 |
| TD | 65536 |
| SOLVENT | DMSO |
| NS | 1165 |
| DS | 2 |
| SWH | 30030.029 Hz |
| FIDRES | 0.458222 Hz |
| AQ | 1.0912410 sec |
| RG | 4597.6 |
| DW | 16.650 usec |
| DE | 6.50 usec |
| TE | 299.2 K |
| D1 | 2.00000000 sec |
| D11 | 0.03000000 sec |
| TD0 | 1 |
| ======== CHANNEL f1 ======== | |
| NUC1 | 13C |
| P1 | 20.00 usec |
| PL1 | -6.00 dB |
| SFO1 | 125.7703643 MHz |
| ======== CHANNEL f2 ======== | |
| CPDPRG2 | waltz16 |
| NUC2 | 1H |
| PCPD2 | 80.00 usec |
| PL2 | -1.10 dB |
| PL12 | 13.44 dB |
| PL13 | 16.40 dB |
| SFO2 | 500.1320005 MHz |
| SI | 32768 |
| SF | 125.7577890 MHz |
| WDW | EM |
| SSB | 0 |
| LB | 1.00 Hz |
| GB | 0 |
| PC | 1.40 |

4-(1H-Indol-3-yl)-6-methyl-2-thioxo-1,2,3,4-tetrahydropyrimidine-5-carbonyl chloride 6a

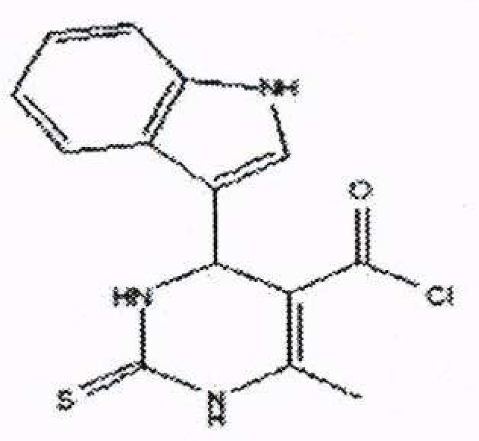

ON DMSO D:\\ abar

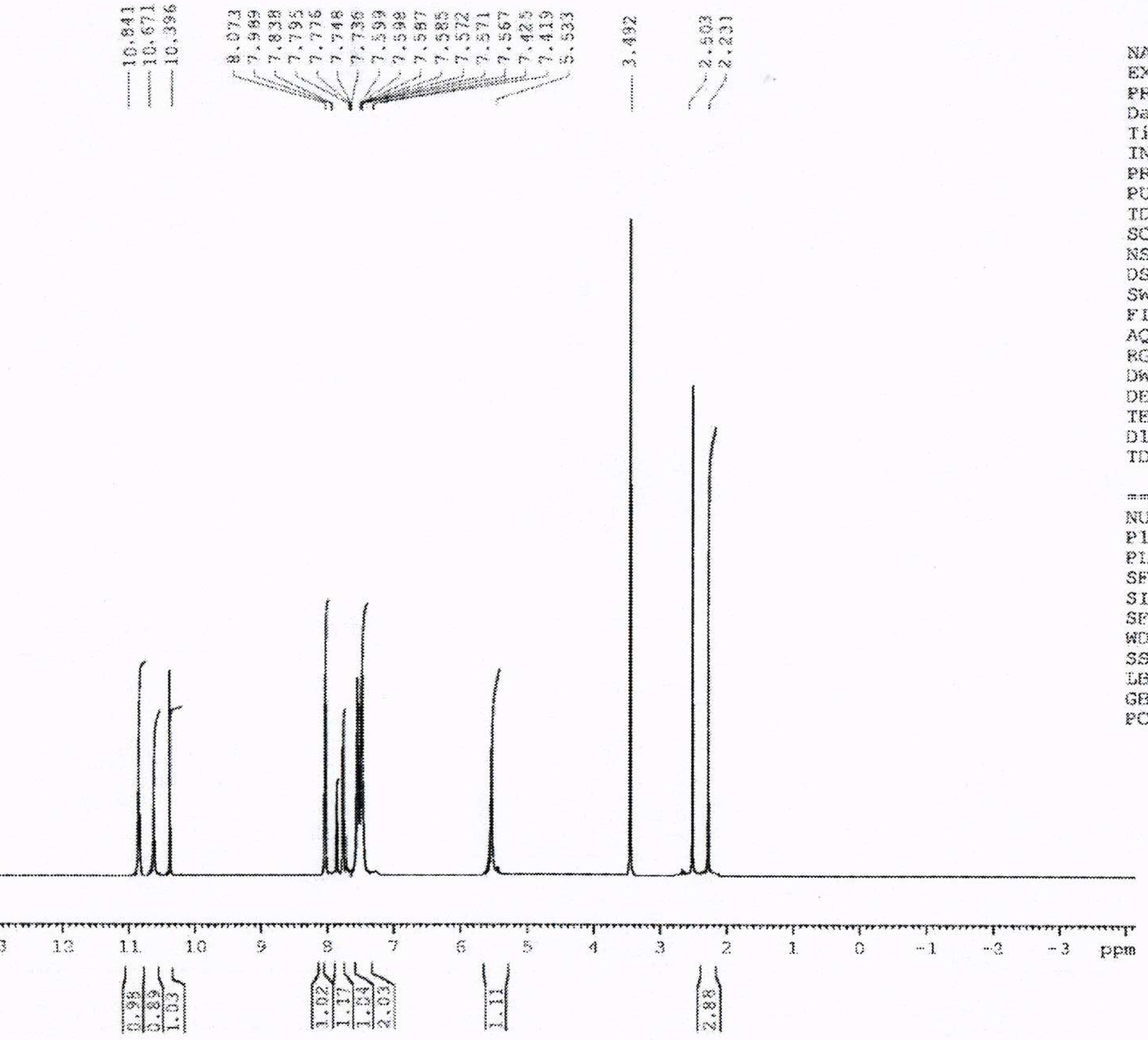


BRUKER

| | | |
|---|---|---|
| NAME | drshahat-8 | |
| EXPNO | 10 | |
| PROCNO | 1 | |
| Date_ | 20210625 | |
| Time | 18.59 | |
| INSTRUM | spect | |
| PROBHD | 5 mm PABBO BB- | |
| PULPROG | zg30 | |
| TD | 65536 | |
| SOLVENT | DMSO | |
| NS | 16 | |
| DS | 2 | |
| SWH | 10330.578 | Hz |
| FIDRES | 0.157632 | Hz |
| AQ | 3.1720407 | sec |
| RG | 90.5 | |
| DW | 48.400 | usec |
| DE | 6.50 | usec |
| TE | 298.3 | K |
| D1 | 1.00000000 | sec |
| TD0 | 1 | |

======== CHANNEL f1 ========

| | | |
|---|---|---|
| NUC1 | 1H | |
| P1 | 14.70 | usec |
| PL1 | -1.10 | dB |
| SFO1 | 500.1330885 | MHz |
| SI | 32768 | |
| SF | 500.1300000 | MHz |
| WDW | EM | |
| SSB | 0 | |
| LB | 0.30 | Hz |
| GB | 0 | |
| PC | 1.00 | |

C13CPD32 DMSO D:\\ abari 46

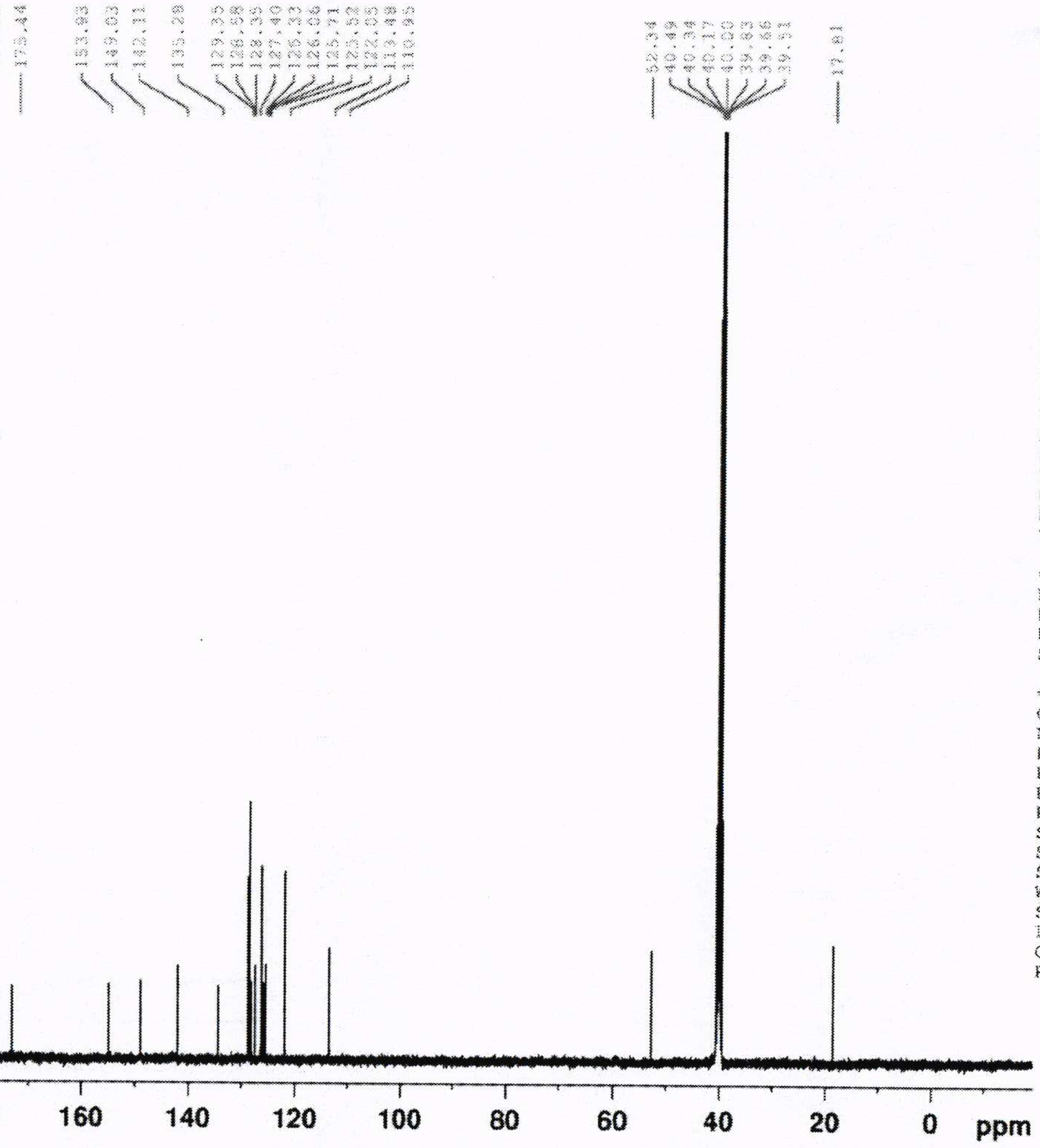


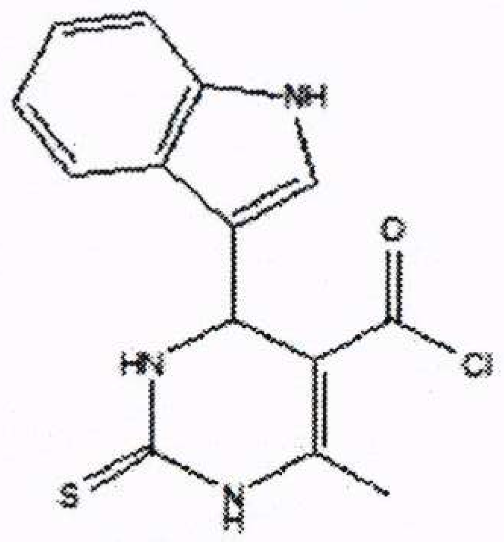


6a

BRUKER

| | |
|---|---|
| NAME | drshahat-8 |
| EXPNO | 11 |
| PROCNO | 1 |
| Date_ | 20210625 |
| Time | 20.47 |
| INSTRUM | spect |
| PROBHD | 5 mm PABBO BB- |
| PULPROG | zgpg30 |
| TD | 65536 |
| SOLVENT | DMSO |
| NS | 2000 |
| DS | 2 |
| SWH | 30030.029 Hz |
| FIDRES | 0.458222 Hz |
| AQ | 1.0912410 sec |
| RG | 14596.5 |
| DW | 16.650 usec |
| DE | 6.50 usec |
| TE | 299.1 K |
| D1 | 2.00000000 sec |
| D11 | 0.03000000 sec |
| TD0 | 1 |

======== CHANNEL f1 ========

| | |
|---|---|
| NUC1 | 13C |
| P1 | 20.00 usec |
| PL1 | -6.00 dB |
| SFO1 | 125.7703643 MHz |

======== CHANNEL f2 ========

| | |
|---|---|
| CPDPRG2 | waltz16 |
| NUC2 | 1H |
| PCPD2 | 80.00 usec |
| PL2 | -1.10 dB |
| PL12 | 13.44 dB |
| PL13 | 16.40 dB |
| SFO2 | 500.1320005 MHz |
| SI | 32768 |
| SF | 125.7577890 MHz |
| WDW | EM |
| SSB | 0 |
| LB | 1.00 Hz |
| GB | 0 |
| PC | 1.40 |

4-(2-Chloroquinolin-3-yl)-6-methyl-2-thioxo-1,2,3,4-tetrahydropyrimidine-5-carbonyl chloride 6b

ON DMSO D:\\ abar

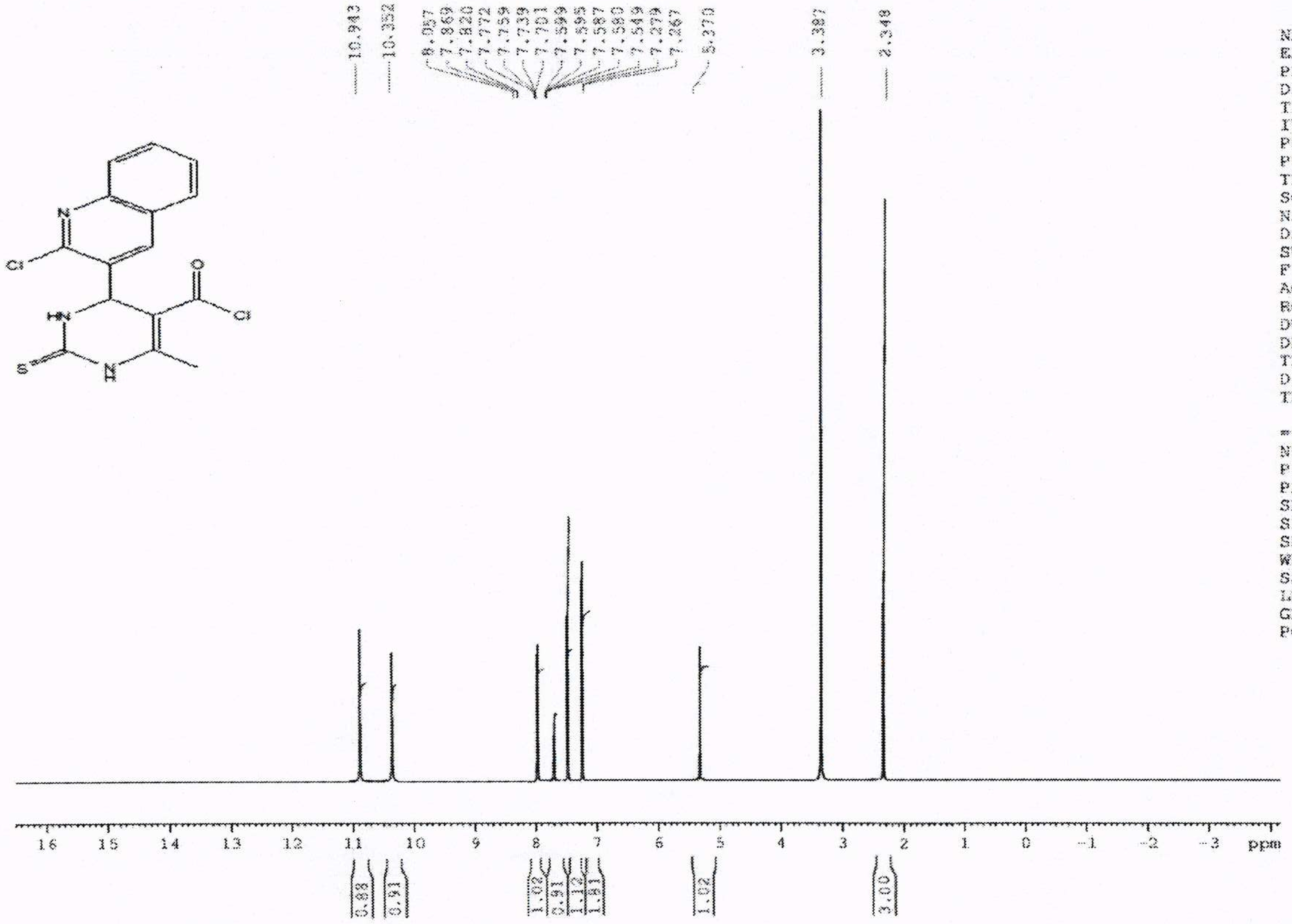


BRUKER

| | | |
|---|---|---|
| NAME | drshahat-7 | |
| EXPNO | 10 | |
| PROCNO | 1 | |
| Date_ | 20210624 | |
| Time | 18.59 | |
| INSTRUM | spect | |
| PROBHD | 5 mm PABBO BB- | |
| PULPROG | zg30 | |
| TD | 65536 | |
| SOLVENT | DMSO | |
| NS | 16 | |
| DS | 2 | |
| SWH | 10330.578 | Hz |
| FIDRES | 0.157632 | Hz |
| AQ | 3.1720407 | sec |
| RG | 90.5 | |
| DW | 48.400 | usec |
| DE | 6.50 | usec |
| TE | 298.3 | K |
| D1 | 1.00000000 | sec |
| TD0 | 1 | |
| ======== CHANNEL f1 ======== | | |
| NUC1 | 1H | |
| P1 | 14.70 | usec |
| PL1 | -1.10 | dB |
| SFO1 | 500.1330885 | MHz |
| SI | 32768 | |
| SF | 500.1300000 | MHz |
| WDW | EM | |
| SSB | 0 | |
| LB | 0.30 | Hz |
| GB | 0 | |
| PC | 1.00 | |

C13CPD32 DMSO D:\\ abari 45

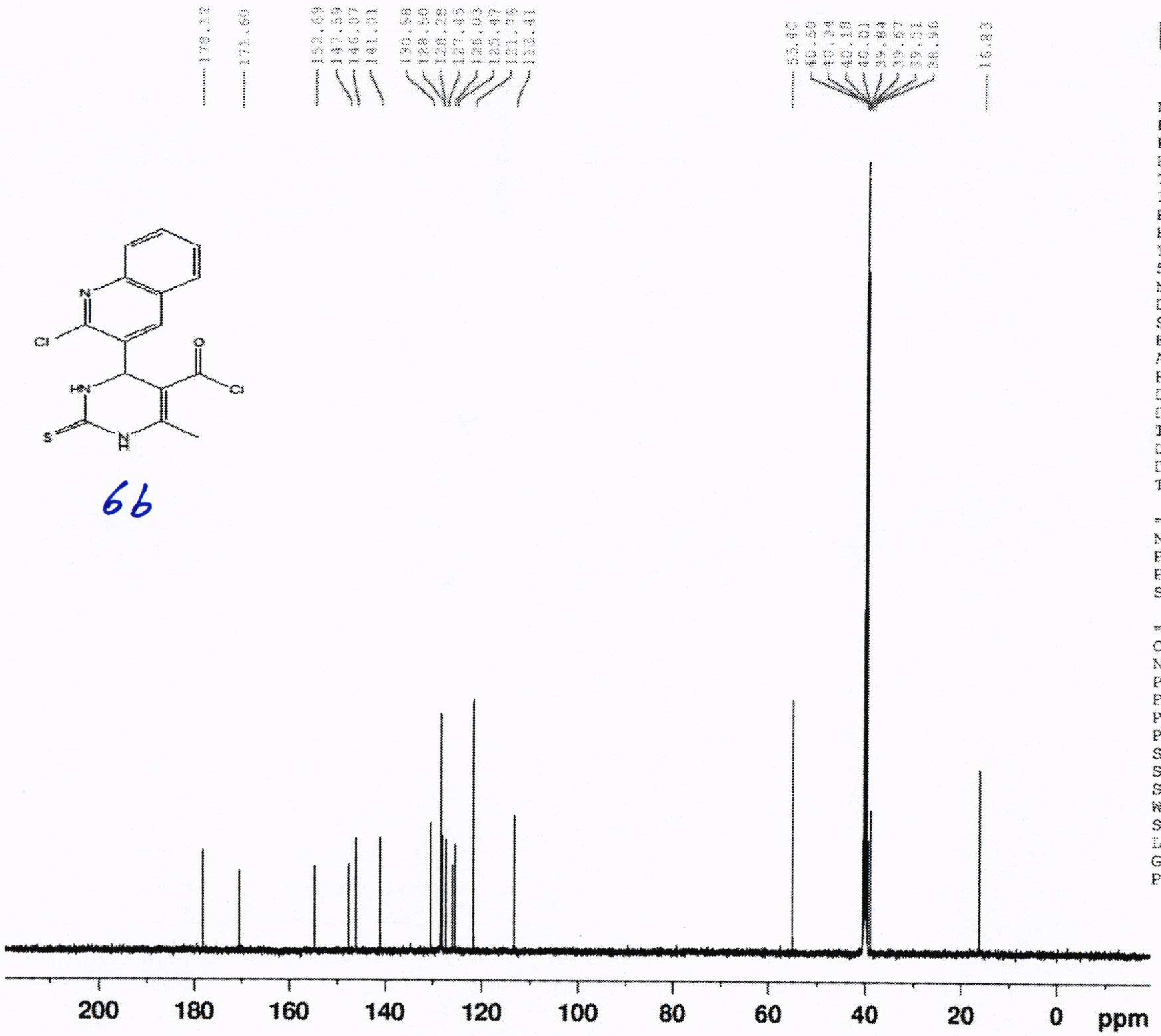


6b

BRUKER

| | |
|---|---|
| NAME | drshahat-7 |
| EXPNO | 11 |
| PROCNO | 1 |
| Date_ | 20210624 |
| Time | 13.45 |
| INSTRUM | spect |
| PROBHD | 5 mm PABBO BB- |
| PULPROG | zgpg30 |
| TD | 65536 |
| SOLVENT | DMSO |
| NS | 1104 |
| DS | 2 |
| SWH | 30030.029 Hz |
| FIDRES | 0.458222 Hz |
| AQ | 1.0912410 sec |
| RG | 2048 |
| DW | 16.650 usec |
| DE | 6.50 usec |
| TE | 299.0 K |
| D1 | 2.00000000 sec |
| D11 | 0.03000000 sec |
| TD0 | 1 |

======== CHANNEL f1 ========

| | |
|---|---|
| NUC1 | 13C |
| P1 | 20.00 usec |
| PL1 | -6.00 dB |
| SFO1 | 125.7703643 MHz |

======== CHANNEL f2 ========

| | |
|---|---|
| CPDPRG2 | waltz16 |
| NUC2 | 1H |
| PCPD2 | 80.00 usec |
| PL2 | -1.10 dB |
| PL12 | 13.44 dB |
| PL13 | 16.40 dB |
| SFO2 | 500.1320005 MHz |
| SI | 32768 |
| SF | 125.7577890 MHz |
| WDW | EM |
| SSB | 0 |
| LB | 1.00 Hz |
| GB | 0 |
| PC | 1.40 |

4-(1,3-Diphenyl-1H-pyrazol-4-yl)-6-methyl-2-thioxo-1,2,3,4-tetrahydropyrimidine-5-carbonyl chloride 6c

ON DMSO D:\\ abar

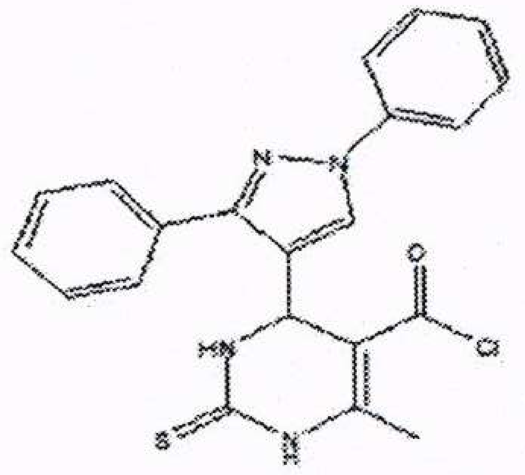


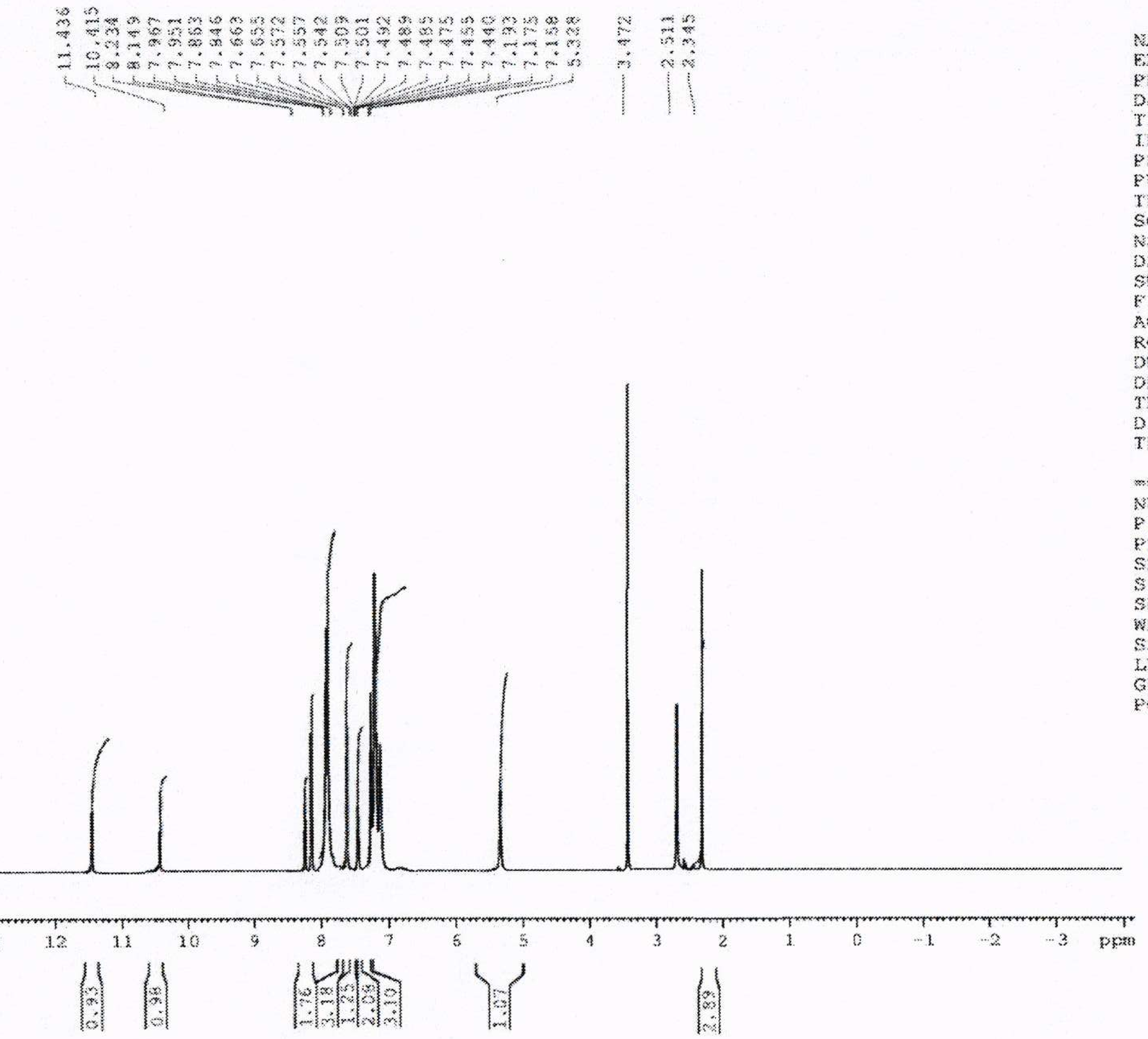


BRUKER

| | |
|---|---|
| NAME | drshahat-9 |
| EXPNO | 10 |
| PROCNO | 1 |
| Date_ | 20210622 |
| Time | 11.17 |
| INSTRUM | spect |
| PROBHD | 5 mm PABBO BB- |
| PULPROG | zg30 |
| TD | 65536 |
| SOLVENT | DMSO |
| NS | 16 |
| DS | 2 |
| SWH | 10330.578 Hz |
| FIDRES | 0.157632 Hz |
| AQ | 3.1720407 sec |
| RG | 128 |
| DW | 48.400 usec |
| DE | 6.50 usec |
| TE | 298.3 K |
| D1 | 1.00000000 sec |
| TD0 | 1 |

======== CHANNEL f1 ========

| | |
|---|---|
| NUC1 | 1H |
| P1 | 14.70 usec |
| PL1 | -1.10 dB |
| SFO1 | 500.1330885 MHz |
| SI | 32768 |
| SF | 500.1300000 MHz |
| WDW | EM |
| SSB | 0 |
| LB | 0.30 Hz |
| GB | 0 |
| PC | 1.00 |

C13CPD32 DMSO D:\\ abari 44

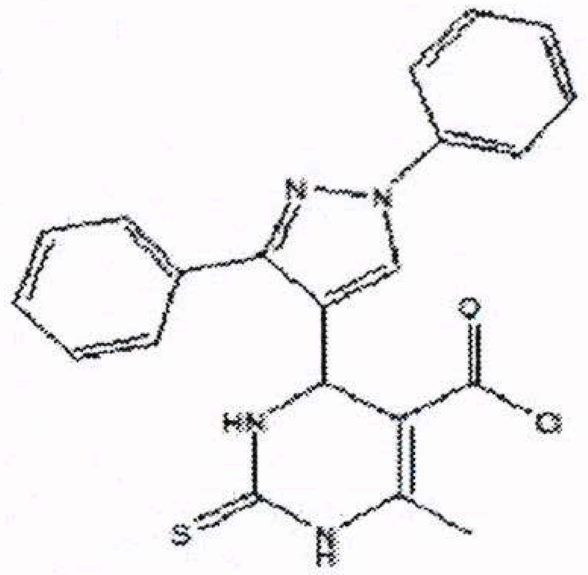


6c

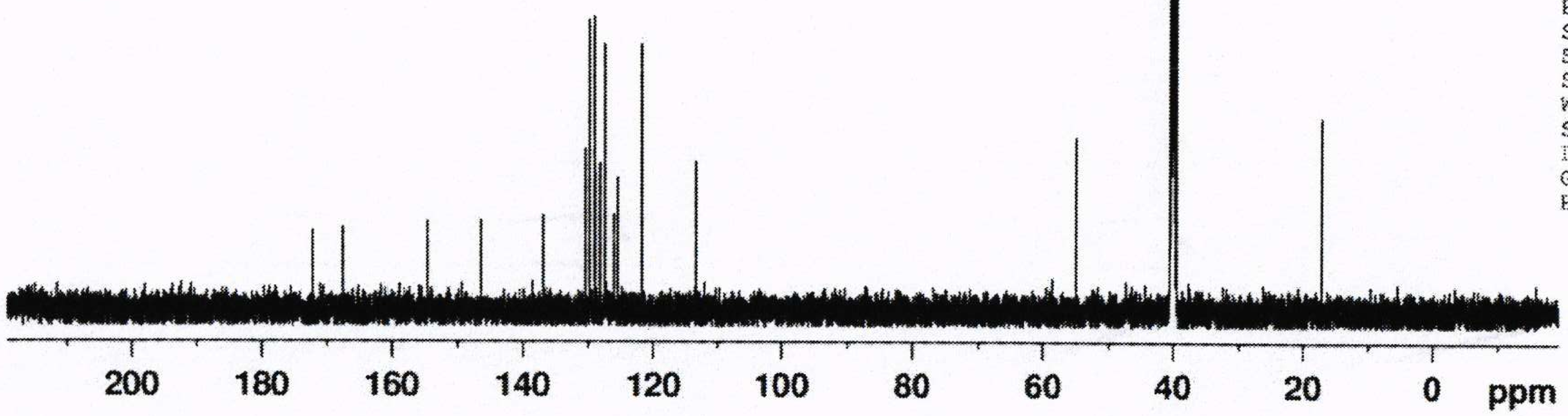


BRUKER

| | | |
|---|---|---|
| NAME | drshahat-9 | |
| EXPNO | 11 | |
| PROCNO | 1 | |
| Date_ | 20210622 | |
| Time | 12.07 | |
| INSTRUM | spect | |
| PROBHD | 5 mm PABBO BB- | |
| PULPROG | zgpg30 | |
| TD | 65536 | |
| SOLVENT | DMSO | |
| NS | 1165 | |
| DS | 2 | |
| SWH | 30030.029 | Hz |
| FIDRES | 0.458222 | Hz |
| AQ | 1.0912410 | sec |
| RG | 4597.6 | |
| DW | 16.650 | usec |
| DE | 6.50 | usec |
| TE | 299.2 | K |
| D1 | 2.00000000 | sec |
| D11 | 0.03000000 | sec |
| TD0 | 1 | |

======== CHANNEL f1 ========

| | | |
|---|---|---|
| NUC1 | 13C | |
| P1 | 20.00 | usec |
| PL1 | -6.00 | dB |
| SFO1 | 125.7703643 | MHz |

======== CHANNEL f2 ========

| | | |
|---|---|---|
| CPDPRG2 | waltz16 | |
| NUC2 | 1H | |
| PCPD2 | 80.00 | usec |
| PL2 | -1.10 | dB |
| PL12 | 13.44 | dB |
| PL13 | 16.40 | dB |
| SFO2 | 500.1320005 | MHz |
| SI | 32768 | |
| SF | 125.7577890 | MHz |
| WDW | EM | |
| SSB | 0 | |
| LB | 1.00 | Hz |
| GB | 0 | |
| PC | 1.40 | |

4-(1H-Indol-3-yl)-6-methyl-N-phenyl-2-thioxo-1,2,3,4-tetrahydropyrimidine-5-carboxamide

7a

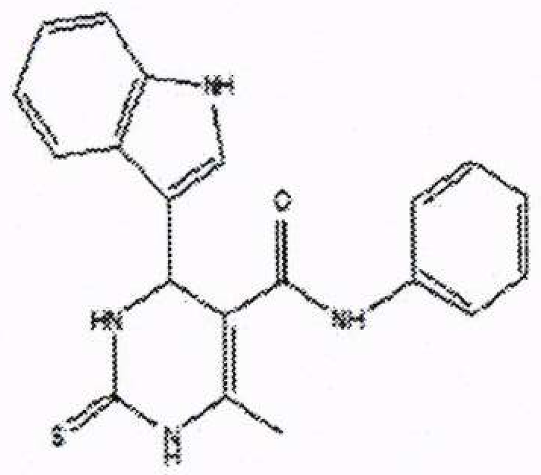


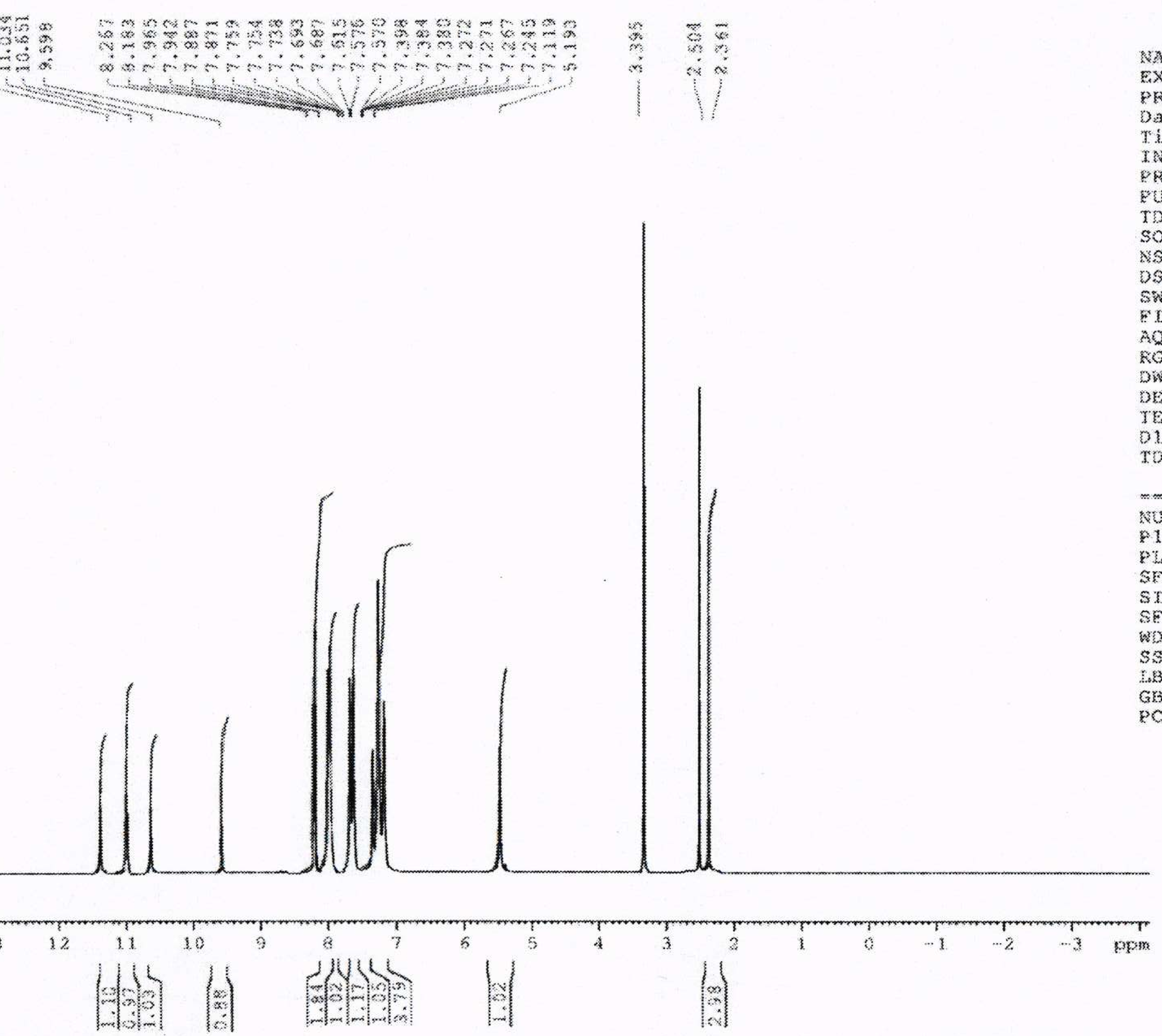


BRUKER

| | |
|---|---|
| NAME | drshahat-11 |
| EXPNO | 10 |
| PROCNO | 1 |
| Date_ | 20210624 |
| Time | 18.59 |
| INSTRUM | spect |
| PROBHD | 5 mm PABBO BB- |
| PULPROG | zg30 |
| TD | 65536 |
| SOLVENT | DMSO |
| NS | 16 |
| DS | 2 |
| SWH | 10330.578 Hz |
| FIDRES | 0.157632 Hz |
| AQ | 3.1720407 sec |
| RG | 90.5 |
| DW | 48.400 usec |
| DE | 6.50 usec |
| TE | 298.3 K |
| D1 | 1.00000000 sec |
| TD0 | 1 |

======== CHANNEL f1 ========

| | |
|---|---|
| NUC1 | 1H |
| P1 | 14.70 usec |
| PL1 | -1.10 dB |
| SFO1 | 500.1330885 MHz |
| SI | 32768 |
| SF | 500.1300000 MHz |
| WDW | EM |
| SSB | 0 |
| LB | 0.30 Hz |
| GB | 0 |
| PC | 1.00 |

C13CPD32 DMSO D:\\ abari 46

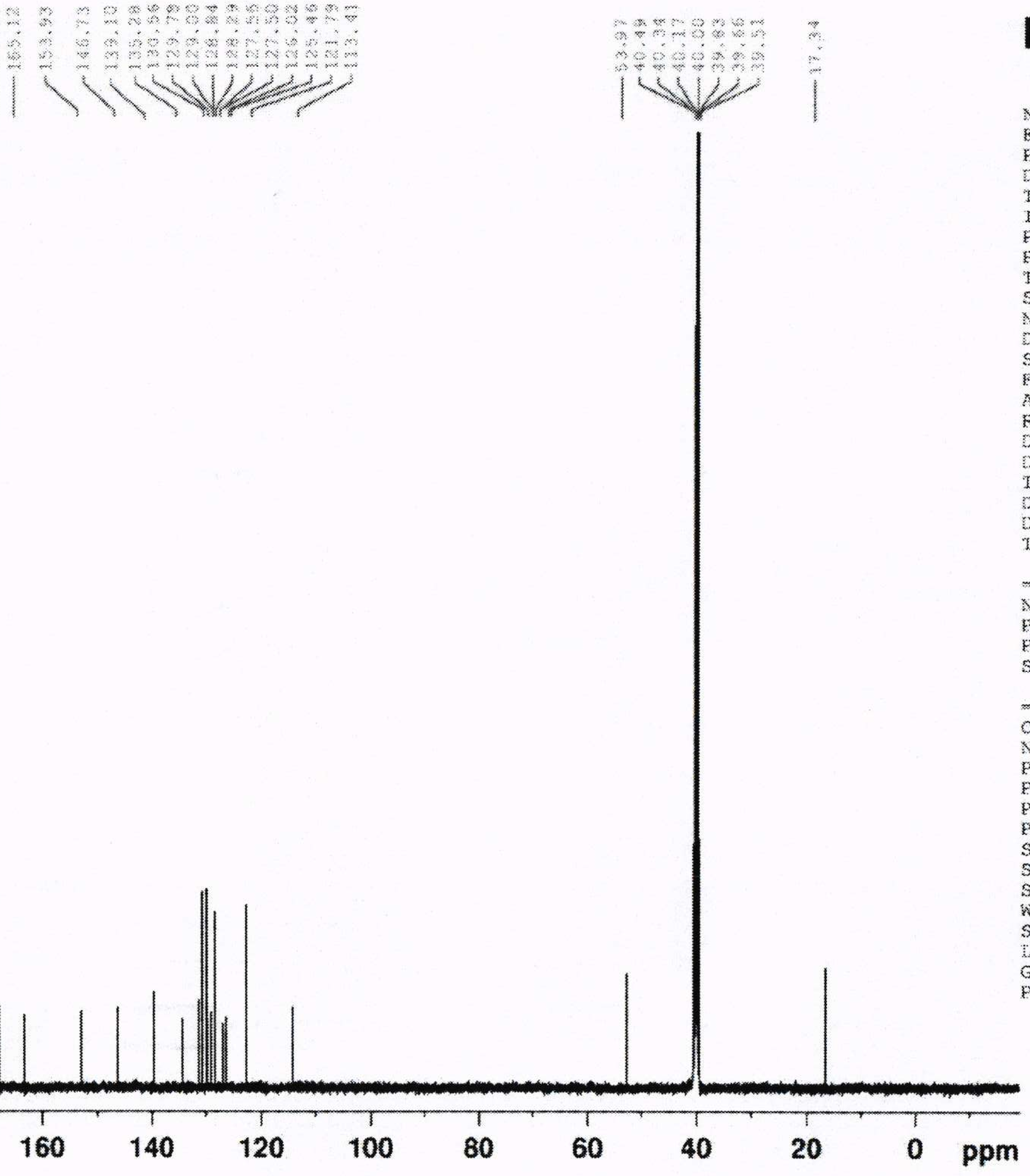


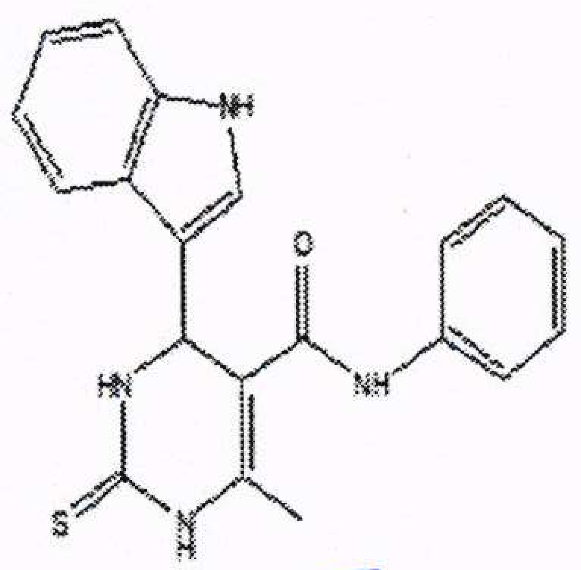


7a

BRUKER

| | | |
|---|---|---|
| NAME | drshahat-11 | |
| EXPNO | 11 | |
| PROCNO | 1 | |
| Date_ | 20210624 | |
| Time | 20.47 | |
| INSTRUM | spect | |
| PROBHD | 5 mm PABBO BB- | |
| PULPROG | zgpg30 | |
| TD | 65536 | |
| SOLVENT | DMSO | |
| NS | 2000 | |
| DS | 2 | |
| SWH | 30030.029 | Hz |
| FIDRES | 0.458222 | Hz |
| AQ | 1.0912410 | sec |
| RG | 14596.5 | |
| DW | 16.650 | usec |
| DE | 6.50 | usec |
| TE | 299.1 | K |
| D1 | 2.00000000 | sec |
| D11 | 0.03000000 | sec |
| TD0 | 1 | |

======== CHANNEL f1 ========

| | | |
|---|---|---|
| NUC1 | 13C | |
| P1 | 20.00 | usec |
| PL1 | -6.00 | dB |
| SFO1 | 125.7703643 | MHz |

======== CHANNEL f2 ========

| | | |
|---|---|---|
| CPDPRG2 | waltz16 | |
| NUC2 | 1H | |
| PCPD2 | 80.00 | usec |
| PL2 | -1.10 | dB |
| PL12 | 13.44 | dB |
| PL13 | 16.40 | dB |
| SFO2 | 500.1320005 | MHz |
| SI | 32768 | |
| SF | 125.7577890 | MHz |
| WDW | EM | |
| SSB | 0 | |
| LB | 1.00 | Hz |
| GB | 0 | |
| PC | 1.40 | |

4-(2-Chloroquinolin-3-yl)-6-methyl-N-phenyl-2-thioxo-1,2,3,4-tetrahydropyrimidine-5-carboxamide 7b

ƆN DMSO D:\\ abar

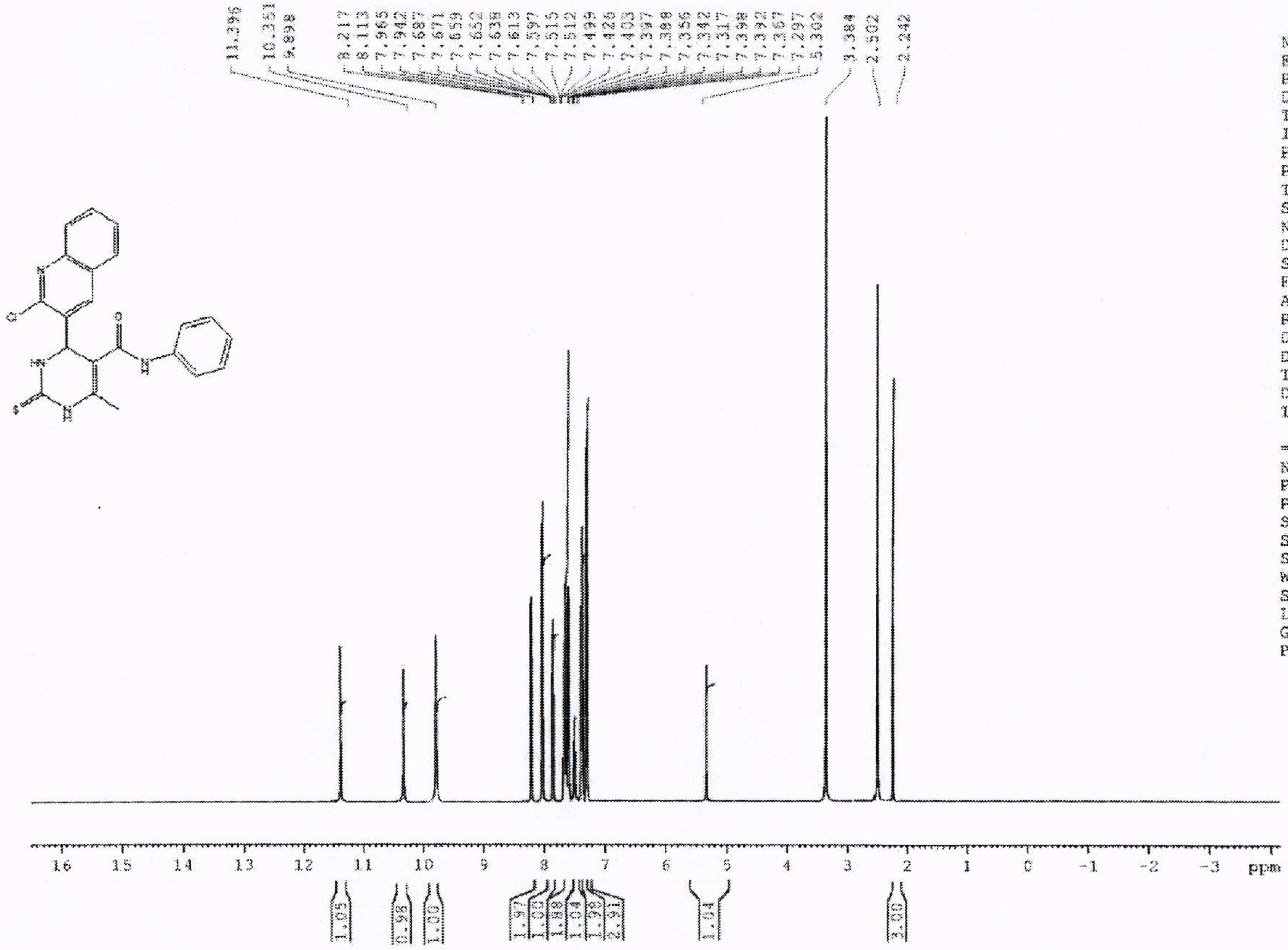


BRUKER

| | |
|---|---|
| NAME | drshahat-10 |
| EXPNO | 10 |
| PROCNO | 1 |
| Date_ | 20210624 |
| Time | 18.59 |
| INSTRUM | spect |
| PROBHD | 5 mm PABBO BB- |
| PULPROG | zg30 |
| TD | 65536 |
| SOLVENT | DMSO |
| NS | 16 |
| DS | 2 |
| SWH | 10330.578 Hz |
| FIDRES | 0.157632 Hz |
| AQ | 3.1720407 sec |
| RG | 90.5 |
| DW | 48.400 usec |
| DE | 6.50 usec |
| TE | 298.3 K |
| D1 | 1.00000000 sec |
| TD0 | 1 |

======== CHANNEL f1 ========

| | |
|---|---|
| NUC1 | 1H |
| P1 | 14.70 usec |
| PL1 | -1.10 dB |
| SFO1 | 500.1330885 MHz |
| SI | 32768 |
| SF | 500.1300000 MHz |
| WDW | EM |
| SSB | 0 |
| LB | 0.30 Hz |
| GB | 0 |
| PC | 1.00 |

C13CPD32 DMSO D:\\ abari 46

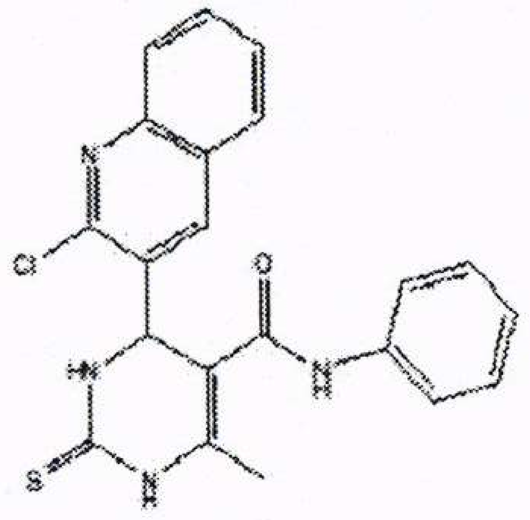


76

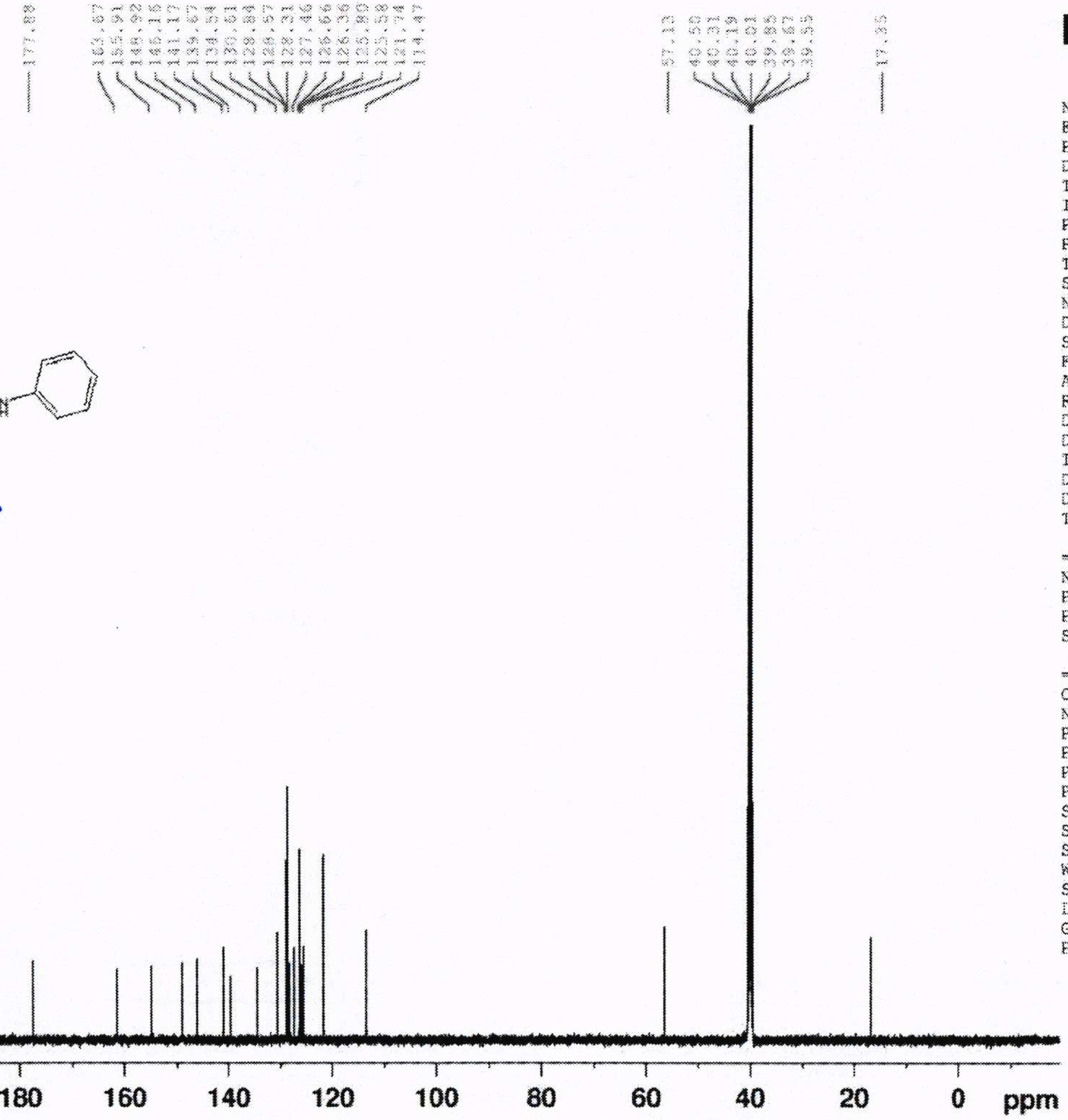


BRUKER

| | | |
|---|---|---|
| NAME | drshahat-10 | |
| EXPNO | 11 | |
| PROCNO | 1 | |
| Date_ | 20210624 | |
| Time | 20.47 | |
| INSTRUM | spect | |
| PROBHD | 5 mm PABBO BB- | |
| PULPROG | zgpg30 | |
| TD | 65536 | |
| SOLVENT | DMSO | |
| NS | 2000 | |
| DS | 2 | |
| SWH | 30030.029 | Hz |
| FIDRES | 0.458222 | Hz |
| AQ | 1.0912410 | sec |
| RG | 14596.5 | |
| DW | 16.650 | usec |
| DE | 6.50 | usec |
| TE | 299.1 | K |
| D1 | 2.00000000 | sec |
| D11 | 0.03000000 | sec |
| TD0 | 1 | |
| ======== CHANNEL f1 ======== | | |
| NUC1 | 13C | |
| P1 | 20.00 | usec |
| PL1 | -6.00 | dB |
| SFO1 | 125.7703643 | MHz |
| ======== CHANNEL f2 ======== | | |
| CPDPRG2 | waltz16 | |
| NUC2 | 1H | |
| PCPD2 | 80.00 | usec |
| PL2 | -1.10 | dB |
| PL12 | 13.44 | dB |
| PL13 | 16.40 | dB |
| SFO2 | 500.1320005 | MHz |
| SI | 32768 | |
| SF | 125.7577890 | MHz |
| WDW | EM | |
| SSB | 0 | |
| LB | 1.00 | Hz |
| GB | 0 | |
| PC | 1.40 | |

**Cairo University**
**Micro Analytical Center**

***DI Analysis***
***Shimadzu Qp-2010 Plus***

Sample Information

| | |
|---|---|
| Analyzed by | : Mai Younis |
| Analyzed | : 22/06/2021 02:44:23  م |
| Sample Name | : 10 |
| Sample ID | : |
| Customer Name | : Dr. Moh F |
| Data File | : C:\GCMSsolution\Data\Project1\21.QGD |
| Org Data File | : C:\GCMSsolution\Data\Project1\21.QGD |
| Method File | : C:\GCMSsolution\Data\Project1\Mai.qgm |
| Org Method File | : C:\GCMSsolution\Data\Project1\Mai.qgm |
| Report File | : |
| Tuning File | : C:\GCMSsolution\System\Tune1_default1.qgt |
| $EndIf$Modified by | : Mai Younis |
| Modified | : 22/06/2021 02:49:22 م |

Method

------ Analytical Line 1 ------

| | |
|---|---|
| IonSourceTemp | :250.00 °C |
| [MS Table] | |
| --Group 1 - Event 1-- | |
| Start Time | :0.00min |
| End Time | :10.00min |
| ACQ Mode | :Scan |
| Event Time | :0.50sec |
| Scan Speed | :1000 |
| Start m/z | :50.00 |
| End m/z | :500.00 |
| Electron Voltage | : 70 eV |
| Ionization Mode | : EI |

C:\GCMSsolution\Data\Project1\21.QGD

Chromatogram 21 C:\GCMSsolution\Data\Project1\21.QGD

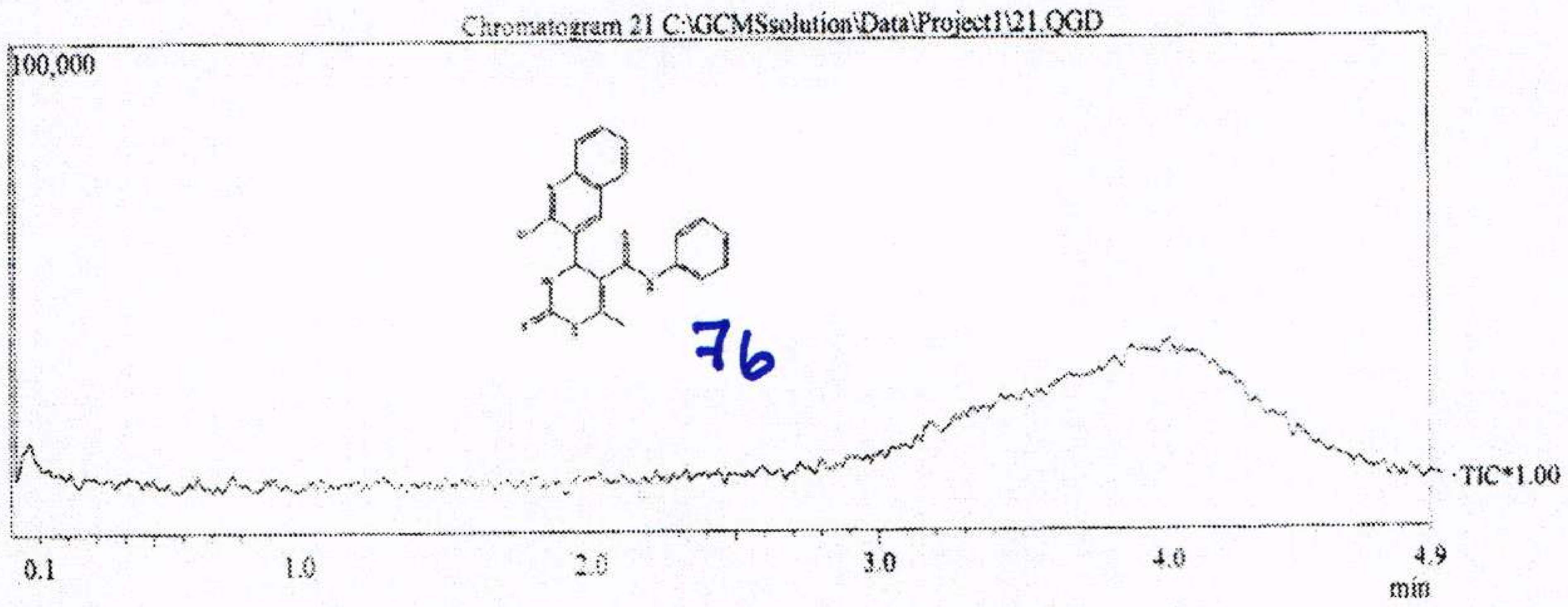


76

Spectrum

Line#:1 R.Time:3.1(Scan#:370)
MassPeaks:408
RawMode:Single 3.1(370) BasePeak:92(537)
BG Mode:None Group 1 - Event 1

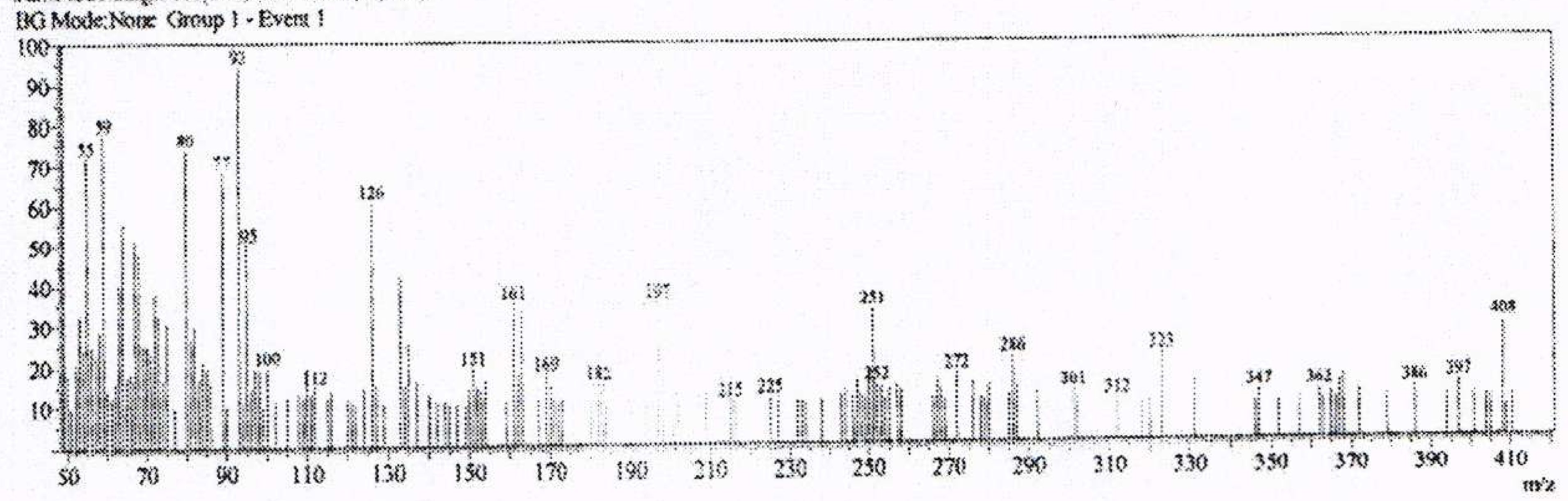


Mass Table
Line#:1 R.Time:3.1(Scan#:370)
MassPeaks:408
RawMode:Single 3.1(370) BasePeak:92(537)
BG Mode:None Group 1 - Event 1

| # | m/z | Abs. In | Rel. Int. | # | m/z | Abs. In | Rel. Int. | # | m/z | Abs. In | Rel. Int. |
|---|---|---|---|---|---|---|---|---|---|---|---|
| 1 | 50.00 | 97 | 18.06 | 4 | 53.00 | 177 | 32.96 | 7 | 56.00 | 135 | 25.14 |
| 2 | 51.00 | 52 | 9.68 | 5 | 54.00 | 127 | 23.65 | 8 | 57.00 | 122 | 22.72 |
| 3 | 52.00 | 110 | 20.48 | 6 | 55.00 | 386 | 71.88 | 9 | 58.00 | 154 | 28.68 |

4-(1,3-Diphenyl-1H-pyrazol-4-yl)-6-methyl-N-phenyl-2-thioxo-1,2,3,4-tetrahydropyrimidine-5-carboxamide 7c

ON DMSO D:\\ abar

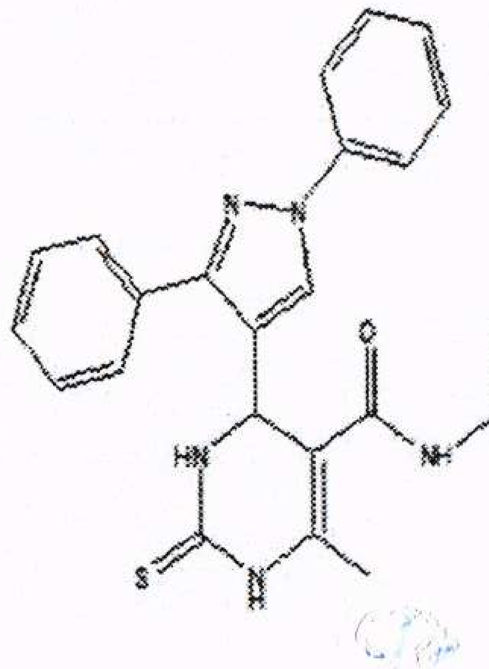


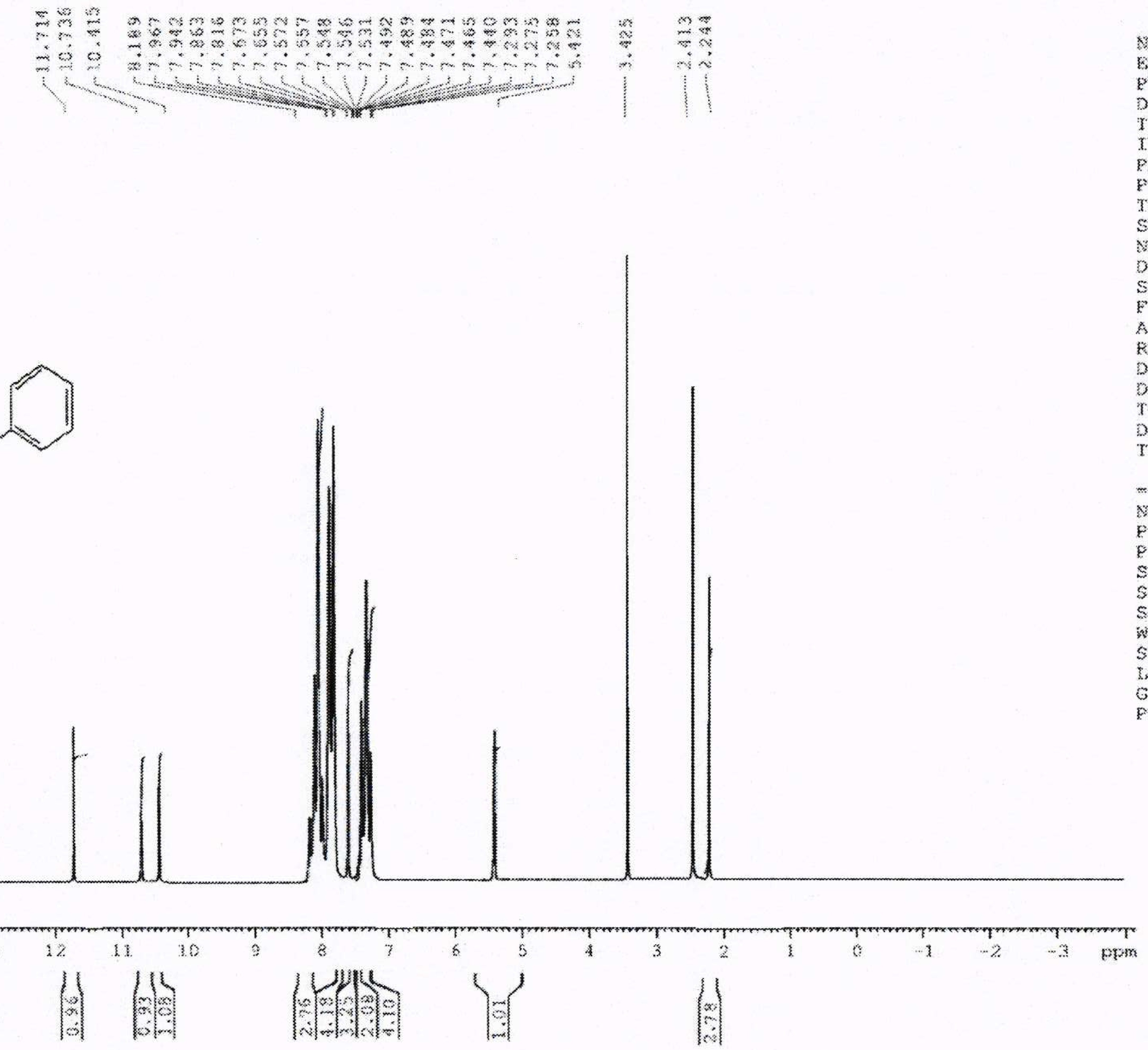


BRUKER

| | |
|---|---|
| NAME | drshahat-12 |
| EXPNO | 10 |
| PROCNO | 1 |
| Date_ | 20210624 |
| Time | 11.17 |
| INSTRUM | spect |
| PROBHD | 5 mm PABBO BB- |
| PULPROG | zg30 |
| TD | 65536 |
| SOLVENT | DMSO |
| NS | 16 |
| DS | 2 |
| SWH | 10330.578 Hz |
| FIDRES | 0.157632 Hz |
| AQ | 3.1720407 sec |
| RG | 128 |
| DW | 48.400 usec |
| DE | 6.50 usec |
| TE | 298.3 K |
| D1 | 1.00000000 sec |
| TD0 | 1 |

======== CHANNEL f1 ========

| | |
|---|---|
| NUC1 | 1H |
| P1 | 14.70 usec |
| PL1 | -1.10 dB |
| SFO1 | 500.1330885 MHz |
| SI | 32768 |
| SF | 500.1300000 MHz |
| WDW | EM |
| SSB | 0 |
| LB | 0.30 Hz |
| GB | 0 |
| PC | 1.00 |

C13CPD32 DMSO D:\\ abari 44

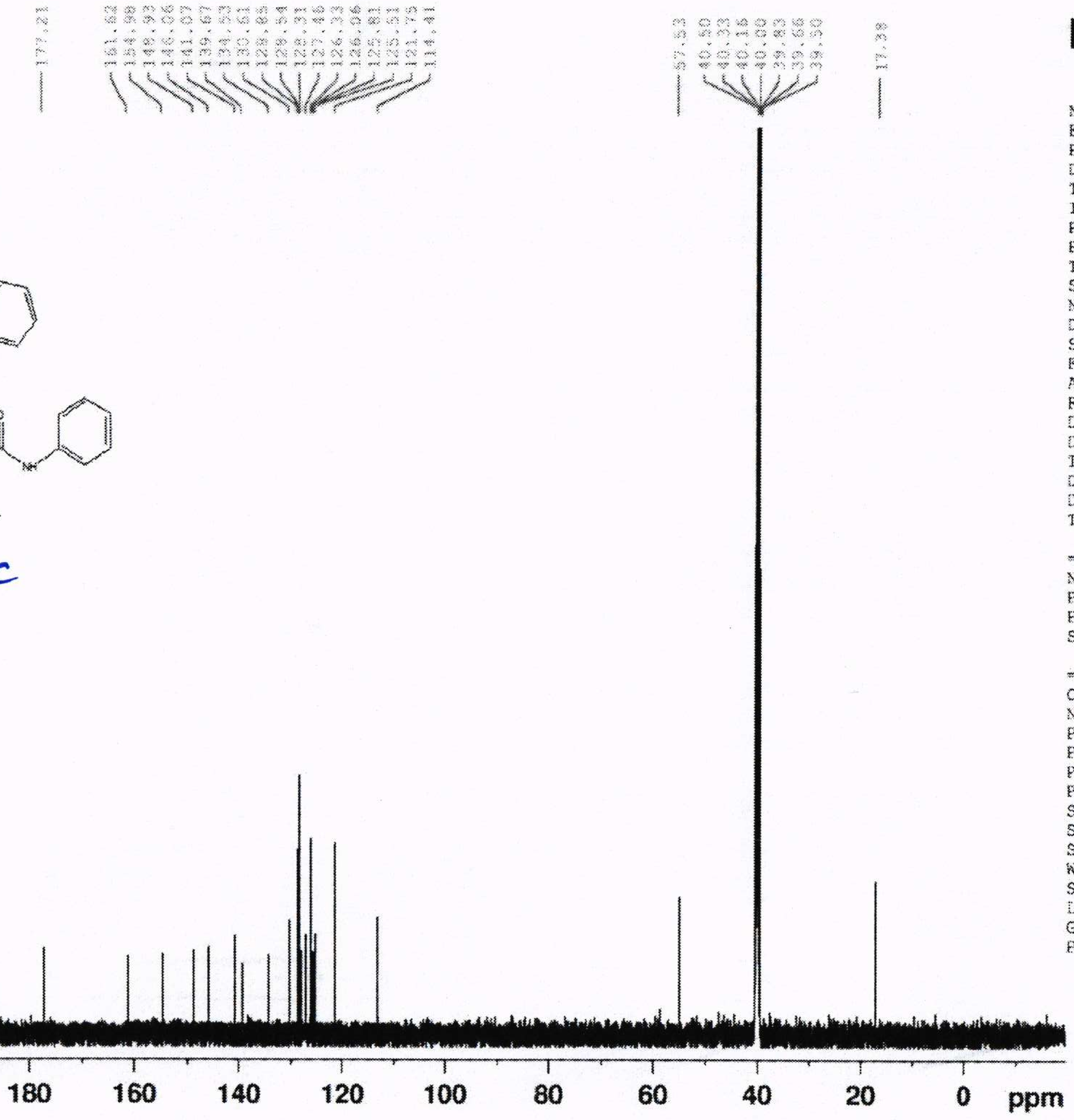


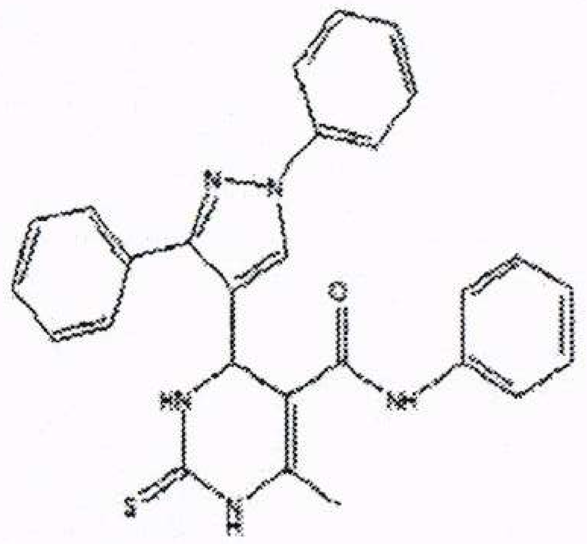


7c

| | | |
|---|---|---|
| NAME | drshahat-12 | |
| EXPNO | 11 | |
| PROCNO | 1 | |
| Date_ | 20210624 | |
| Time | 12.07 | |
| INSTRUM | spect | |
| PROBHD | 5 mm PABBO BB- | |
| PULPROG | zgpg30 | |
| TD | 65536 | |
| SOLVENT | DMSO | |
| NS | 1165 | |
| DS | 2 | |
| SWH | 30030.029 | Hz |
| FIDRES | 0.458222 | Hz |
| AQ | 1.0912410 | sec |
| RG | 4597.6 | |
| DW | 16.650 | usec |
| DE | 6.50 | usec |
| TE | 299.2 | K |
| D1 | 2.00000000 | sec |
| D11 | 0.03000000 | sec |
| TD0 | 1 | |

======== CHANNEL f1 ========

| | | |
|---|---|---|
| NUC1 | 13C | |
| P1 | 20.00 | usec |
| PL1 | -6.00 | dB |
| SFO1 | 125.7703643 | MHz |

======== CHANNEL f2 ========

| | | |
|---|---|---|
| CPDPRG2 | waltz16 | |
| NUC2 | 1H | |
| PCPD2 | 80.00 | usec |
| PL2 | -1.10 | dB |
| PL12 | 13.44 | dB |
| PL13 | 16.40 | dB |
| SFO2 | 500.1320005 | MHz |
| SI | 32768 | |
| SF | 125.7577890 | MHz |
| WDW | EM | |
| SSB | 0 | |
| LB | 1.00 | Hz |
| GB | 0 | |
| PC | 1.40 | |

4-(1H-Indol-3-yl)-6-methyl-2-thioxo-N-p-tolyl-1,2,3,4-tetrahydropyrimidine-5-carboxamide

8a

DN DMSO D:\\ abar

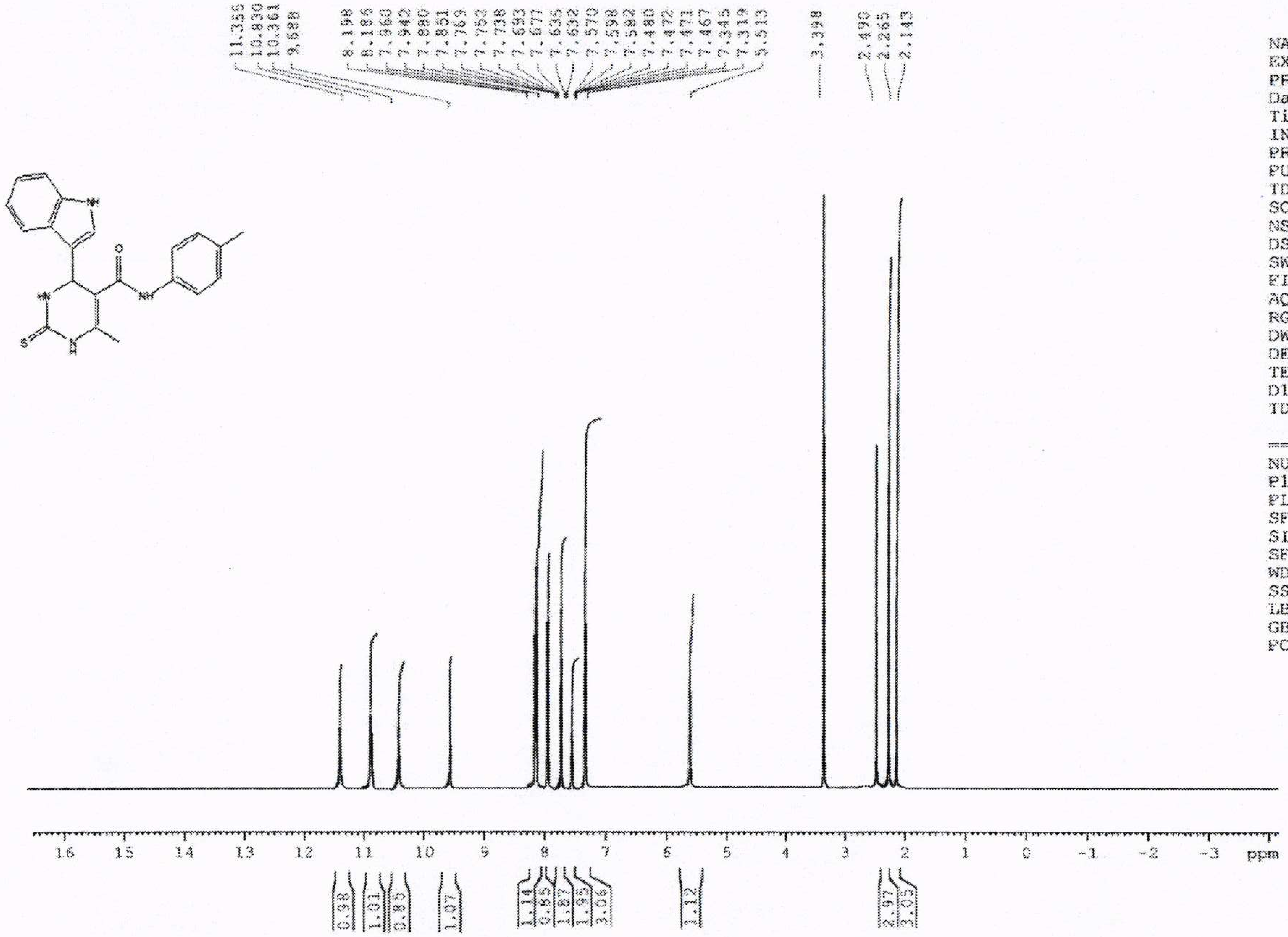


| | |
|---|---|
| NAME | drshahat-14 |
| EXPNO | 10 |
| PROCNO | 1 |
| Date_ | 20210624 |
| Time | 18.59 |
| INSTRUM | spect |
| PROBHD | 5 mm PABBO BB- |
| PULPROG | zg30 |
| TD | 65536 |
| SOLVENT | DMSO |
| NS | 16 |
| DS | 2 |
| SWH | 10330.578 Hz |
| FIDRES | 0.157632 Hz |
| AQ | 3.1720407 sec |
| RG | 90.5 |
| DW | 48.400 usec |
| DE | 6.50 usec |
| TE | 298.3 K |
| D1 | 1.00000000 sec |
| TD0 | 1 |

======== CHANNEL f1 ========

| | |
|---|---|
| NUC1 | 1H |
| P1 | 14.70 usec |
| PL1 | -1.10 dB |
| SFO1 | 500.1330885 MHz |
| SI | 32768 |
| SF | 500.1300000 MHz |
| WDW | EM |
| SSB | 0 |
| LB | 0.30 Hz |
| GB | 0 |
| PC | 1.00 |

C13CPD32 DMSO D:\\ abari 46

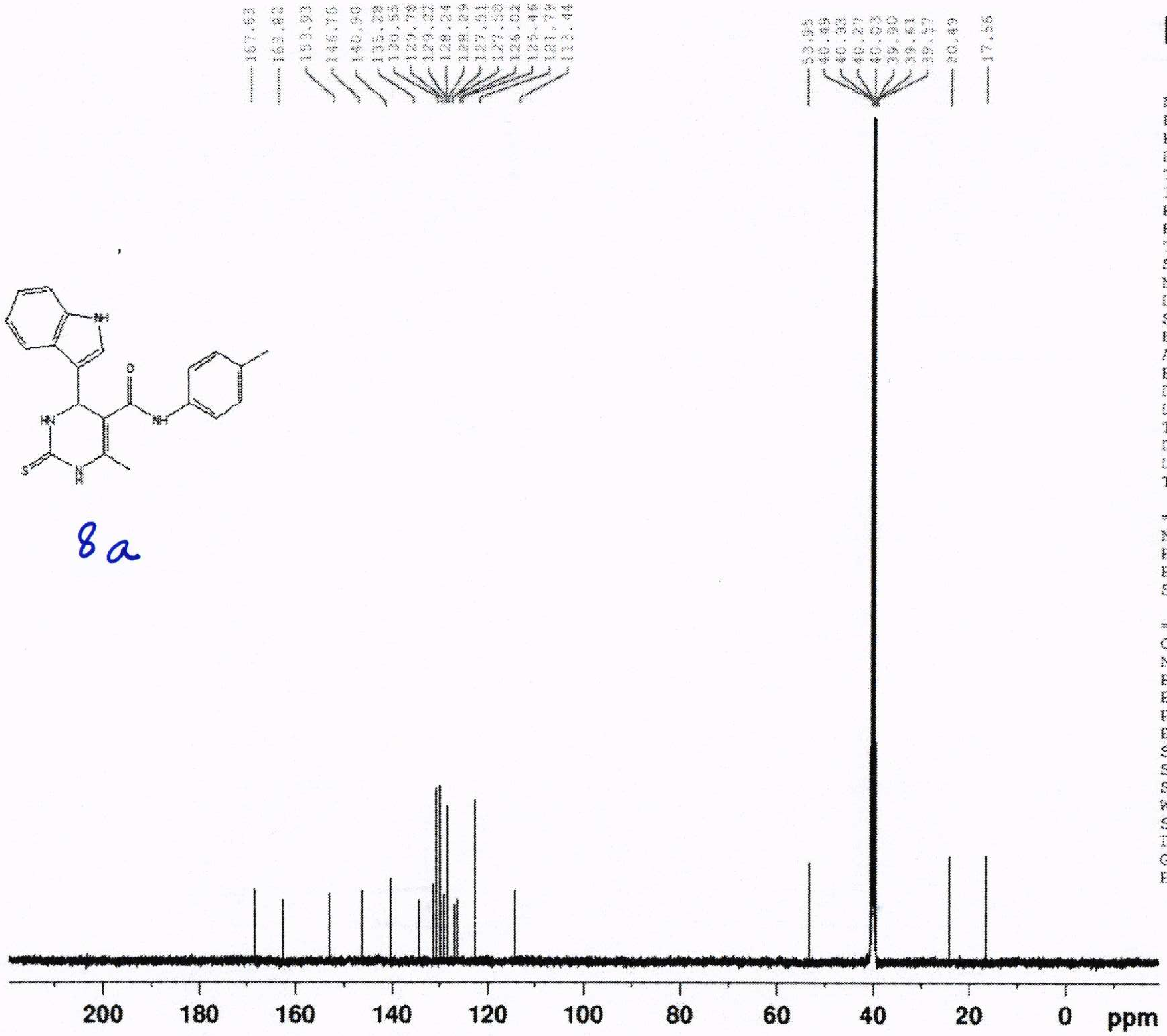


8a

BRUKER

| | |
|---|---|
| NAME | drshahat-14 |
| EXPNO | 11 |
| PROCNO | 1 |
| Date_ | 20210624 |
| Time | 20.47 |
| INSTRUM | spect |
| PROBHD | 5 mm PABBO BB- |
| PULPROG | zgpg30 |
| TD | 65536 |
| SOLVENT | DMSO |
| NS | 2000 |
| DS | 2 |
| SWH | 30030.029 Hz |
| FIDRES | 0.458222 Hz |
| AQ | 1.0912410 sec |
| RG | 14596.5 |
| DW | 16.650 usec |
| DE | 6.50 usec |
| TE | 299.1 K |
| D1 | 2.00000000 sec |
| D11 | 0.03000000 sec |
| TD0 | 1 |

======== CHANNEL f1 ========

| | |
|---|---|
| NUC1 | 13C |
| P1 | 20.00 usec |
| PL1 | -6.00 dB |
| SFO1 | 125.7703643 MHz |

======== CHANNEL f2 ========

| | |
|---|---|
| CPDPRG2 | waltz16 |
| NUC2 | 1H |
| PCPD2 | 80.00 usec |
| PL2 | -1.10 dB |
| PL12 | 13.44 dB |
| PL13 | 16.40 dB |
| SFO2 | 500.1320005 MHz |
| SI | 32768 |
| SF | 125.7577890 MHz |
| WDW | EM |
| SSB | 0 |
| LB | 1.00 Hz |
| GB | 0 |
| PC | 1.40 |

4-(2-Chloroquinolin-3-yl)-6-methyl-2-thioxo-N-p-tolyl-1,2,3,4-tetrahydropyrimidine-5-carboxamide

8b

ON DMSO D:\\ abar

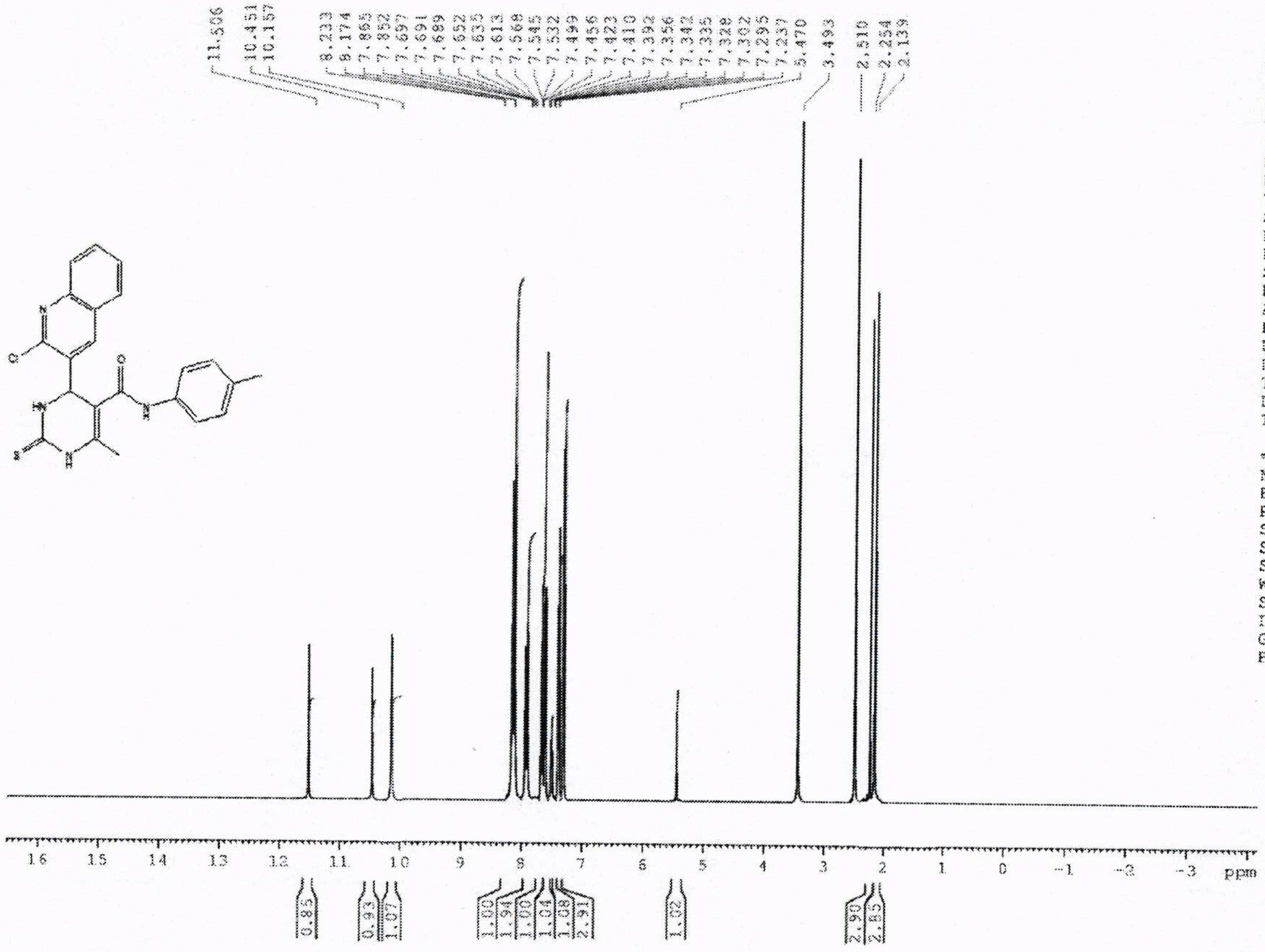


BRUKER

| | |
|---|---|
| NAME | drshahat-13 |
| EXPNO | 10 |
| PROCNO | 1 |
| Date_ | 20210623 |
| Time | 18.59 |
| INSTRUM | spect |
| PROBHD | 5 mm PABBO BB- |
| PULPROG | zg30 |
| TD | 65536 |
| SOLVENT | DMSO |
| NS | 16 |
| DS | 2 |
| SWH | 10330.578 Hz |
| FIDRES | 0.157632 Hz |
| AQ | 3.1720407 sec |
| RG | 90.5 |
| DW | 48.400 usec |
| DE | 6.50 usec |
| TE | 298.3 K |
| D1 | 1.00000000 sec |
| TD0 | 1 |

======== CHANNEL f1 ========

| | |
|---|---|
| NUC1 | 1H |
| P1 | 14.70 usec |
| PL1 | -1.10 dB |
| SFO1 | 500.1330885 MHz |
| SI | 32768 |
| SF | 500.1300000 MHz |
| WDW | EM |
| SSB | 0 |
| LB | 0.30 Hz |
| GB | 0 |
| PC | 1.00 |

C13CPD32 DMSO D:\\ abari 46

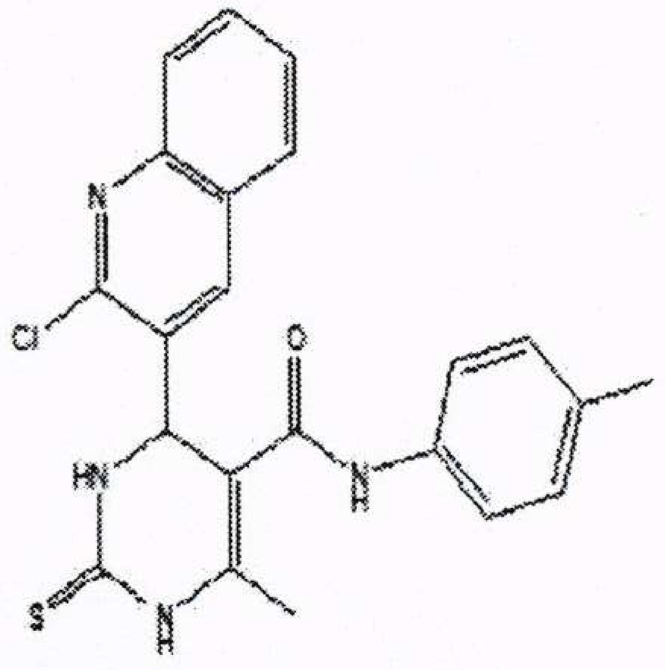

8b

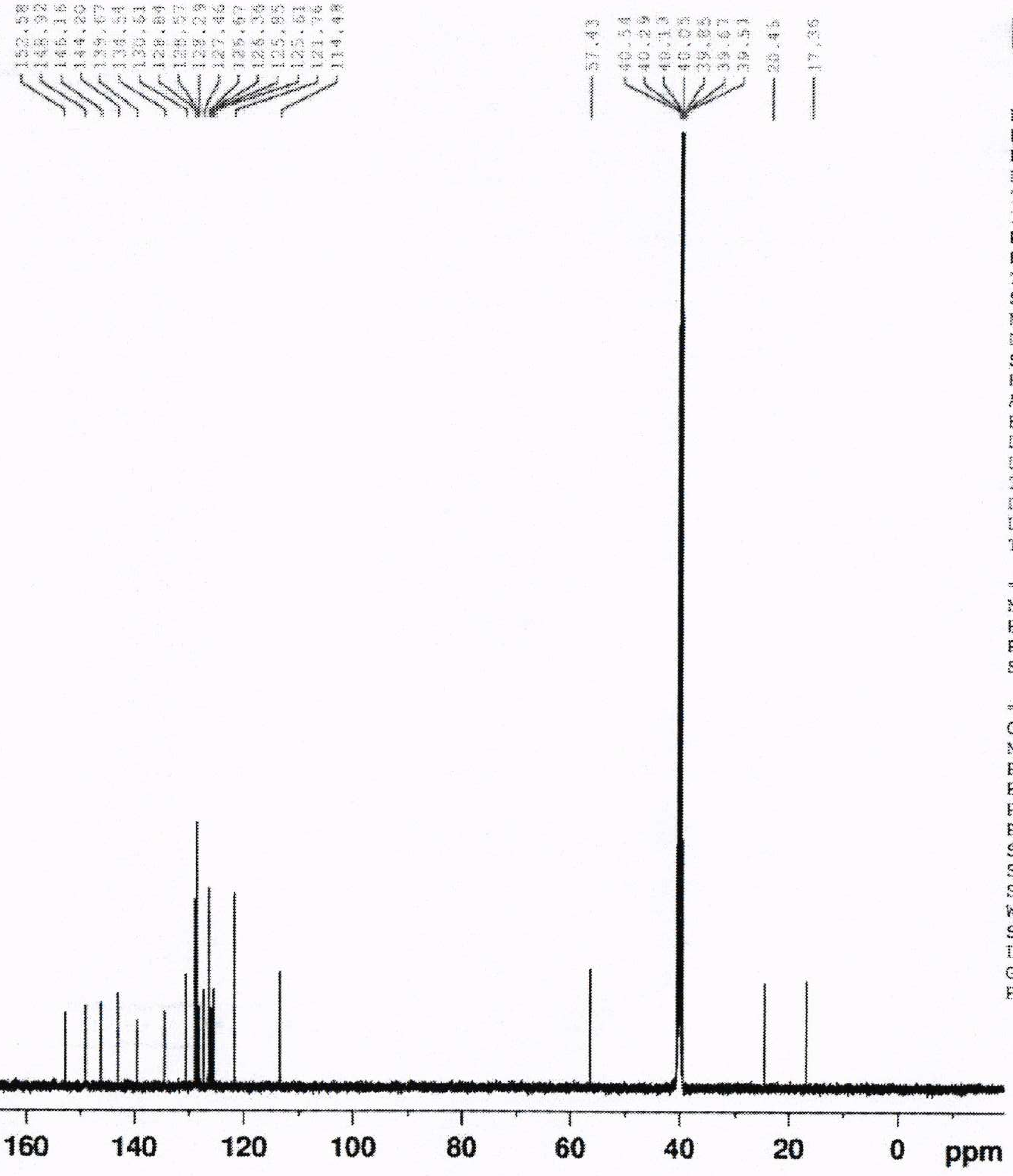


BRUKER

| | | |
|---|---|---|
| NAME | drshahat-13 | |
| EXPNO | 11 | |
| PROCNO | 1 | |
| Date_ | 20210623 | |
| Time | 20.47 | |
| INSTRUM | spect | |
| PROBHD | 5 mm PABBO BB- | |
| PULPROG | zgpg30 | |
| TD | 65536 | |
| SOLVENT | DMSO | |
| NS | 2000 | |
| DS | 2 | |
| SWH | 30030.029 | Hz |
| FIDRES | 0.458222 | Hz |
| AQ | 1.0912410 | sec |
| RG | 14596.5 | |
| DW | 16.650 | usec |
| DE | 6.50 | usec |
| TE | 299.1 | K |
| D1 | 2.00000000 | sec |
| D11 | 0.03000000 | sec |
| TD0 | 1 | |

======== CHANNEL f1 ========

| | | |
|---|---|---|
| NUC1 | 13C | |
| P1 | 20.00 | usec |
| PL1 | -6.00 | dB |
| SFO1 | 125.7703643 | MHz |

======== CHANNEL f2 ========

| | | |
|---|---|---|
| CPDPRG2 | waltz16 | |
| NUC2 | 1H | |
| PCPD2 | 80.00 | usec |
| PL2 | -1.10 | dB |
| PL12 | 13.44 | dB |
| PL13 | 16.40 | dB |
| SFO2 | 500.1320005 | MHz |
| SI | 32768 | |
| SF | 125.7577890 | MHz |
| WDW | EM | |
| SSB | 0 | |
| LB | 1.00 | Hz |
| GB | 0 | |
| PC | 1.40 | |

4-(1,3-Diphenyl-1H-pyrazol-4-yl)-6-methyl-2-thioxo-N-p-tolyl-1,2,3,4-tetrahydropyrimidine-5-carboxamide 8c

ON DMSO D:\\ abar

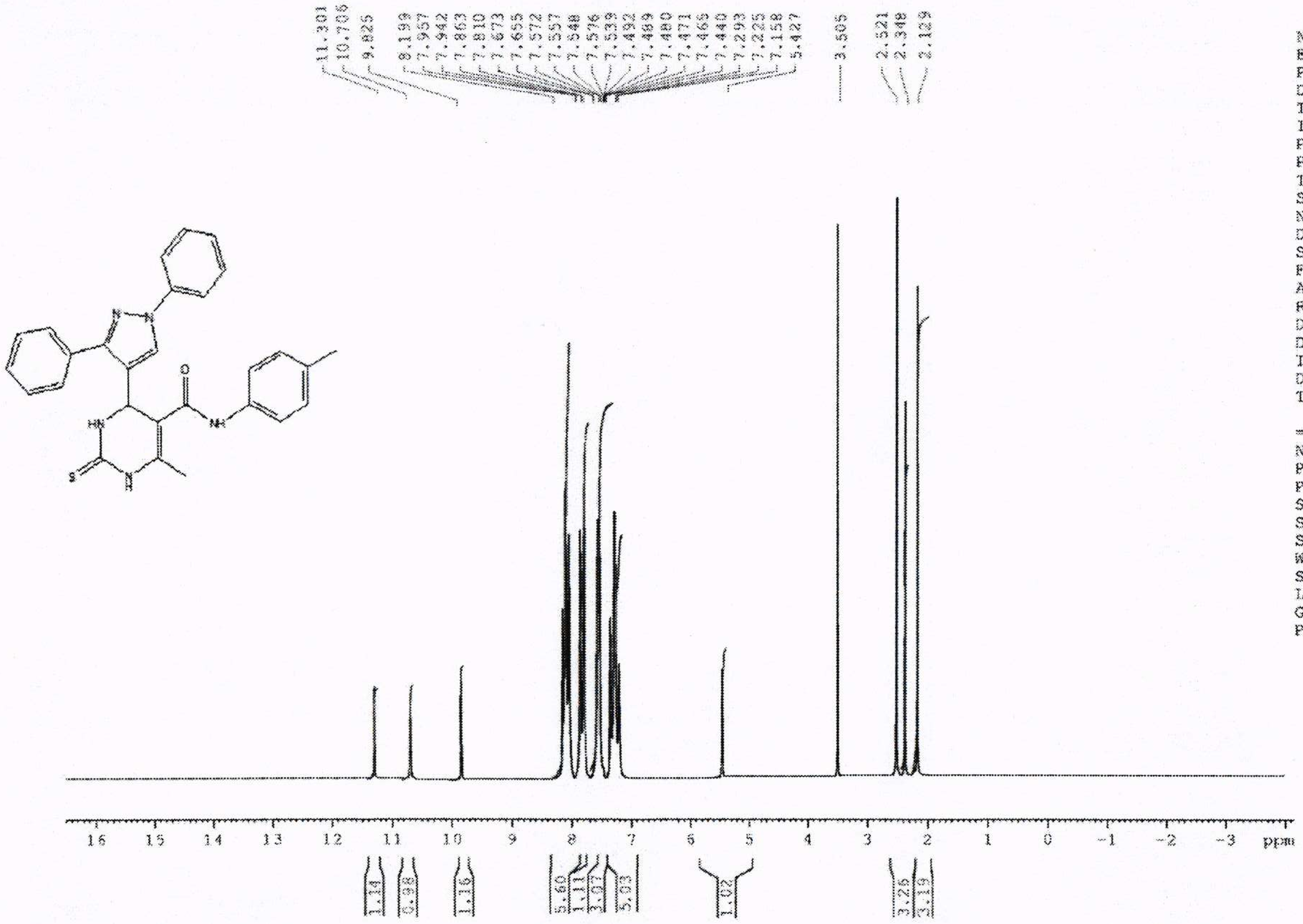


BRUKER

| | | |
|---|---|---|
| NAME | drshahat-15 | |
| EXPNO | 10 | |
| PROCNO | 1 | |
| Date_ | 20210622 | |
| Time | 11.17 | |
| INSTRUM | spect | |
| PROBHD | 5 mm PABBO BB- | |
| PULPROG | zg30 | |
| TD | 65536 | |
| SOLVENT | DMSO | |
| NS | 16 | |
| DS | 2 | |
| SWH | 10330.578 | Hz |
| FIDRES | 0.157632 | Hz |
| AQ | 3.1720407 | sec |
| RG | 128 | |
| DW | 48.400 | usec |
| DE | 6.50 | usec |
| TE | 298.3 | K |
| D1 | 1.00000000 | sec |
| TD0 | 1 | |

======== CHANNEL f1 ========

| | | |
|---|---|---|
| NUC1 | 1H | |
| P1 | 14.70 | usec |
| PL1 | -1.10 | dB |
| SFO1 | 500.1330885 | MHz |
| SI | 32768 | |
| SF | 500.1300000 | MHz |
| WDW | EM | |
| SSB | 0 | |
| LB | 0.30 | Hz |
| GB | 0 | |
| PC | 1.00 | |

C13CPD32 DMSO D:\\ abari 44

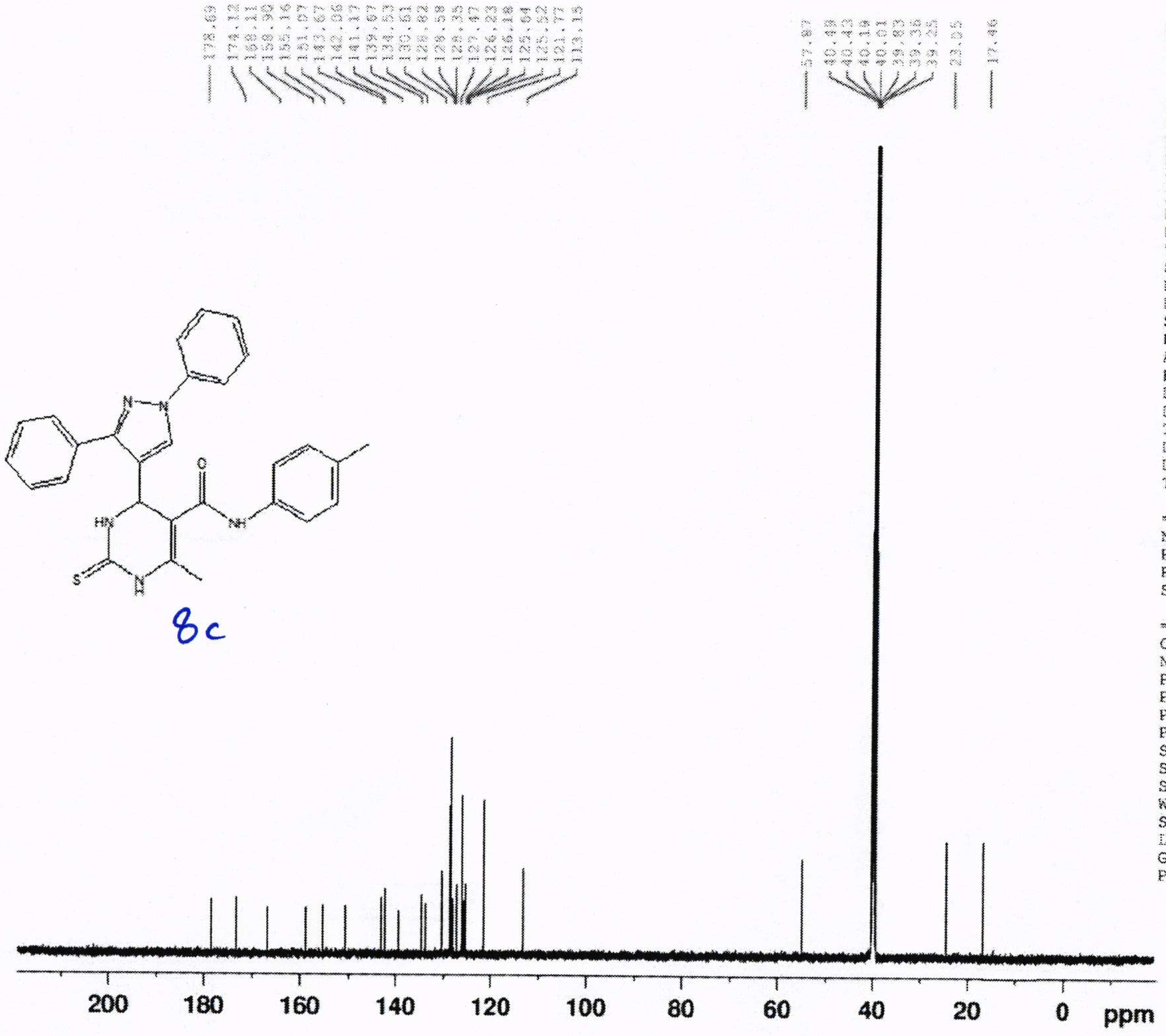


8c

BRUKER

| | |
|---|---|
| NAME | drshahat-15 |
| EXPNO | 11 |
| PROCNO | 1 |
| Date_ | 20210622 |
| Time | 12.07 |
| INSTRUM | spect |
| PROBHD | 5 mm PABBO BB- |
| PULPROG | zgpg30 |
| TD | 65536 |
| SOLVENT | DMSO |
| NS | 1165 |
| DS | 2 |
| SWH | 30030.029 Hz |
| FIDRES | 0.458222 Hz |
| AQ | 1.0912410 sec |
| RG | 4597.6 |
| DW | 16.650 usec |
| DE | 6.50 usec |
| TE | 299.2 K |
| D1 | 2.00000000 sec |
| D11 | 0.03000000 sec |
| TD0 | 1 |

======== CHANNEL f1 ========

| | |
|---|---|
| NUC1 | 13C |
| P1 | 20.00 usec |
| PL1 | -6.00 dB |
| SFO1 | 125.7703643 MHz |

======== CHANNEL f2 ========

| | |
|---|---|
| CPDPRG2 | waltz16 |
| NUC2 | 1H |
| PCPD2 | 80.00 usec |
| PL2 | -1.10 dB |
| PL12 | 13.44 dB |
| PL13 | 16.40 dB |
| SFO2 | 500.1320005 MHz |
| SI | 32768 |
| SF | 125.7577890 MHz |
| WDW | EM |
| SSB | 0 |
| LB | 1.00 Hz |
| GB | 0 |
| PC | 1.40 |

4-(1H-Indol-3-yl)-6-methyl-N-(4-nitrophenyl)-2-thioxo-1,2,3,4-tetrahydropyrimidine-5-carboxamide 9a

ON DMSO D:\\ abar

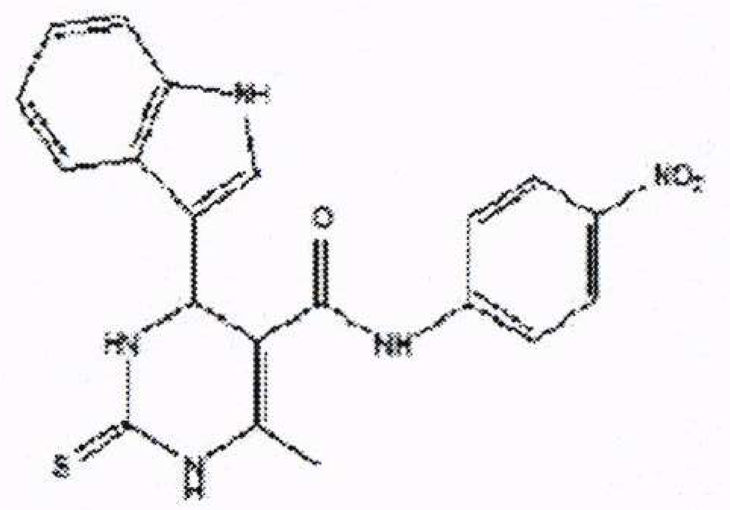


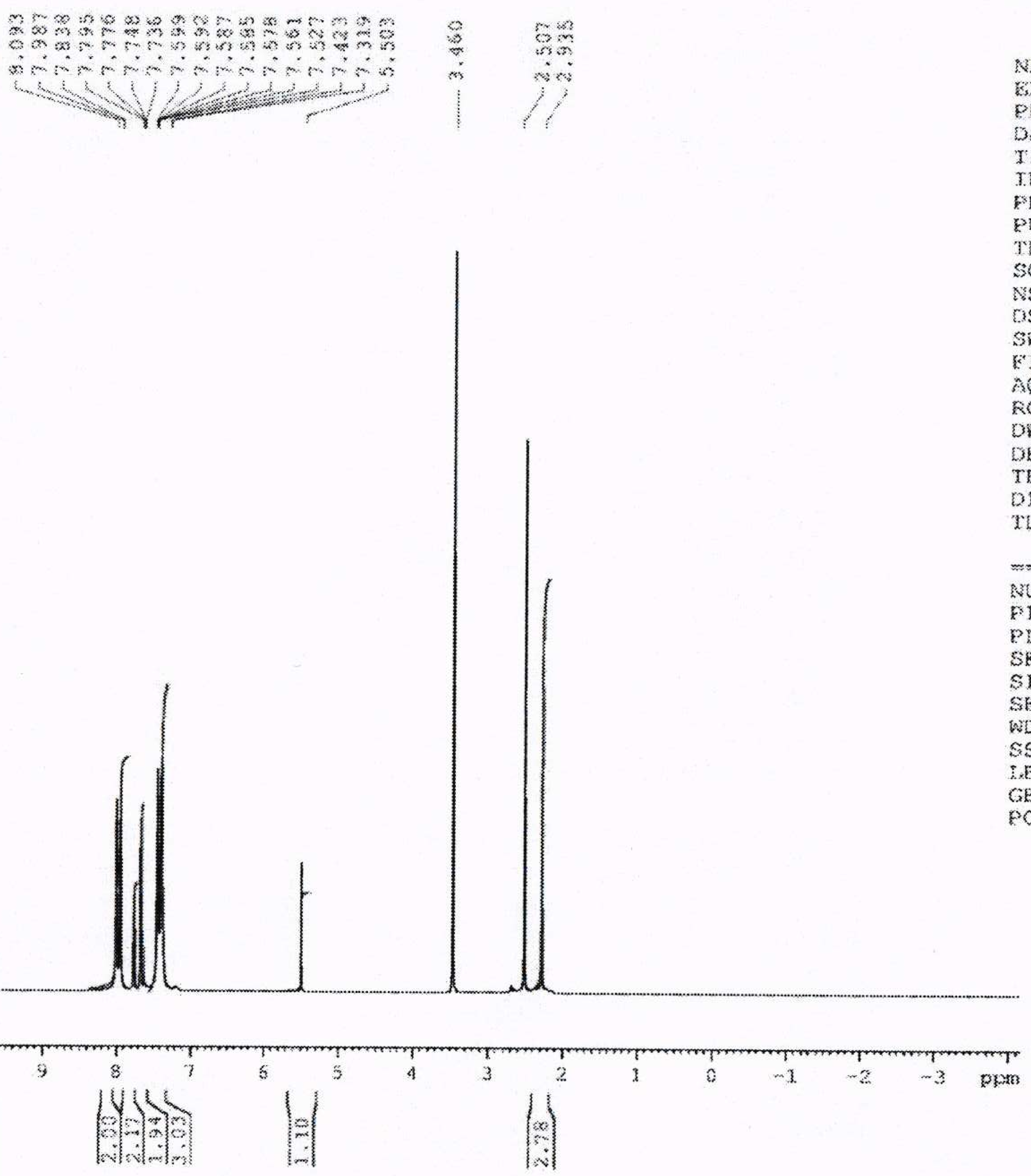


BRUKER

| | | |
|---|---|---|
| NAME | drshahat-17 | |
| EXPNO | 10 | |
| PROCNO | 1 | |
| Date_ | 20210622 | |
| Time | 18.59 | |
| INSTRUM | spect | |
| PROBHD | 5 mm PABBO BB- | |
| PULPROG | zg30 | |
| TD | 65536 | |
| SOLVENT | DMSO | |
| NS | 16 | |
| DS | 2 | |
| SWH | 10330.578 | Hz |
| FIDRES | 0.157632 | Hz |
| AQ | 3.1720407 | sec |
| RG | 90.5 | |
| DW | 48.400 | usec |
| DE | 6.50 | usec |
| TE | 298.3 | K |
| D1 | 1.00000000 | sec |
| TD0 | 1 | |

======== CHANNEL f1 ========

| | | |
|---|---|---|
| NUC1 | 1H | |
| P1 | 14.70 | usec |
| PL1 | -1.10 | dB |
| SFO1 | 500.1330885 | MHz |
| SI | 32768 | |
| SF | 500.1300000 | MHz |
| WDW | EM | |
| SSB | 0 | |
| LB | 0.30 | Hz |
| GB | 0 | |
| PC | 1.00 | |

C13CPD32 DMSO D:\\ abari 46

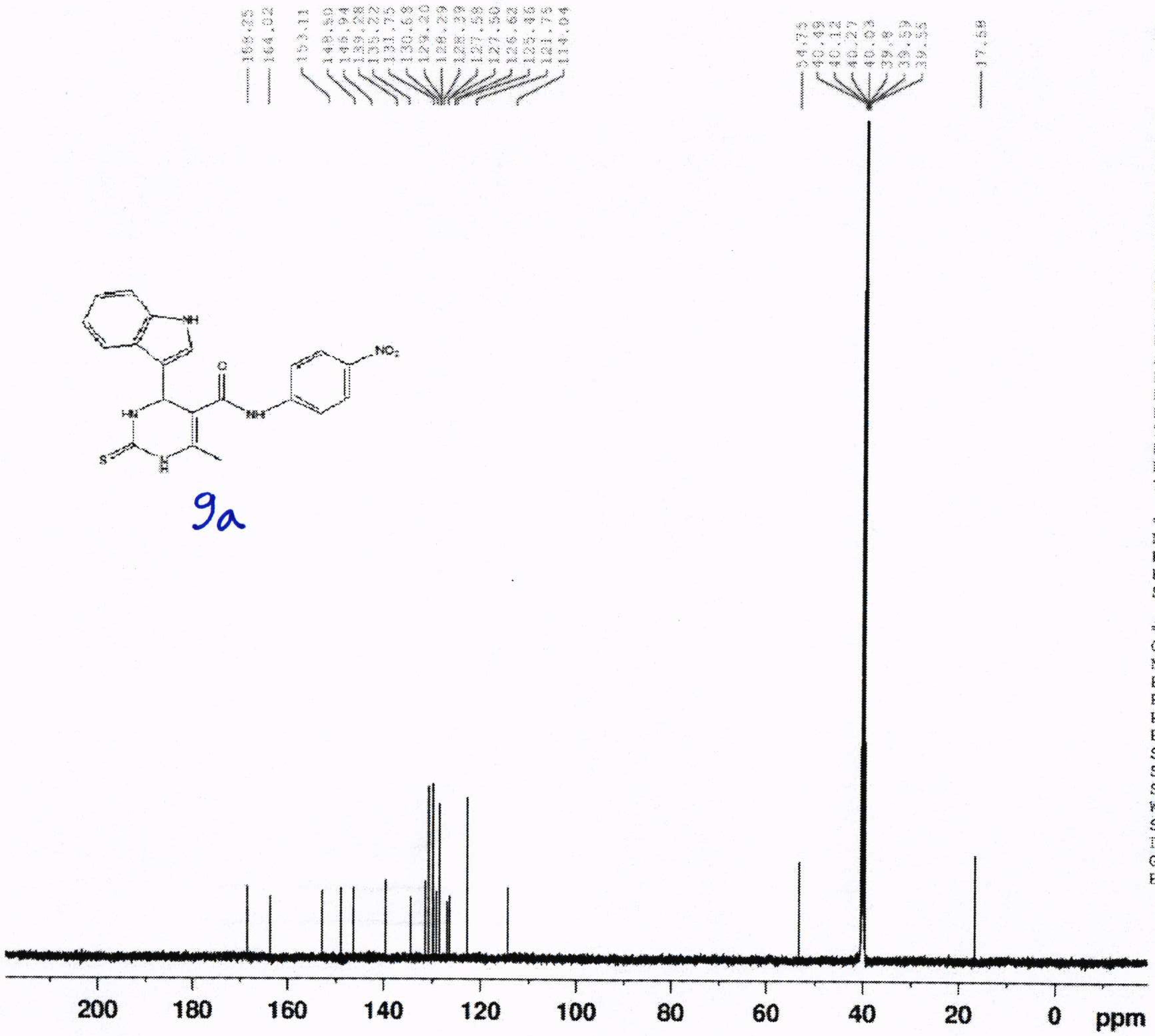


9a

BRUKER

| | |
|---|---|
| NAME | drshahat-17 |
| EXPNO | 11 |
| PROCNO | 1 |
| Date_ | 20210622 |
| Time | 20.47 |
| INSTRUM | spect |
| PROBHD | 5 mm PABBO BB- |
| PULPROG | zgpg30 |
| TD | 65536 |
| SOLVENT | DMSO |
| NS | 2000 |
| DS | 2 |
| SWH | 30030.029 Hz |
| FIDRES | 0.458222 Hz |
| AQ | 1.0912410 sec |
| RG | 14596.5 |
| DW | 16.650 usec |
| DE | 6.50 usec |
| TE | 299.1 K |
| D1 | 2.00000000 sec |
| D11 | 0.03000000 sec |
| TD0 | 1 |

======== CHANNEL f1 ========

| | |
|---|---|
| NUC1 | 13C |
| P1 | 20.00 usec |
| PL1 | -6.00 dB |
| SFO1 | 125.7703643 MHz |

======== CHANNEL f2 ========

| | |
|---|---|
| CPDPRG2 | waltz16 |
| NUC2 | 1H |
| PCPD2 | 80.00 usec |
| PL2 | -1.10 dB |
| PL12 | 13.44 dB |
| PL13 | 16.40 dB |
| SFO2 | 500.1320005 MHz |
| SI | 32768 |
| SF | 125.7577890 MHz |
| WDW | EM |
| SSB | 0 |
| LB | 1.00 Hz |
| GB | 0 |
| PC | 1.40 |

4-(2-Chloroquinolin-3-yl)-6-methyl-N-(4-nitrophenyl)-2-thioxo-1,2,3,4-tetrahydropyrimidine-5-carboxamide 9b

ON DMSO D:\\ abar

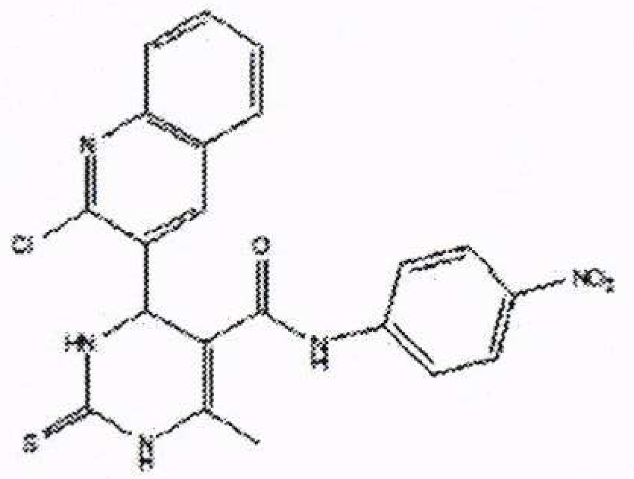


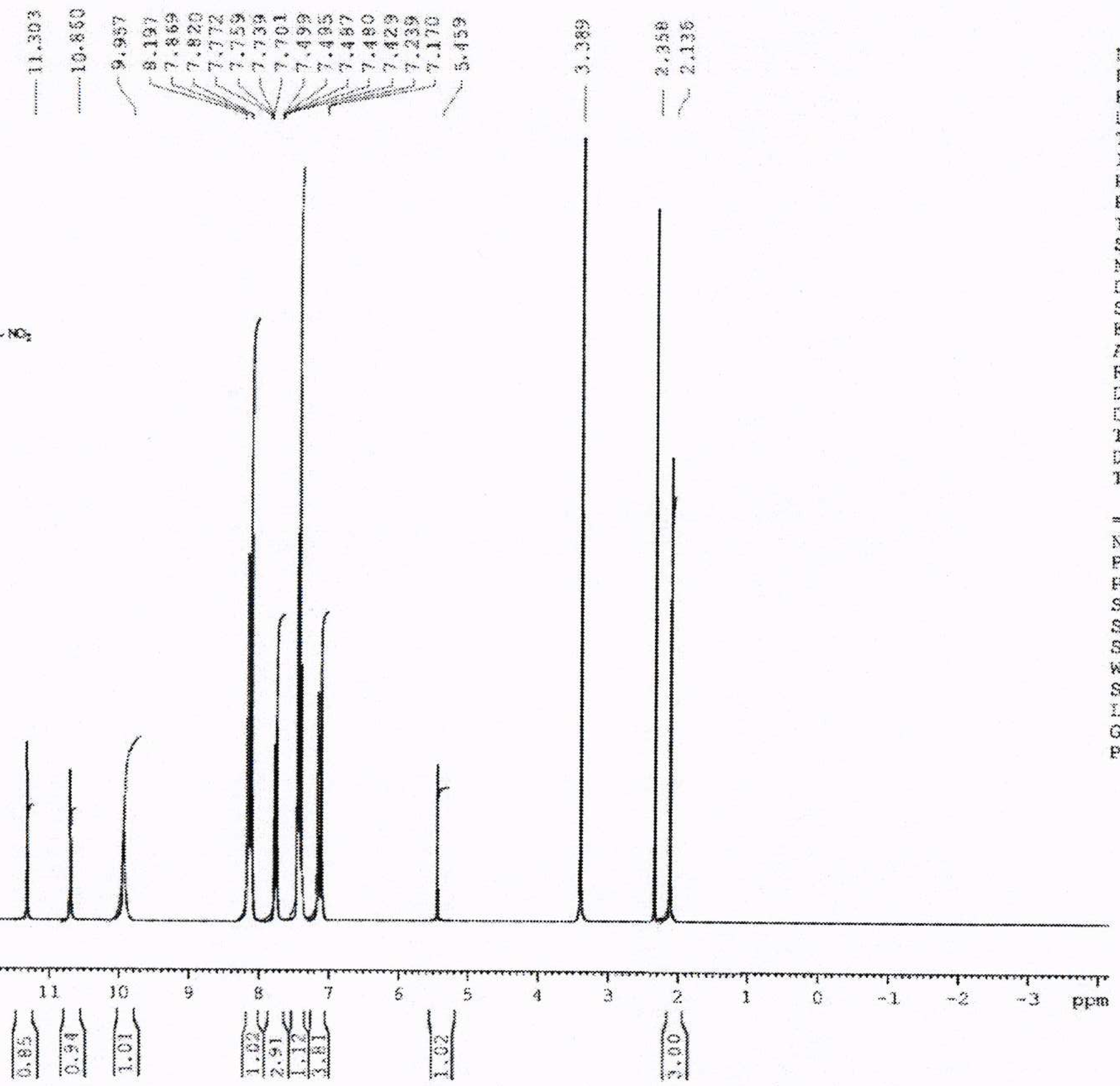


BRUKER

| | | |
|---|---|---|
| NAME | drshahat-16 | |
| EXPNO | 10 | |
| PROCNO | 1 | |
| Date_ | 20210622 | |
| Time | 18.59 | |
| INSTRUM | spect | |
| PROBHD | 5 mm PABBO BB- | |
| PULPROG | zg30 | |
| TD | 65536 | |
| SOLVENT | DMSO | |
| NS | 16 | |
| DS | 2 | |
| SWH | 10330.578 | Hz |
| FIDRES | 0.157632 | Hz |
| AQ | 3.1720407 | sec |
| RG | 90.5 | |
| DW | 48.400 | usec |
| DE | 6.50 | usec |
| TE | 298.3 | K |
| D1 | 1.00000000 | sec |
| TD0 | 1 | |

======= CHANNEL f1 =======

| | | |
|---|---|---|
| NUC1 | 1H | |
| P1 | 14.70 | usec |
| PL1 | -1.10 | dB |
| SFO1 | 500.1330885 | MHz |
| SI | 32768 | |
| SF | 500.1300000 | MHz |
| WDW | EM | |
| SSB | 0 | |
| LB | 0.30 | Hz |
| GB | 0 | |
| PC | 1.00 | |

C13CPD32 DMSO D:\\ abari 46

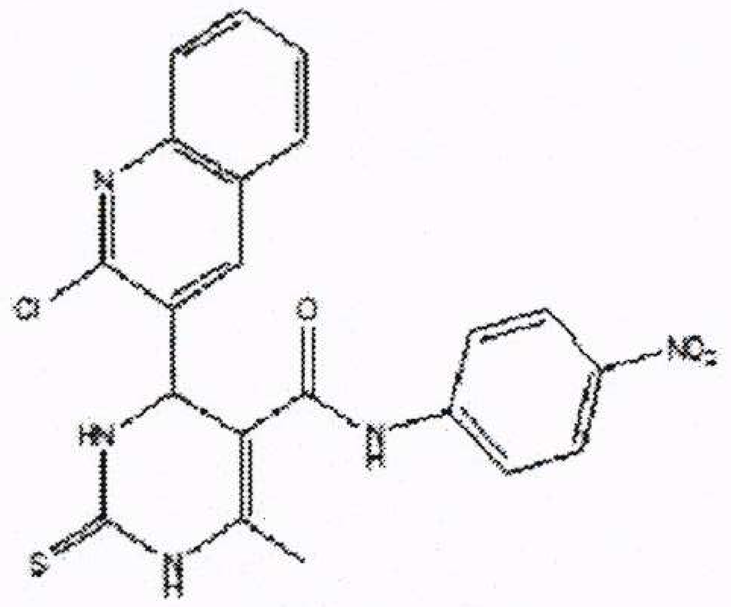

9b

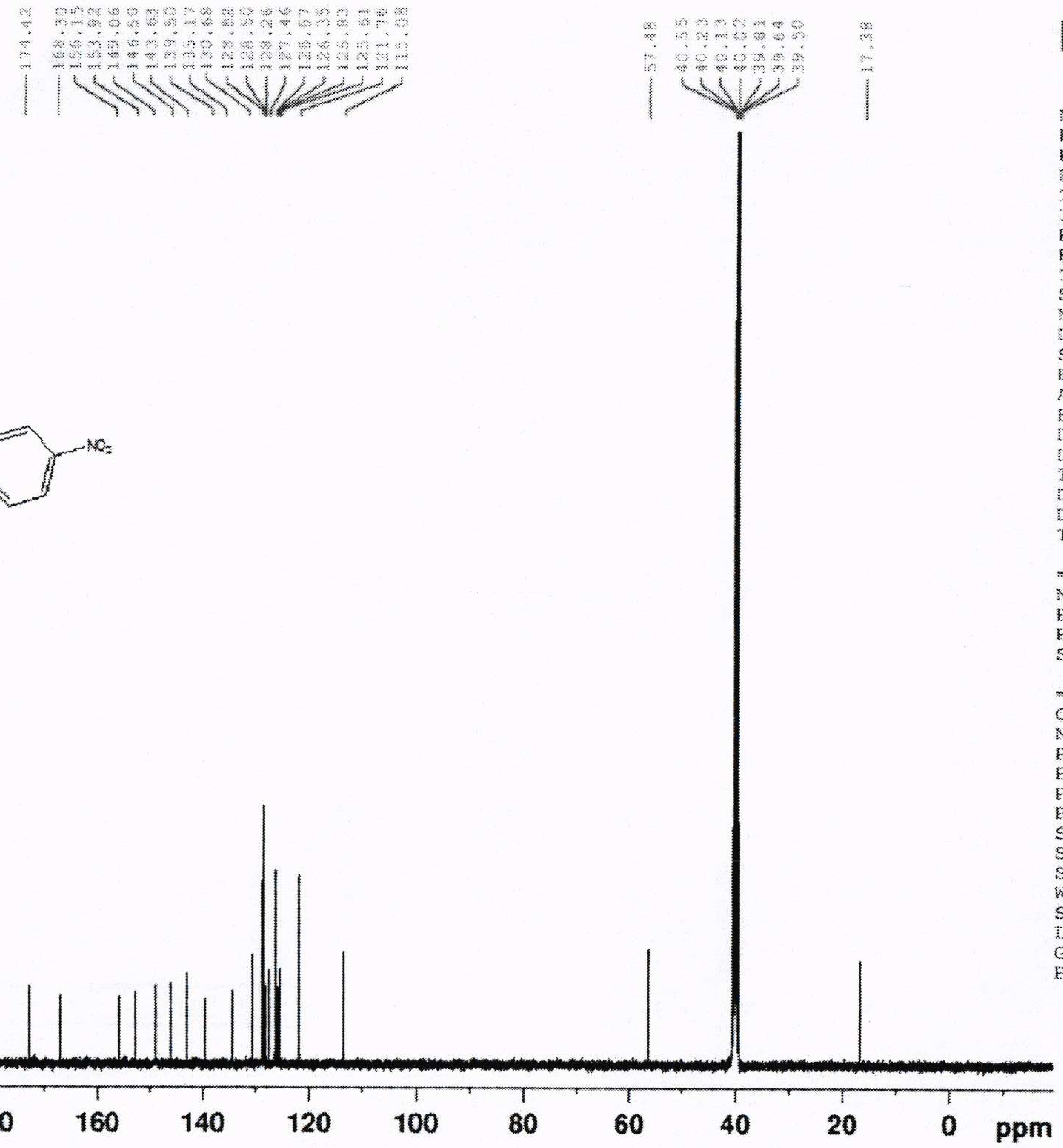


BRUKER

| | |
|---|---|
| NAME | drshahat-16 |
| EXPNO | 11 |
| PROCNO | 1 |
| Date_ | 20210622 |
| Time | 20.47 |
| INSTRUM | spect |
| PROBHD | 5 mm PABBO BB- |
| PULPROG | zgpg30 |
| TD | 65536 |
| SOLVENT | DMSO |
| NS | 2000 |
| DS | 2 |
| SWH | 30030.029 Hz |
| FIDRES | 0.458222 Hz |
| AQ | 1.0912410 sec |
| RG | 14596.5 |
| DW | 16.650 usec |
| DE | 6.50 usec |
| TE | 299.1 K |
| D1 | 2.00000000 sec |
| D11 | 0.03000000 sec |
| TD0 | 1 |

======== CHANNEL f1 ========

| | |
|---|---|
| NUC1 | 13C |
| P1 | 20.00 usec |
| PL1 | -6.00 dB |
| SFO1 | 125.7703643 MHz |

======== CHANNEL f2 ========

| | |
|---|---|
| CPDPRG2 | waltz16 |
| NUC2 | 1H |
| PCPD2 | 80.00 usec |
| PL2 | -1.10 dB |
| PL12 | 13.44 dB |
| PL13 | 16.40 dB |
| SFO2 | 500.1320005 MHz |
| SI | 32768 |
| SF | 125.7577890 MHz |
| WDW | EM |
| SSB | 0 |
| LB | 1.00 Hz |
| GB | 0 |
| PC | 1.40 |

4-(1,3-Diphenyl-1H-pyrazol-4-yl)-6-methyl-N-(4-nitrophenyl)-2-thioxo-1,2,3,4-tetrahydropyrimidine-5-carboxamide 9c

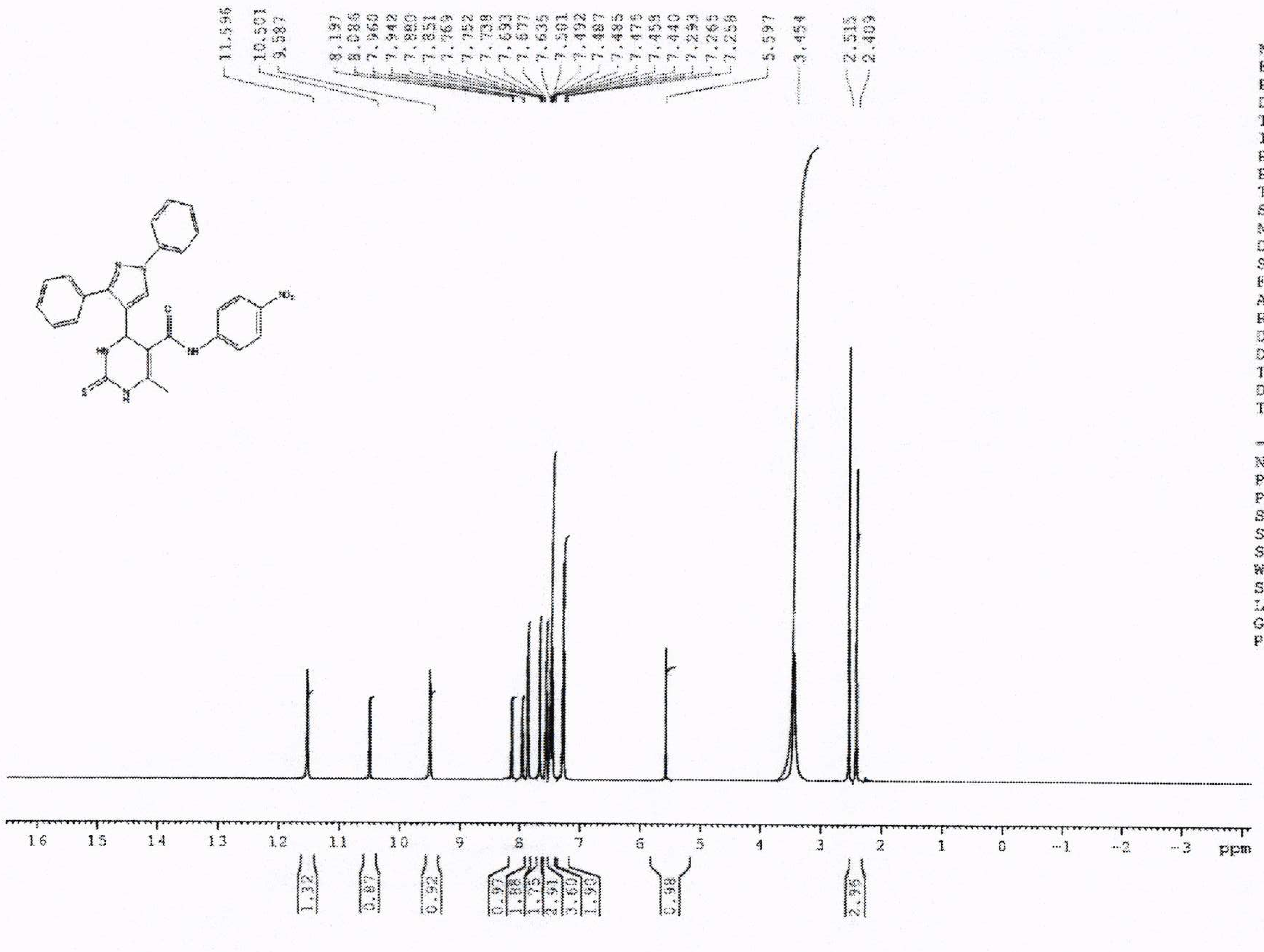


NAME drshahat-18
EXPNO 10
PROCNO 1
Date_ 20210622
Time 11.17
INSTRUM spect
PROBHD 5 mm PABBO BB-
PULPROG zg30
TD 65536
SOLVENT DMSO
NS 16
DS 2
SWH 10330.578 Hz
FIDRES 0.157632 Hz
AQ 3.1720407 sec
RG 128
DW 48.400 usec
DE 6.50 usec
TE 298.3 K
D1 1.00000000 sec
TD0 1

======== CHANNEL f1 ========
NUC1 1H
P1 14.70 usec
PL1 -1.10 dB
SFO1 500.1330885 MHz
SI 32768
SF 500.1300000 MHz
WDW EM
SSB 0
LB 0.30 Hz
GB 0
PC 1.00

C13CPD32 DMSO D:\\ abari 44

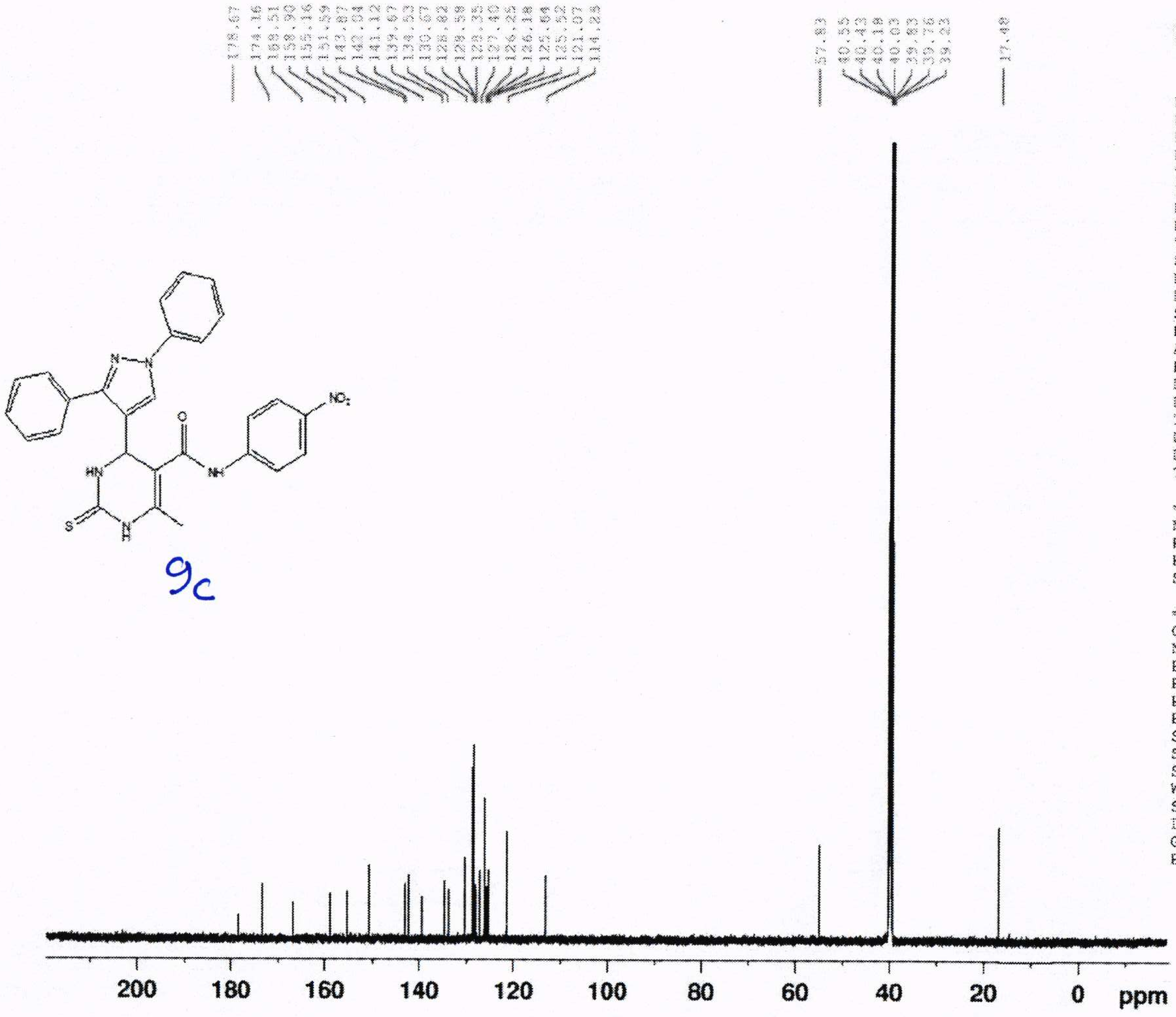


9c

BRUKER

| | |
|---|---|
| NAME | drshahat-18 |
| EXPNO | 11 |
| PROCNO | 1 |
| Date_ | 20210622 |
| Time | 12.07 |
| INSTRUM | spect |
| PROBHD | 5 mm PABBO BB- |
| PULPROG | zgpg30 |
| TD | 65536 |
| SOLVENT | DMSO |
| NS | 1165 |
| DS | 2 |
| SWH | 30030.029 Hz |
| FIDRES | 0.458222 Hz |
| AQ | 1.0912410 sec |
| RG | 4597.6 |
| DW | 16.650 usec |
| DE | 6.50 usec |
| TE | 299.2 K |
| D1 | 2.00000000 sec |
| D11 | 0.03000000 sec |
| TD0 | 1 |

======== CHANNEL f1 ========

| | |
|---|---|
| NUC1 | 13C |
| P1 | 20.00 usec |
| PL1 | -6.00 dB |
| SFO1 | 125.7703643 MHz |

======== CHANNEL f2 ========

| | |
|---|---|
| CPDPRG2 | waltz16 |
| NUC2 | 1H |
| PCPD2 | 80.00 usec |
| PL2 | -1.10 dB |
| PL12 | 13.44 dB |
| PL13 | 16.40 dB |
| SFO2 | 500.1320005 MHz |
| SI | 32768 |
| SF | 125.7577890 MHz |
| WDW | EM |
| SSB | 0 |
| LB | 1.00 Hz |
| GB | 0 |
| PC | 1.40 |
